# Genomic dissection of methane emission traits in cattle: A meta-GWAS and heritability analysis across populations

**DOI:** 10.1371/journal.pone.0344752

**Published:** 2026-04-10

**Authors:** Sare Golpasand, Navid Ghavi Hossein-Zadeh, Shahrokh Ghovvati

**Affiliations:** Department of Animal Science, Faculty of Agricultural Sciences, University of Guilan, Rasht, Iran; Ataturk University: Ataturk Universitesi, TÜRKIYE

## Abstract

Enteric methane emissions from ruminants represent a significant contributor to agricultural greenhouse gases, necessitating precise genetic tools to guide mitigation strategies. This study aimed to identify genomic regions and estimate heritability parameters associated with methane-related traits in cattle through an integrated meta-analytical framework. The meta-analysis of the genome-wide association studies (meta-GWAS) was carried out with the METAL software, combining SNP level data extracted from published studies. Simultaneously, a distinct random effects meta-analysis of genomic and pedigree-based heritability estimates was performed using Comprehensive Meta-Analysis software. Functional analysis of the post-GWAS, including: Gene Ontology, KEGG, and network-based enrichment analysis, was also performed to describe the biological context of significant genes. The meta-GWAS identified 74 significant SNPs that were significant for the traits of methane, which are related to 113 candidate genes. Functional enrichment analyses revealed pathways related to metabolism, immune response, ion transport, and host–microbiome interactions. The KEGG metabolic pathway emerged as a highly enriched term, encompassing key genes such as: *ALDH7A1*, *CYP51A1*, *P4HA2*, and *SHPK*, which are involved in amino acid catabolism, lipid processing, and redox regulation functions critical to energy balance and digestive efficiency. Network analysis with Cytoscape has revealed *TRPV3, TRPV1, ANK3, PKD2* and *SHPK* as network hub genes. Heritability meta-analysis indicated that methane production exhibited the moderate genomic (h^2^ = 0.296) and pedigree-based (h^2^ = 0.299) heritability estimations, and methane yield was also found to have moderate and high heritability. The findings highlight the potential for methane-related traits as viable targets for genetic selection. This research demonstrates the value of integrating functional genomics and quantitative genetic approaches to enhance understanding of the biological and heritable components of methane emissions, providing a robust foundation for an environmentally sustainable livestock breeding program.

## Introduction

With the growing global concern about climate change, methane has emerged as a critical target in mitigation strategies. It has a high global warming potential and a relatively short atmospheric lifespan that makes it a unique point of focus to achieve accelerated benefits of climate. Despite technological progress in controlling industrial methane emissions, enteric fermentation in ruminant livestock remains a major contributor to global methane emissions [1]. Over the past 130 years, methane emissions from livestock, particularly from ruminants, particularly cattle and buffalo have increased more than fourfold, and the largest contributors were non-dairy and dairy cattle [1]. Thus, effective mitigation strategies in the livestock industry must integrate nutritional interventions, genetic selection, and improvements in production efficiency to reduce environmental impact without affecting animal health, productivity, or system sustainability [2–7].

Genome-wide association studies (GWAS) have become a powerful approach to genetic variants and markers associated with economically important and complex traits in livestock, particularly in cattle [8–10]. Statistical modeling and high-density single-nucleotide polymorphism (SNP) arrays have enabled the identification of genotype-phenotype associations across populations, enhancing our knowledge about the molecular architecture of complex traits Moreover, GWAS summary statistics are useful as inputs to meta-analyses, fine-mapping and the generation of genomic prediction models [11–13].

In recent years, GWAS have been increasingly applied to sustainability-related traits such as: disease resistance, heat tolerance, feed efficiency, and methane emission in ruminants [14–16]. GWAS also provide a genetic basis to refine the selection indices and improve the precision of genomic evaluations in livestock breeding programs by discovering significant SNPs [17]. Several GWAS studies have been conducted to investigate methane-related traits in livestock [16,18–20].

In addition to the identification of significant genomic regions, a precise and quantitative understanding the genetic parameters underlying methane emission-related traits, including their heritability and genetic correlations with key economic traits, is essential for developing sustainable breeding strategies. Recent studies in ruminants confirm that methane emissions show significant heritable variation without having an adverse impact on productivity [21]. Methane emission is a measurable environmental trait that can be incorporated directly or indirectly into selection indices. Thus gradual minimization of the environmental footprint of livestock production is a viable and sustainable approach to environmental mitigation through genetic selection [22]. However, heritability estimates for methane traits have been reported to differ widely because of the variation in population size, breed composition, measurement methods, and models of analysis. Integrating results through meta-analysis provides a robust statistical framework for synthesizing these heterogeneous findings. Specifically, random-effects models consider both within- and between-study variation, and the estimates are more representative of actual genetic effects in different populations [23–26]

Additionally, integrative genomic methods that involve the combination of GBLUP and GWAS have been found to be useful in estimating heritability and also identifying candidate genes. For instance, genomic analyses in Nellore cattle have identified several genes that regulate rumen microbiota, lipid metabolism, and fatty acid biosynthesis that contribute to genetic variation in methane emissions [18].

Integrating GWAS results with reliable heritability estimates, especially in the context of genomic selection, has a high potential for developing selection indices and breeding strategies that would reduce greenhouse gas emissions in livestock [27,28]. This effort is reinforced by meta-analysis, which combines results from independent studies, thereby increasing the statistical power to detect actual genetic associations and improving the accuracy and reliability of results [13,29]. The recent methodological developments in statistical for genomic meta-analysis have significantly increased the ability to detect subtle genetic effects and have provided to clarify the missing heritability that is frequently lost in individual studies [30]. Furthermore, large-scale international collaborations and research consortia, which increase sample sizes and include genetically diverse populations have enhanced the discovery of novel loci and improved our understanding of complex traits [31]. Recognizing the urgent necessity of environmentally sustainable livestock systems, the current study combines two complementary methods, a meta-analysis of published GWAS findings and a random-effects meta-analysis of genomic and pedigree-based heritability estimates. This integrative framework enhances the genetic knowledge of the methane-related traits in cattle by synthesizing information in a wide range of populations and study designs, which increases statistical power, decreases study-specific bias, and improves the ability to identify important genomic regions associated to methane emissions. The findings of this study are useful for designing environmentally sustainable breeding strategies to reduce the environmental impact of livestock production.

## Materials and methods

### Search strategy and data selection criteria

**Fig 1:**
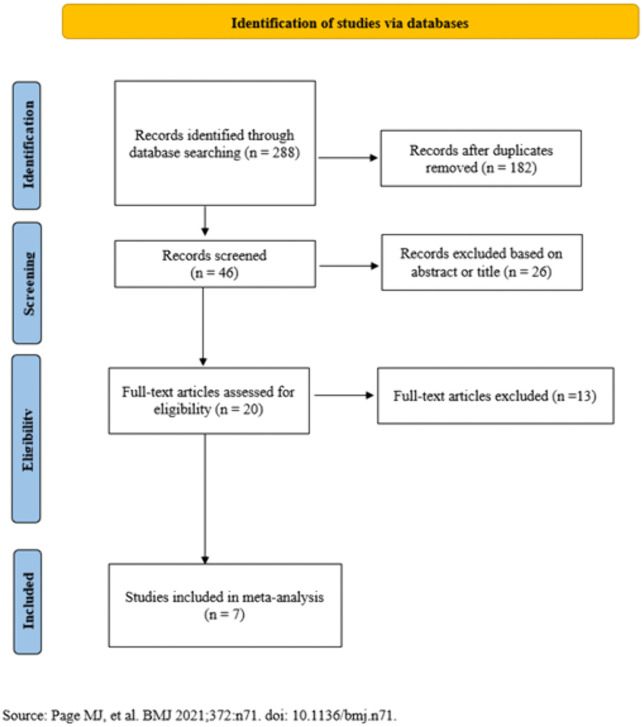
Flow diagram representing the studies incorporated into the GWAS meta-analysis by the PRISMA approach.

**Fig 2:**
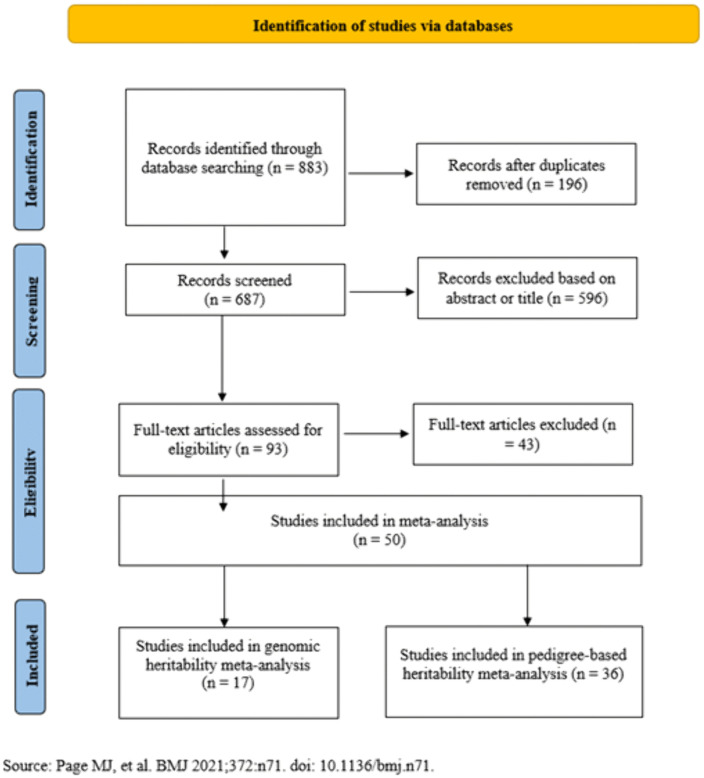
Flow diagram representing the studies incorporated into the heritability meta-analysis by the PRISMA approach.

A systematic and comprehensive review of the literature on GWAS, genomic heritability, and pedigree-based heritability studies related to methane-related traits in cattle was performed based on the PRISMA (Preferred Reporting Items for Systematic Reviews and Meta-Analyses) guidelines [32] (Figs 1 and 2 and S1 Checklist).

The last searches of the database were carried out on 30 September 2025 to identify all relevant studies. Literature searches were performed using multiple databases, including Google Scholar (https://scholar.google.com), PubMed (https://pubmed.ncbi.nlm.nih.gov), Scopus (https://www.scopus.com), Web of Science (https://www.webofscience.com), and ResearchGate (https://www.researchgate.net) databases.

To guide the search strategy and ensure a structured and focused retrieval process, the Population, Intervention, Comparison, and Outcome (PICO) framework was adopted [33]. Three distinct categories of analysis were considered under this framework; For GWAS meta-analysis, the population included dairy and beef cattle breeds evaluated for methane-related traits. The intervention involved GWAS employing SNP marker arrays. Comparisons were made across different SNP markers, cattle populations, or multiple studies. The outcome of interest was the identification of SNPs significantly associated with methane-related traits. The second category focused on studies reporting genomic heritability estimates. The population consisted of cattle breeds assessed for methane-related traits. Interventions involved genomic heritability estimation using SNP-based genomic relationship models, such as genomic best linear unbiased prediction (GBLUP), BayesC, or single-step genomic approaches. Comparisons were drawn across different breeds, estimation models, or population structures. The outcome was the estimation of genomic heritability (h²_g_) for methane production, methane yield, or associated traits. In the pedigree-based heritability analysis, the target population included cattle breeds with available phenotypic data for methane-associated traits. Studies estimating pedigree-based heritability using Bayesian inference and restricted maximum likelihood (REML) estimation methods in animal models with additive genetic relationship matrices have been included in the interventions. Comparisons were made across different breeds, statistical models, or estimation methodologies. The outcome was pedigree-based heritability estimates (h²_a_) for methane production and related traits.

Additionally, the following strategically combined search terms ensured comprehensive retrieval of relevant studies:

- (“GWAS” OR “genome-wide association”) AND (“methane production” OR “methane emission” OR “methane yield” OR “residual methane” OR “methane intensity” OR “methane concentration” OR “enteric methane”) AND (“cattle” OR “cow” OR “bovine”)
- (“genomic heritability” OR “heritability” OR “genetic parameter”) AND (“methane production” OR “methane emission” OR “methane yield” OR “residual methane” OR “methane intensity” OR “methane concentration” OR “enteric methane”) AND (“cattle” OR “cow” OR “bovine”)

No restrictions were imposed on the publication date to ensure maximal coverage of all relevant studies.

In total, the titles of 46 articles were screened, of which 20 articles were assessed in full text for eligibility based on the use of GWAS for methane-related traits and the availability of SNP-level results, including genomic coordinates and P-values. After a thorough evaluation, 7 articles met the inclusion criteria and were retained for the meta-analysis. From the identified studies, data including study title, SNP identifier (marker name), chromosome number, genomic position, P-value, and sample size were extracted and compiled into an Excel file. Additionally, 687 articles related to the heritability estimates for methane traits were initially screened. Based on the title review, 93 articles were deemed relevant and were assessed in full text. After excluding 43 studies due to insufficient or incomplete information, 50 articles were ultimately included for heritability estimates in the meta-analysis. Additional file 1 S1 Table provides a summary of the total number of records from studies included in the heritability meta-analysis. It presents key metadata for each study, including the reference, country or region of origin, cattle breed, type of genetic relationship matrix employed (pedigree or genomic-based), statistical method used, and the analytical model implemented for heritability estimation.

### GWAS meta-analysis

For the meta-analysis, marker names, P-values, and sample sizes extracted from the selected GWAS articles were compiled into text files compatible with METAL software (version 2011-03-25), a command-line tool for GWAS meta-analytic studies [34]. The analysis was conducted using the sample-size–weighted Z-score method implemented in METAL. Due to the lack of consistent reference allele information across the included studies, it was assumed that the effect directions were consistent for all markers. Following the meta-analysis, genome-wide significance was assessed using the Bonferroni correction. The significance threshold was determined by dividing the conventional alpha level (α = 0.05) by the total number of SNPs included in the meta-analysis.

### Downstream functional annotation, gene ontology, and network analysis

Significant SNPs identified through meta-analysis were examined in order to determine any genes that could be related to methane emission traits. Genomic position and variant identifiers and data were retrieved and confirmed by Ensembl BioMart (https://asia.ensembl.org/info/website/index. html) and Variant Effect Predictor (VEP) database (https://asia.ensembl.org/Tools/VEP). Additionally, genes associated with SNPs reported in previous GWAS studies were incorporated to strengthen the candidate gene list. This integrative approach resulted in the identification of 113 genes (S2 Table), forming the foundation for subsequent post-GWAS analyses. Network analysis employs mathematical and computational methodologies to investigate the structure, function, and dynamics of interconnected systems. This approach, commonly referred to as network science, focuses on the systematic analysis of complex systems composed of interacting elements. These elements, referred to as nodes, are connected through edges or links that represent various types of relationships, collectively forming a network [35]. Protein-protein interaction (PPI) network analysis enables the exploration of functional relationships among proteins and provides insight into their roles within broader biological systems. To explore the interactions among the genes, a PPI network was constructed using the STRING database (https://string-db.org/) and visualized with Cytoscape software [36]. The CytoHubba plug-in was applied using the maximal clique centrality (MCC) algorithm to identify the five key hub genes based on their degree of centrality within the network. Functional enrichment analysis was conducted using the ClueGO and CluePedia plugins within Cytoscape [37,38]. The analysis included all genes associated with significant SNPs and was carried out across the three Gene Ontology (GO) categories: biological process (BP), molecular function (MF), and cellular component (CC). In order to confirm and complement these results, a secondary GO-based enrichment analysis was conducted using the BiNGO plug-in in Cytoscape [39]. The default parameters of BiNGO were used, using the same gene list and GO categories (BP, MF, CC), with a significance threshold of P < 0.05 after FDR correction. The created hierarchical GO tree using BiNGO was color-coded based on the statistical significance to facilitate interpretation of enriched biological functions.

To further refine the network analysis, the genes with significant SNPs were mapped to their human orthologs, and a gene network analysis for 86 genes was performed with GeneMANIA in Cytoscape [40]. This tool constructs a comprehensive gene interaction network using various data sources, including co-expression, co-localization, physical interactions, shared protein domains, predicted functional relationships, and literature-based associations to construct a comprehensive gene interaction network. Finally, to obtain a broader understanding of gene function, enrichment analyses were performed using the DAVID web tool (https://davidbioinformatics.nih.gov/) that enables enrichment analysis across three main functional categories: biological processes (BP), cellular components (CC), and molecular functions (MF). In addition, pathway enrichment based on the KEGG database was conducted to identify key biological and metabolic pathways potentially involved in methane emission.

### Meta-analysis of genomic and pedigree-based heritability estimates

Genomic and pedigree-based heritability estimates were meta-analyzed separately using Comprehensive Meta-Analysis (CMA) software version 3.0 [41]. The analysis of genomic heritability estimates was limited to two traits: methane production (METP) and methane concentration (METC). Eligible studies were systematically searched and identifiable estimates of heritability and their standard errors were obtained. In cases where standard errors for heritability estimates were not reported in the articles, these values were estimated using the combined variance method [42]. The formula for calculating the predicted standard error for articles that lacked this information is defined as follows:


$${SE}_{ij}=\sqrt{\frac{\left(\frac{\sum_{k=\text{1}}^{K}\overset{\text{2}}{\underset{ik}{s}}\overset{\text{2}}{\underset{ik}{n}}}{\sum_{k=\text{1}}^{K}n_{ik}}\right)}{{n^{\prime }}_{ij}}}$$

In this equation, SE_ij_ denotes the estimated standard error associated with the genetic parameter reported for the *i*th trait in the *j*th article, in which the original standard error was not provided. S_ik_ represents the observed standard error for the estimate of the *i*th trait in the *k*th article, which includes both the parameter estimate and its standard error. n_ik_ refers to the number of records used to derive the parameter estimate in the *k*th study, and n′ij is the number of records used to predict the published parameter estimate for the *i*th trait in the *j*th article that has not reported the standard error.

The extracted data were subsequently imported into CMA for statistical analysis. Given the expected heterogeneity across studies, a random-effects model was employed. Following the meta-analysis, the pooled heritability estimate and its associated standard error were calculated.

### Evaluation of publication bias

To evaluate the presence of publication bias, Egger’s linear regression test was employed. When statistical evidence of bias was detected (P < 0.10), the Trim-and-Fill method was applied to detect the number of potentially missing studies [43]. A Funnel plot was also generated and visually inspected to assess the symmetry of the effect size distribution. The results were analyzed and interpreted in the context of between-study heterogeneity and overall effect size estimation. After estimating the number of missing studies, the imputed values were incorporated into the model to recalculate the weighted mean effect size and its associated variance. It should be noted that in the presence of significant heterogeneity across studies (Q test with P < 0.10), the validity of publication bias tests may be compromised, potentially leading to false positive claims [44].

## Results

### GWAS meta-analysis

This meta-analysis integrated genomic data, specifically SNP positions, chromosomal locations, and p-values, extracted from relevant studies identified through a systematic literature review (Table 1).

**Table 1:** Characteristics of GWAS papers included in databases for performing a meta-analysis.

Reference	Number of Animals	Number of Significant SNPs	Breed
Souza et al. [18]	743	11	Nellore cattle
Lekamp et al. [16]	802	28	crossbred beef steers
Manzanilla-Pech et al. [45]	1844	21	Danish Holstein cattle
Jalil Sarghale et al. [19]	150	9	Iranian Holstein cattle
Jalil Sarghale et al. [46]	150	5	Iranian Holstein cattle
Calderon-Chagoya et al. [47]	280	46	Dairy cattle (Simmental, Holstein, or Brown Swiss)
Pszczola et al. [20]	287	50	Polish Holstein-Friesian

SNP: Single Nucleotide Polymorphism.

Due to the lack of reported SNP effect sizes in the original studies, the combined statistical analyses were based exclusively on the p-values derived from each study. After the meta-analysis, 74 SNPs were found to be statistically significant (P-value < 0.0003), and it was found that they are association with methane emission traits. The number of significant SNPs per chromosome is summarized in Table 2, offering an overview of their genome-wide distribution patterns associated with methane emission traits.

**Table 2:** The number of significant SNPs per chromosome.

Chromosome number	Length of chromosome (bps)	Number of SNPs
1	158,534,110	8
2	136,231,102	1
3	121,005,158	2
4	120,000,601	2
5	120,089,316	5
6	117,806,340	1
7	110,682,743	7
8	113,319,770	1
9	105,454,467	2
10	103,308,737	10
11	106,982,474	1
12	87,216,183	3
13	83,472,345	3
14	82,403,003	2
15	85,007,780	2
16	81,013,979	1
17	73,167,244	6
18	65,820,629	1
19	63,449,741	3
20	71,974,595	3
23	52,498,615	2
24	62,317,253	2
28	45,940,150	3

The Table 3 provides detailed information on the identified SNPs, including their chromosomal positions and statistical significance.

**Table 3:** Detailed information of significant SNPs identified in GWAS Meta-analysis.

Marker name	CHR	Genomic position	Allele 1	Allele 2	Weight^*^	Z-score	P-value
1_54481566	1	54481566	T	G	150	5.137	2.79E-07
BovineHD0100039506	1	138388705	T	G	280	4.553	5.30E-06
BovineHD0100033007	1	116746240	A	C	280	4.36	1.30E-05
BovineHD0100026381	1	92730269	T	C	280	4.107	4.00E-05
BovineHD0100035695	1	126404986	A	G	280	4.096	4.20E-05
BovineHD0100024203	1	84485319	A	C	280	3.927	8.60E-05
BovineHD0100022218	1	77090546	T	C	280	3.643	0.00027
BovineHD0100025559	1	89815855	A	G	280	3.624	0.00029
ARS-BFGL-NGS-116634	2	107318080	A	G	150	4.866	1.14E-06
rs110220315	3	86090597	A	G	2406	6.75	1.48E-11
rs110058749	3	88164394	G	A	2406	6.513	7.38E-11
BovineHD0300026630	3	92487386	A	G	280	3.867	0.00011
4:115131249	4	115131249	G	C	150	6.351	2.14E-10
rs134296722	4	104579265	T	C	1604	5.197	2.02E-07
BovineHD0400033637	4	115960201	A	G	280	3.633	0.00028
rs109244569	5	21516232	T	G	2406	6.935	4.06E-12
rs110309656	5	21475995	C	A	2406	6.935	4.06E-12
rs42740586	5	1870477	G	A	2406	6.585	4.56E-11
rs137645685	5	60495101	G	A	2406	6.516	7.22E-11
BovineHD0500011829	5	44357278	A	C	280	4.265	2.00E-05
BovineHD0600033597	6	117942392	T	C	150	5.304	1.13E-07
rs43508672	7	22191462	A	G	2406	7.581	3.44E-14
rs43508661	7	22204260	A	G	2406	7.581	3.44E-14
rs43508667	7	22197623	A	G	2406	7.581	3.44E-14
rs29023390	7	22202959	G	A	2406	7.581	3.44E-14
rs43508669	7	22195580	T	C	2406	7.581	3.44E-14
rs43509246	7	22328341	C	T	2406	7.581	3.44E-14
rs43141114	7	22268814	G	A	2406	7.581	3.44E-14
rs41621748	8	56479469	G	A	2406	6.503	7.87E-11
BovineHD080011543	8	38725165	A	C	150	4.903	9.43E-07
rs43605790	9	89807465	G	A	2406	9.723	2.41E-22
BovineHD0900021600	9	77448787	A	G	280	4.085	4.40E-05
rs132818032	10	67219330	A	G	743	5.508	3.63E-08
rs109315063	10	67275755	G	A	743	5.508	3.63E-08
rs134213606	10	67236195	G	A	743	5.508	3.63E-08
rs135715112	10	67262107	G	A	743	5.508	3.63E-08
rs135939893	10	67227401	A	G	743	5.508	3.63E-08
rs133164059	10	67254747	G	A	743	5.508	3.63E-08
rs109694327	10	67248322	A	G	743	5.508	3.63E-08
rs136661248	10	67231936	G	T	743	5.508	3.63E-08
rs110008873	10	67276588	A	G	743	5.508	3.63E-08
BovineHD1100001194	11	3312404	T	C	280	3.633	0.00028
rs134083327	12	10040464	G	A	2406	6.764	1.34E-11
rs134046542	12	54289535	G	A	2406	6.728	1.72E-11
rs110121749	12	46243629	A	C	2406	6.627	3.43E-11
13:81673732	13	81673732	C	T	150	5.483	4.17E-08
ARS-BFGL-NGS-109467	13	28331553	A	G	280	3.76	0.00017
BovineHD1300013919	13	47642260	A	C	280	3.624	0.00029
BovineHD1400017260	14	62204044	T	C	280	4.07	4.70E-05
rs133977719	14	28398738	T	C	802	3.737	0.000186
BovineHD1500006429	15	24508531	A	C	150	5.598	2.17E-08
15:25797132	15	25797132	A	G	150	5.451	5.00E-08
rs42691659	16	63144583	C	T	2406	6.445	1.15E-10
rs110766243	17	32120758	G	A	2406	6.77	1.29E-11
rs134209520	17	48682081	T	C	743	5.264	1.41E-07
rs109484372	17	44884744	A	G	743	5.229	1.70E-07
BovineHD1700000175	17	713923	A	G	150	5.223	1.76E-07
Hapmap48751-BTA-41232	17	58016927	A	G	280	3.775	0.00016
BovineHD1700016427	17	58018395	A	C	280	3.673	0.00024
BovineHD1800009917	18	32762956	A	G	150	4.77	1.84E-06
rs134127572	19	36424186	A	G	2406	6.958	3.45E-12
19:24494923	19	24494923	G	C	150	5.851	4.89E-09
rs110820800	19	59382179	C	A	802	3.748	0.000178
rs109366906	20	18472746	T	C	2406	6.558	5.46E-11
ARS-BFGL-NGS-87102	20	49859323	A	G	150	5.404	6.52E-08
rs110957960	20	20494060	A	G	1604	5.26	1.44E-07
rs136158794	23	39522823	C	T	2406	6.998	2.60E-12
rs133044483	23	39502283	C	A	2406	6.998	2.60E-12
rs110389869	24	54044457	A	G	2406	6.748	1.50E-11
rs135137105	24	22424171	T	C	2406	6.449	1.13E-10
28:21771233	28	21771233	C	T	150	5.478	4.30E-08
BTB00987935	28	35294673	C	T	287	3.658	0.000254
ARS-BFGL-NGS-12759	28	15739975	A	G	280	3.624	0.00029

CHR: Chromosome.

* Weight indicates the total sample size contributing to the meta-analysis for each SNP, corresponding to the sample-size–weighted Z-score approach implemented in the METAL program.

Additionally, Z-score values, derived from the transformation of combined P-values, represent both the magnitude and directionality of the aggregated SNP effects across the analyzed studies.

### Functional annotation, gene ontology, and network analysis

**Fig 3:**
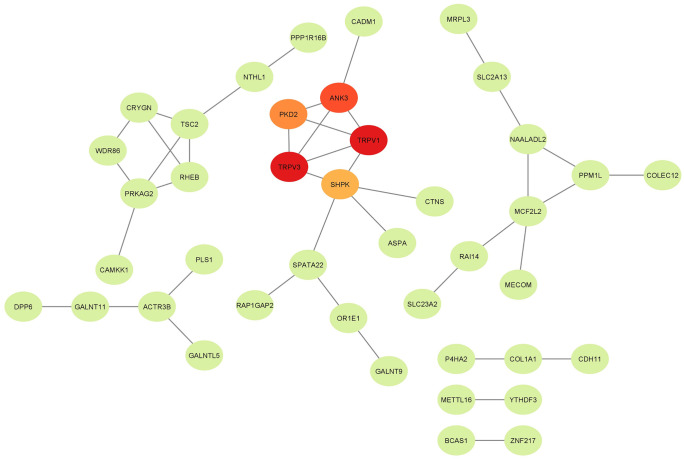
PPI network of candidate genes and hub genes. The network illustrates the interactions among candidate genes, with node color intensity indicating their degree of connectivity. The top five hub genes: *TRPV3*, *TRPV1*, *ANK3*, *PKD2*, and *SHPK,* are shown in red and orange, representing higher centrality within the network.

The meta-analysis resulted in the identification of 113 genes associated with significant SNPs. To explore the functional roles of these genes, pathway annotations were retrieved from the DAVID database and categorizedinto three major GO domains: BP, CC, and MF. The results indicated that the candidate genes are involved in a wide range of biological functions and pathways. Detailed functional annotations for each gene, including gene ID, gene name, and associated GO terms, are provided in S3 Table, which provides additional information on the different biological functions of individual genes, especially those are not present in any gene interaction networks or functional clustering analyses. In this study, a topological analysis of significant nodes in an interactome network was conducted by CytoHubba plug-in, which combines topological analysis algorithms. Among the eleven topological analysis methods implemented in CytoHubba, MCC was found to be the most accurate in identifying essential proteins [48] and hence it was used to identify essential nodes. Utilizing the CytoHubba plugin in Cytoscape [49], five genes: *TRPV3*, *TRPV1*, *ANK3*, *PKD2*, and *SHPK* were identified as central hub genes within the interaction network (Fig 3).

**Fig 4:**
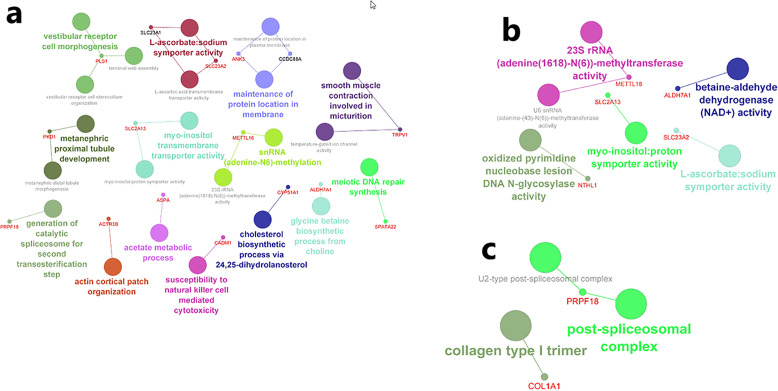
Functional enrichment analysis of candidate genes using ClueGO and CluePedia in Cytoscape. **(a)** Gene ontology enrichment for Biological Processes (BP); **(b)** Cellular Components (CC); **(c)** Molecular Functions (MF).

In general, gene expression networks are constructed in Cytoscape based on the degree of correlation or interaction among relevant genes [50]. The ClueGO and CluePedia plugins in Cytoscape were employed to identify and visualize the biological, molecular, and cellular functions of genes derived from the GWAS meta-analysis. The result provides an integrated visualization of three major GO categories (Fig 4a–4c).

Each node in the network represents a GO term, and the edges indicate functional relationships based on shared gene involvement and dependency. The candidate genes were found to be involved in a wide range of biological and molecular functions, including metabolic and enzymatic pathways to processes associated with morphogenesis, immune response, and cellular structure organization. This functional network does not only point out the different functions of these genes in various biological contexts but also highlights pathways which can be used as future targets of functional validation or mechanistic studies which can be applied in the trait of interest. Under BP term, various important pathways were identified. Among them, there are the acetate metabolic process, which entails the *ASPA* gene, and the cholesterol biosynthetic process via 24,25-dihydrolanosterol, regulated by *CYP51A1*, which underscores the gene’s role in cholesterol metabolism. Immune-related processes, such as susceptibility to natural killer cell-mediated cytotoxicity and cytoskeletal pathways like actin cortical patch organization, further illustrate the involvement of candidate genes in immune modulation and cellular architecture. In the MF category, the network was predominantly enriched for pathways related to enzymatic activity and membrane transport. For instance, snRNA (adenine-N6)-methylation activity associated with *METTL16* and betaine-aldehyde dehydrogenase (NAD+) activity linked to *ALDH7A1* highlight functional roles in RNA methylation and osmolyte metabolism, respectively. Within the CC category, genes were associated with structures central to RNA processing and extracellular matrix organization. The post-spliceosomal complex, represented by *PRPF18*, is essential for post-transcriptional RNA splicing, while the collagen type I trimer cluster, including *COL1A1*, reflects the contribution of candidate genes to extracellular matrix integrity and tissue stability.

**Fig 5:**
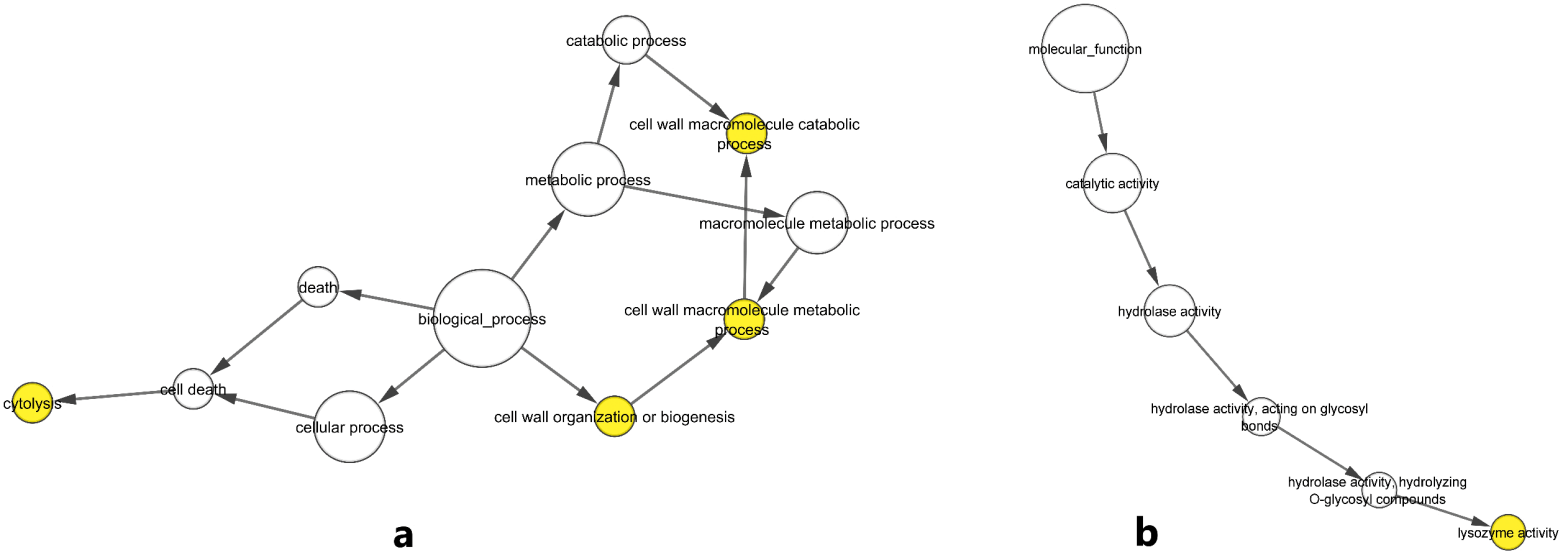
GO analysis of candidate genes using BiNGO. **(a)** Gene ontology enrichment for biological processes; **(b)** Gene ontology enrichment for molecular functions. Node color intensity reflects the significance of GO term enrichment (adjusted P-value), with darker colors indicating more significant enrichment.

To elucidate the functional roles of the identified genes, an enrichment analysis using the BiNGO plugin of Cytoscape was performed, with regard to biological processes and molecular functions. The biological process analysis revealed significant enrichment of pathways associated with cytolysis, cell wall organization or biogenesis, cell wall macromolecule metabolism, and catabolism (Fig 5a). These findings indicate that the selected genes are actively involved in essential pathways related to the construction, degradation, and remodeling of cell walls. Also, their participation in cytolytic processes indicates their potential roles in cell death mechanisms that result in the disruption of cellular integrity and release of intracellular contents. All of these pathways may be critical for regulating diverse cellular activities, including stress responses, immune reactions, and tissue regeneration processes. In terms of molecular functions, our analysis identified significant enrichment in enzymatic activities, notably lysozyme activity (Fig 5b).

Lysozymes are enzymes that are part of the regulation of microbes and cellular protection against pathogenic invasion by catalyzing the hydrolysis of glycosidic bonds in the polysaccharide frameworks especially bacterial cell walls [51]. The identified activity of lysozyme is hierarchically organized in terms of cascading of molecular functions that include catalytic activity, hydrolase activity, hydrolase activity targeting glycosyl bonds, and eventually, hydrolase activity targeting O glycosyl compounds. These findings underscore the targeted catalytic and hydrolytic activity of the candidate genes in the various biological and cellular pathways.

**Fig 6:**
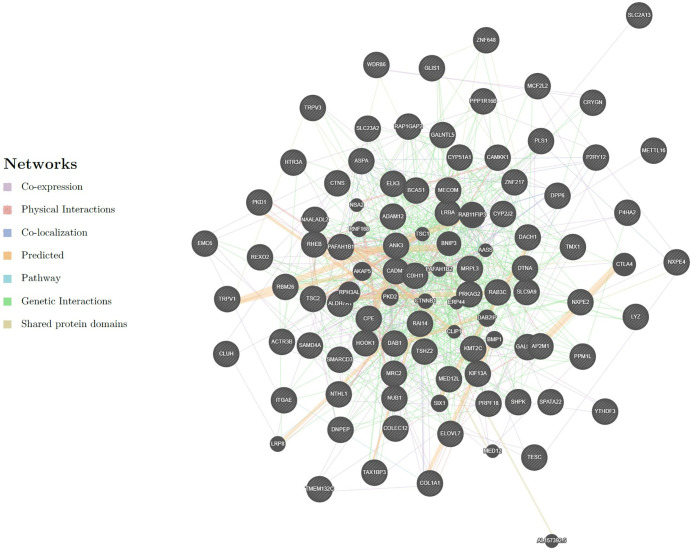
Gene interaction network of candidate genes generated by GeneMANIA. The network integrates co-expression, physical interactions, pathways, co-localization, and other functional links among candidate genes. Edge colors indicate interaction types as defined in the legend.

In the present study, the GeneMANIA was utilized to investigate the interactions among candidate genes at both the gene and protein levels. In this analysis, the candidate genes that were identified by genome-wide association meta-analysis were evaluated within the context of an integrated interaction network (Fig 6).

The majority of interactions observed within the constructed network were classified as physical interactions, comprising 70.9% of all connections. These interactions reflect direct binding events between proteins encoded by the candidate genes, suggesting their participation in shared protein complexes or molecular pathways at the protein level. The high prevalence of such interactions implies potential synergistic functions among these gene products within the cellular environment. Co-expression interactions represented 16.01% of the network. These associations are inferred from correlated gene expression patterns across various biological contexts and conditions. High levels of co-expression typically indicate coordinated gene regulation in response to shared environmental stimuli or involvement in common biological pathways. Predicted interactions accounted for 4.96% of total connections. Although these interactions have not yet been experimentally validated, they are derived from computational modeling and suggest potential functional relationships. Their presence, alongside confirmed physical and co-expression interactions, may point to genes with previously unrecognized but functionally analogous roles, highlighting targets for future experimental validation. Other categories included co-localization (3.22%), genetic interactions (2.63%), pathway-based connections (1.74%), and shared protein domains (0.55%). Detailed information on GeneMANIA interactions, including co-expression gene pairs and associated network data, is provided in S4 Table. A comprehensive functional analysis of the identified genes was performed by using the DAVID web tool. The results were systematically grouped into four primary categories: BP, CC, MF, and KEGG signaling pathways. The detailed outputs are presented in Tables 4 and 5.

**Table 4:** Gene ontology analysis performed by the DAVID web tool.

BP	Term	P-value	Genes	Fold enrichment
GO:0042742 ~ defense response to bacterium	0.027767	COLEC12, LYZ2, SLC9A9, LYSB	6.044201
GO:0047496 ~ vesicle transport along microtubule	0.063015	KIF13A, PAFAH1B1	30.39878
GO:0046329 ~ negative regulation of JNK cascade	0.063015	MECOM, PAFAH1B1	30.39878
GO:0051056 ~ regulation of small GTPase-mediated signal transduction	0.084307	RAP1GAP2, TSC2	22.46866
GO:0098703 ~ calcium ion import across plasma membrane	0.084307	TRPV3, TRPV1	22.46866
GO:0043488 ~ regulation of mRNA stability	0.087809	YTHDF3, SAMD4A	21.53247
**MF**	GO:0005262 ~ calcium channel activity	0.023615	TRPV3, TRPV1, PKD1	12.47166
	GO:0008013 ~ beta-catenin binding	0.044274	CDH11, TAX1 BP3, MED12L	8.850859
	GO:0005515 ~ protein binding	0.051124	PPP1R16B, CLUH, SAMD4A, TRPV3, WDR86, ANK3, TRPV1, PKD1, COL1A1, NUB1, MECOM, P4HA2, TAX1 BP3, RAI14	1.736561
	GO:0005261 ~ monoatomic cation channel activity	0.06957	TRPV3, PKD1	27.43766
	GO:0004653 ~ polypeptide N-acetylgalactosaminyltransferase activity	0.07292	GALNT11, GALNT9	26.13111
	GO:0046872 ~ metal ion binding	0.079312	COLEC12, RBM26, PPM1L, KMT2C, TSHZ2, GLIS1, COL1A1, P2RX5, RHEB, NTHL1, ADAM12, ZNF217, ASPA	1.668333
	GO:0005230 ~ extracellular ligand-gated monoatomic ion channel activity	0.079585	HTR3A, TRPV1	23.85884
	GO:0004857 ~ enzyme inhibitor activity	0.079585	PPP1R16B, TMX1	23.85884
	GO:0003796 ~ lysozyme activity	0.082899	LYZ2, LYSB	22.86472
	GO:0001227 ~ DNA-binding transcription repressor activity, RNA polymerase II-specific	0.087497	DACH1, ZNF217, GLIS1, ELK3	3.7715
	GO:0051219 ~ phosphoprotein binding	0.089493	TRPV1, PAFAH1B1	21.10589
**CC**	GO:0012505 ~ endomembrane system	0.002392	RHEB, TMX1, SLC2A13, GALNTL5, CTNS, PKD1	6.390518
	GO:0005737 ~ cytoplasm	0.026704	SLC23A2, PRKAG2, CAMKK1, DNPEP, KIF13A, METTL16, MCF2L2, PLS1, RAP1GAP2, PPP1R16B, CLUH, DTNA, YTHDF3, TESC, SAMD4A, TSC2, ANK3, REXO2, CYP2J30, COL1A1, DAB1, RHEB, HOOK1, CDH11, TAX1 BP3, ASPA	1.495213
	GO:0016323 ~ basolateral plasma membrane	0.032552	SLC23A2, TAX1 BP3, ANK3, PKD1	5.687561
	GO:0016607 ~ nuclear speck	0.038041	PPP1R16B, PRPF18, DACH1, MECOM, ZNF217	3.906292
	GO:0016020 ~ membrane	0.048595	P2RY12, COLEC12, RAB3C, SLC23A2, TMEM132C, DTNA, TMEM178B, CADM1, PPM1L, LRBA, CYP51A1, NXPE2, CTNS, ANK3, NAALADL2, MRC2, TMEM233, DPP6, RHEB, ADAM12, GALNTL5, CPE, EMC6, GALNT9	1.448753
	GO:0005829 ~ cytosol	0.055484	RAP1GAP2, PPM1L, LRBA, KMT2C, TESC, SAMD4A, PRKAG2, NUB1, DNPEP, DACH1, RHEB, P4HA2, SHPK, ALDH7A1, ASPA, RAI14, PAFAH1B1, PLS1	1.564427
	GO:0005794 ~ Golgi apparatus	0.064664	GALNT11, DACH1, GALNTL5, CPE, TSC2, PKD1, GALNT9	2.436532
	GO:0001650 ~ fibrillar center	0.074174	SAMD4A, TAX1 BP3, RAI14	6.613443
	GO:0032420 ~ stereocilium	0.083355	PAFAH1B1, PLS1	22.75024
	GO:0000118 ~ histone deacetylase complex	0.099183	MECOM, ZNF217	18.95854

BP: Biological Processes; CC: Cellular Components; MF: Molecular Functions.

**Table 5:** Enriched functional pathways.

Term	P-value	Genes	Fold enrichment
bta00512:Mucin type O-glycan biosynthesis	0.010210213	GALNT11, GALNTL5, GALNT9	19.0744186
bta00514:Other types of O-glycan biosynthesis	0.017229165	GALNT11, GALNTL5, GALNT9	14.51314459
bta00310:Lysine degradation	0.03379633	MECOM, KMT2C, ALDH7A1	10.11522199
bta01100:Metabolic pathways	0.046363087	GALNT11, KMT2C, CYP51A1, ELOVL7, CYP2J30, MECOM, P4HA2, SHPK, GALNTL5, ALDH7A1, ASPA, GALNT9, PAFAH1B1	1.756498779
bta04211:Longevity regulating pathway	0.05909808	RHEB, PRKAG2, TSC2	7.417829457
bta04750:Inflammatory mediator regulation of TRP channels	0.083790118	TRPV3, TRPV1, CYP2J30	6.069133192
bta00340:Histidine metabolism	0.092335928	ALDH7A1, ASPA	20.23044397

On the BP category, the candidate genes were highly enriched in a wide variety of specialized pathways, most notably the defense response to bacteria, a process that also involves *COLEC12* and *LYZ2*, *SLC9A9* and *LYSB*, indicating a key role in innate immunity.

Other enriched pathways included vesicular transport along microtubules, negative regulation of the JNK cascade, regulation of small GTPase-mediated signaling, calcium ion import from the plasma membrane, and regulation of mRNA stability. The candidate genes in the CC category were majorly foundin structures such as the endomembrane system, cytoplasm, basolateral plasma membrane, nuclear speck, cell membrane, and Golgi apparatus. Notably, genes such as *RHEB, TMX1, SLC2A13, GALNTL5*, *CTNS*, and *PKD1* were centrally involved in the endomembrane system. More specialized components, including the fibrillar center, stereocilium, and histone deacetylase complex, were also highlighted, suggesting roles in nuclear activity and the regulation of gene expression. At the MF category, enriched terms included calcium channel activity, involving *TRPV3, TRPV1*, and *PKD1*, as well as beta-catenin binding, protein binding, and more specialized molecular activities such as single-atom ion channel activity, galactosaminyltransferase activity, metal ion binding, enzyme inhibition, lysozyme activity, and phosphorylated protein binding. These results point to the broad involvement of the gene set in ion transport, epigenetic regulation, RNA processing, and intracellular signaling, functions that may underpin tissue-specific physiological roles. KEGG pathway analysis further revealed significant enrichment in mucin-type O-glycan biosynthesis, other types of O-glycan biosynthesis, lysine degradation, metabolic pathways, and signaling routes such as the longevity regulating pathway, regulation of TRP channels by inflammatory mediators, histidine metabolism, and thyroid hormone signaling. Among these, the mucin type O-glycan biosynthesis pathway ranked highest in priority, with the lowest p-value (0.0102) and a high enrichment score (19.07). *GALNT11, GALNTL5,* and *GALNT9* were involved in a variety of overlapping pathways indicating a central role in glycoprotein synthesis and intracellular interaction.

### Meta-analysis of genomic and pedigree-based heritability estimates

The effect sizes and heterogeneity statistics for genomic and pedigree-based heritability estimates of methane-related traits in cattle obtained from the random-effects meta-analysis model are presented in Tables 6 and 7, separated by the approach used (genomic and pedigree).

**Table 6:** Effect size and heterogeneity of the genomic heritability estimates for methane-related traits based on the random-effects meta-analysis model.

Trait	Study	N	h^2^	SE	95% CI	P-value	Q	P-value	I^2^
METP	14	19	0.296	0.035	0.227-0.366	0.000	164.282	0.000	89.043
METC	5	5	0.124	0.009	0.107-0.141	0.000	2.077	0.000	0.000

METP: methane production; METC: methane concentration; N: Number of literature estimates; SE: Standard error; 95% CI: 95% confidence interval.

**Table 7:** Effect size and heterogeneity of the pedigree-based heritability estimates for methane-related traits based on the random-effects meta-analysis model.

Trait	Study	N	h^2^	SE	95% CI	P-value	Q	P-value	I^2^
METP	28	38	0.303	0.020	0.264-0.342	0.000	705.149	0.000	94.753
METY	12	18	0.293	0.030	0.234-0.353	0.000	159.702	0.000	89.355
METINT	11	14	0.257	0.038	0.183-0.330	0.000	243.987	0.000	94.672
METC	7	12	0.207	0.022	0.164-0.250	0.000	58.583	0.000	81.223
RMET	4	10	0.167	0.014	0.138-0.195	0.000	4.262	0.000	0.000

METP: methane production; METY: methane yield; METINT: methane intensity; METC: methane concentration; RMET: residual methane; N: Number of literature estimates; SE: Standard error; 95% CI: 95% confidence interval.

In the genomic analyses, METP showed the highest heritability estimate (0.296), indicating a moderate to high genetic contribution to phenotypic variance. On the other hand, the genomic estimate of METC was significantly lower (0.124). There was significant heterogeneity between the studies of genomic METP, whereas METC showed very low heterogeneity.

The most heritable trait according to pedigree-based heritability estimates was METP (0.299). Methane yield (METY) also presented a similarly moderate heritability estimate (0.293). Moderate heritability estimates were observed for methane intensity (METINT; 0.257) and methane concentration (METC; 0.193). The lowest heritability estimate among the evaluated traits was recorded for residual methane (RMET; 0.167).

The pedigree-based estimates also had a significant heterogeneity. There was the highest heterogeneity in METP (P < 0.001). Other traits, excluding RMET, displayed substantial heterogeneity.

**Fig 7:**
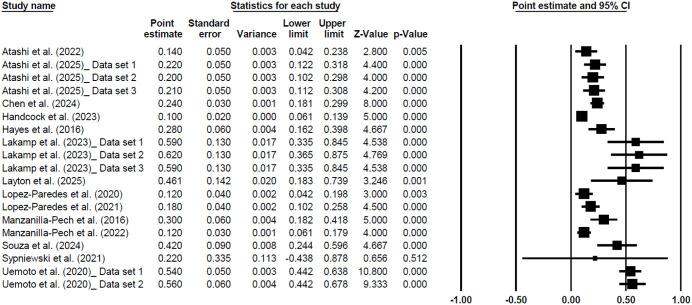
The forest plot of individual studies and the overall outcome for genomic heritability estimates of Methane Production. The mean effect size, calculated according to a random effects model, is indicated by the diamond at the bottom of each plot. The size of the squares illustrates the weight of each study relative to the mean effect size. Smaller squares represent less weight. The horizontal bars represent the 95% CI for the study.

**Fig 8:**
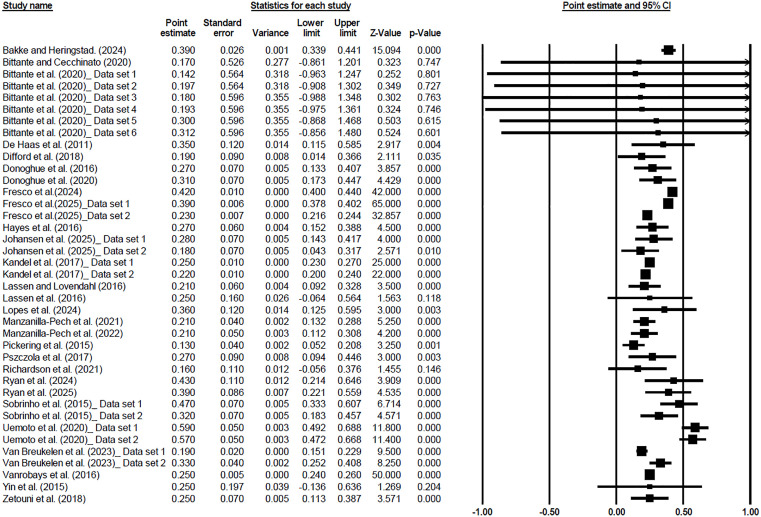
The forest plot of individual studies and the overall outcome for pedigree-based heritability estimates of Methane Production. Details are provided in Fig.7.

The forest plots of individual studies and the overall genomic and pedigree-based heritability estimates for METP are shown in Figs 7 and 8, respectively. The heritability estimates for other traits are presented in S1–S5 Figs.

To determine the possible publication bias of the meta-analytic heritability of the traits associated with methane, the regression test of Egger was used in all the analyzed traits, and the results are described in Table 8.

**Table 8:** Summary of publication bias diagnostics and funnel plot asymmetry correction using the trim-and-fill method for traits with no detected heterogeneity. The table presents results of statistical tests for publication bias and adjusted mean heritability estimates following the imputation of potentially missing studies.

Trait^*^	P-value of Egger’s Test	Trim and Fill		
		Missing	Mean	95% CI
METP-G	0.00166	2	0.272	0.204-0.340
METC-G	0.21440	2	0.120	0.103-0.136
METP-P	0.97772	0	0.303	0.264-0.342
METY-P	0.26428	0	0.293	0.234-0.353
METINT-P	0.46266	0	0.257	0.183-0.330
METC-P	0.76476	0	0.207	0.164-0.250
RMET-P	0.00081	4	0.154	0.129-0.180

*For traits, see Table 7. G: Genomic heritability; P: Pedigree-based heritability.

**Fig 9:**
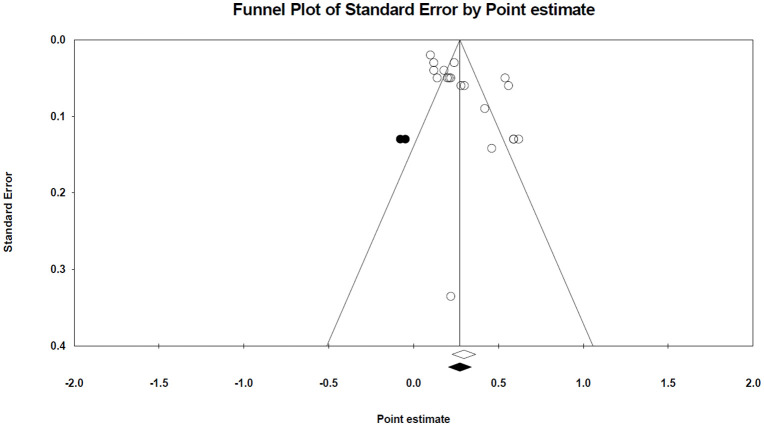
The funnel plot of the genomic heritability estimate for METP. Solid dots indicate the potentially missing studies identified through the trim-and-fill method. Open diamonds represent the pooled estimate and 95% confidence interval (CI) based solely on the published studies, whereas solid diamonds reflect the adjusted pooled estimate and CI after accounting for the imputed studies.

In the case of genomic METP, a statistically significant publication bias was found by Egger’s test. Therefore, Duval and Tweedie’s trim-and-fill method revealed two studies that might be missing on the left side of the funnel plot. The inclusion of these hypothetical studies produced the adjusted estimate of heritability of 0.273, which suggests that the original estimate may be inflated by publication bias. The funnel plot illustrating this analysis is presented in Fig 9.

Genomic METC did not show any significant publication biases (P > 0.10). Although the trim-and-fill method indicated the existence of two missing studies. After inclusion of these hypothetical studies, the adjusted heritability estimate was 0.114. The funnel plot for this trait is provided in S6 Fig.

**Fig 10:**
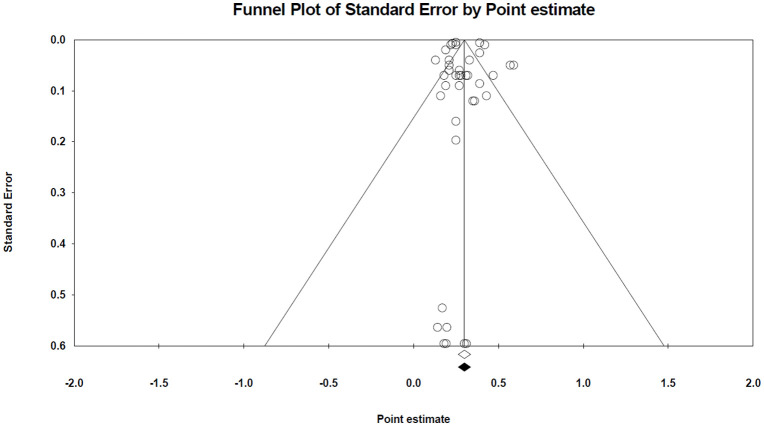
The funnel plot of the pedigree-based heritability estimate for METP. Details are provided in Fig 9.

In the pedigree-based analyses, Egger’s test didn’t show any significant publication bias for METP, METY, METINT, and METC (P > 0.10). Based on this, the trim-and-fill method did not identify any missing studies, and the adjusted heritability were consistent with the initial values. However, for RMET, significant publication bias was detected (P = 0.00041), with four missing studies estimated on the left side of the funnel plot. The adjusted heritability for RMET decreased to 0.154, indicating a meaningful reduction from the original estimate. Fig 10 shows the funnel plot of pedigree-based METP and similar plots of other traits are shown in S7–S10 Figs.

## Discussion

The investigation of methane-related traits across different cattle breeds represents a fundamental component of genomic strategies aimed atmitigating greenhouse gas emissions in the livestock production industry [3,52]. Genetic selection for reducing methane production is considered a sustainable approach because the realized genetic gainsare cumulatively transmitted to subsequent generations, thereby providing long-term environmental benefits [53,54]. In this context, the present research, employed a rigorous and comprehensive meta-analysis of GWAS to integrate heterogeneous evidence on methane traits across multiple breeds and experimental designs. Through systematic screening and inclusion of eligible studies, a consolidated dataset encompassing 4,336 animals was compiled, substantially increasing the statistical power compared with individual studies.

The Meta-analytical GWAS identified 173 SNPs associated with methane-related traits, of which 74 remained statistically significant after Bonferroni correction (P < 2.90 × 10^−4^). These significant SNPs were distributed across various chromosomes, underscoring the polygenic nature underlying methane production traits in cattle [20]. Further investigation of adjacent genes and descriptive studies showed that no common significant SNPs were found across studies, which was probably due to heterogeneity in study designs, breed composition, and statistical methodologies. The meta-analysis methodology used in the study helped combine and amplify statistical power of independent studies, thus, providing a wider range of expected genomic areas of methane traits. In addition to the discovery of the markers, the research provides a synthesis of the estimates of the genetic parameters, which clarifies the heritability of methane traits, using the evidence of genomic and pedigree [23,29,30]. When an extra quantitative meta-analysis based on the CMA framework was included, the estimates of heritabilities and heterogeneity parameters were refined to show a consistent picture of moderate genetic determination of production-level traits. These results align with previous reports in Holstein and Danish Holstein populations, where heritability estimates for methane production (0.16–0.49) confirm the feasibility of achieving genetic progress through selection [52,53].

Furthermore, the convergence between methane traits and correlated indicators such as feed efficiency underscores the practical breeding relevance of these results. Traits with positive genetic correlations with methane production can be efficiently integrated into selection indices, and thus, goals will be reached in both environmental sustainability and efficiency of production [19,54,55]. Taken together, this meta-analysis would not only summarize the existing evidence of the heterogeneous studies, but also point out the new possibilities of genomic optimization of the methane traits, which would provide a statistically reinforced and biologically oherent framework for sustainable livestock breeding.

The present dual analytical approach combining meta-GWAS with the independent meta-analysis of heritability estimates provides a full framework of the evaluation of both the genomic signals and the quantitative genetic variation of methane emission traits in cattle. This method does not determine the entire genetic architecture, but rather defines the recurrent patterns found across studies and estimates the strength of additive genetic control, which can be used as a biologically and statistically supported basis of future validation. The findings underscore the fact that the methane characteristics, though polygenic and environmentally adjusted, are moderately heritable and hence they should be considered as the possible targets of selection in breeding programs. Notably, this synthesis fills the gap between molecular level understanding of SNP and pathway analysis and population level demonstrations of heritable variation which contributes to the better understanding of genomic data into practical breeding. The study thus leads to a more integrative view of the possibility of host genetics to affect methane phenotyping so that they may serve as the basis of evidence in the design of future genomic prediction and selection systems. The production of methane-associated characteristics should be targeted to achieve global climate and sustainability outcomes and preserve the efficiency of production. These traits are economically feasible and genetic gains can be increased during consecutive generations. In light of global initiatives aiming to reduce methane to lower the amount of methane released into the atmosphere by the year 2050, the use of genetic enhancement of cattle with special attention to methane characteristics is a factual and realistic avenue that can be used to meet climate goals. However, further merging of functional genomics and metagenomic data will be necessary in order to transform these statistical linkages into informative and practical breeding tools [53,56].

To further investigate the biological context of the identified genes from SNPs, post-GWAS enrichment analyses including GO terms and KEGG pathways were performed. *TRPV1 and TRPV3* were also discovered as hub genes in the protein-protein interaction network and were enriched in significant biological pathways, such as calcium ion import across the plasma membrane (GO:0098703), calcium channel activity (GO:0005262), and the regulation of inflammatory mediators by TRP channels (bta04750). The convergent pattern of these overlapping enrichments in independent analyses supports the idea that the epithelial ion-transport processes are the emerging biological signal in representations of methane-related traits. These genes have been identified earlier to be related with SNP predictive of methane emission in GWAS on Holstein cattle [19].

This process can aid the metabolism of nitrogen and can possibly affect the uptake of ammonia which has been indirectly associated with the emission of nitrous oxide into the environment. Although the present meta-analysis does not provide direct functional evidence linking *TRPV3* to methane emission, its enrichment in ion transport and signaling pathways, together with prior expression studies in the rumen, indicates a putative role in modulating epithelial bioelectrical properties that could influence rumen fermentation dynamics and microbial composition. Hence, *TRPV3* may act as a contextually relevant candidate gene having an indirect effect on the emission of methanethrough nitrogen and microbial homeostasis, though this remains to be experimentally validated [57,58].

*TRPV1* was enriched in the same GO terms and the same KEGG pathway (e.g., GO:0098703, GO:0005515 and bta4750) as *TRPV3*, but its expression has a more peripheral distribution. It is primarily located in systemic calcium signaling and it is not specifically expressed in the rumen epithelium*.* Therefore, its identification in GWAS may reflect broader physiological correlations or linkage disequilibrium with functionally relevant variants rather than direct causality within the rumen.

In addition to the *TRP* family, several genes were enriched in metabolic and structural pathways, illustrating the multifactorial nature of methane-related traits. *SHPK*, encoding sedoheptulokinase, catalyzes the conversion of sedoheptulose to sedoheptulose-7-phosphate in the non-oxidative phase of the pentose phosphate pathway (PPP). This pathway plays an essential role in redox balance and NADPH production, supporting lipid and nucleotide biosynthesis [59]. *SHPK* was localized to the cytosol (GO:0005829) and enriched in metabolic pathways (bta01100), suggesting its function in maintaining cellular energy and redox homeostasis. Although not directly implicated in methane metabolism, *SHPK’s* regulatory function in carbon flux and oxidative balance may indirectly affect feed efficiency, a trait inversely related to methane yield [60].

Also, GO annotations indicate that *ANK3* is involved in several key cellular functions: protein binding (GO:0005515), cytoplasmic localization (GO:0005737), association with the membrane (GO:0016020), and specific targeting to the basolateral plasma membrane in epithelial cells (GO:0016323). Its expression is especially high in excitable and polarized tissues such as brain, muscle, and cardiac cells [61,62], with multiple isoforms generated via alternative splicing [63]. *ANK3* encodes ankyrin-G, a scaffolding protein that anchors membrane proteins to the spectrin–actin cytoskeleton across various cell types [64–66] and consists of multiple ankyrin repeats, a spectrin-binding region, and a regulatory C-terminal domain [67]. While *ANK3* contributes to cell polarity and membrane organization, there is currently no experimental evidence linking it directly to methane metabolism. Its identification across multiple GO categories likely reflects a structural or regulatory cellular role rather than trait-specific functionality [68–70].

Among the genes located near significant SNPs identified in the current meta-analysis (rs43508672, rs43508661, rs43508667, rs29023390, rs43508669), *P4HA2* was notable for its in protein binding (GO:0005515), cytosolic localization (GO:0005829), and metabolic pathways (bta01100). *P4HA2* encodes prolyl 4-hydroxylase subunit alpha-2, an enzyme involved in collagen biosynthesis and extracellular matrix (ECM) organization. Its co-occurrence with ECM-related genes such as *COL1A1* supports the hypothesis that structural remodeling processes may influence tissue metabolism and, indirectly, methane-related physiology [16]. *P4HA2* has also been implicated in metabolic adaptation under hypoxia. It was identified as a hub gene in Tibetan sheep and goats, where it appears to regulate meat quality, skeletal muscle development, and high-altitude hypoxia response [71,72]. In Tibetan sheep, it resides within a breed-specific ROH island associated with hypoxic adaptation [72]. Its expression is also regulated by the p53 and HIF1α signaling axes under hypoxic stress, interacting with pathways such as mTOR and VEGF signaling [73]. Moreover, *P4HA2* has been reported to participate in PI3K/AKT signaling and epithelial–mesenchymal transition (EMT) in non-ruminant models, indicating broader regulatory capacity in cellular proliferation and metabolism [74,75]. The potential implication of the role of *P4HA2* in energy metabolism and ECM remodeling is that it may have indirect effects on methane yield. The ECM influences cellular signaling, energy partitioning, and potentially immune responses, all of which may shape rumen physiology and microbial interactions. The co-localization of ECM-related genes with methane-associated SNPs further underscores their potential regulatory significance, warranting further functional validation in the context of host–microbiome–environment interactions.

Consistent with this ECM-related signal, *COL1A1* was enriched highly in protein binding (GO:0005515), metal ion binding (GO:0046872), cytoplasmic localization (GO:0005737), and collagen-related cluster of ClueGO/CluePedia results. Previous studies reported the downregulation of *COL1A1* in ruminal epithelial cells of high-gain cattle [76], which might be associated with energy metabolism and efficiency of nutrient-transport. *P4HA2* and *COL1A1* both were located within QTL regions associated with methane traits [16], indicating that ECM remodeling may serve as an intermediate layer, which combines cellular metabolism, tissue structure, and energy efficiency, which ultimately determine methane yield. *KIF13A* encodes a kinesin motor protein involved in microtubule-based vesicle transport, contributing to intracellular organization and membrane trafficking. KIF13A contributes to transfers in gastrointestinal epithelia, i.e., rumen where nutrient transport and metabolic regulation is vital in ensuring a stable microbial environment in ruminants [77,78]. Previous transcriptomic analyses identified *KIF13A* as differentially expressed between low- and high-residual feed intake cattle [79], supporting its potential involvement in energy efficiency pathways. Such mechanisms could indirectly affect methane production through altered nutrient utilization and oxygen availability in the rumen environment [80,81].

The network metabolic regulation was further supported by the enrichment of the *ALDH7A1*, *CYP51A1*, and *SHPK* in metabolic pathways (bta01100), which includes degradation of amino-acids, lipid metabolism, and redox regulation. *ALDH7A1* is involved in the lysine degradation (bta00310) and histidine metabolism (bta00340), which makes it coupled with the feed efficiency and antioxidant defense [82–84]. Similarly, *CYP51A1*, which is associated with sterol/steroid production, is implicated in lipid metabolism and energy use, which are pathways that have been reported to be linked to rumen fermentation and methane variation [20,85]. Taken together these results provide evidence that biological mechanisms of methane characteristics includes ion transport and ECM remodelling, and also energy/redox metabolism, which points to the integrative role of host genomic regulation of methane emission phenotypes.

In general, the post-GWAS analyses confirmed the presence of biological terms that were justified by several enrichment analyses (GO, KEGG, and network), and a interpretation of the results in accordance with the existing data. The results do not publish causality to specific genes but represent functionally consistent networks and pathways that produce a biologically viable pattern of the situation in which cattle variation is linked to methane. These results supplement the quantitative data of heritability meta-analyses and define mechanistic hypotheses which can be validated in empirical studies of functionality and metagenomics in future.

Our meta-analysis distinguished between pedigree-based and genomic heritability estimates for various methane-related traits in cattle (Tables 6 and 7). The integration of both approaches enables a more comprehensive assessment of the genetic potential underlying methane phenotypes while accounting for methodological heterogeneity among studies.

In the present meta-analysis, METP exhibited a moderate heritability (0.296), while METC showed a lower estimate (0.124), suggesting that genetic improvement through selection may be more effective for production-level traits than for concentration-based measures. These findings are in line with previous studies that reported similar or slightly lower genomic heritabilities for methane emissions. For instance, Manzanilla-Pech et al. (2016) estimated heritability values of 0.30 for METP in Angus cattle and 0.23–0.42 for methane-related traits in Holstein populations [86]. Comparable estimates have also been reported in sheep, indicating a consistent genetic basis across ruminants [21,87]. More recent studies applying both direct and indirect phenotyping methods reported genomic heritability estimates in the range of 0.11 to 0.15 [88,89], though lower values may reflect differences in sensor technologies, recording environments, or data structures. These findings support the moderately heritable nature of methane traits. While phenotypic variability may be influenced by measurement approach and animal stage, the consistent presence of additive genetic variation across studies indicates that methane emission traits can be targeted in breeding programs, particularly when supported by accurate genomic evaluations and repeated records for improved reliability [89–92].

The pedigree-based heritability estimates obtained in this meta-analysis further underscore the genetic basis of methane-related traits in cattle. Among the evaluated traits, METP showed the highest heritability, followed closely by methane yield. Moderate heritability was observed for methane intensity, methane concentration, and residual methane, reflecting considerable additive genetic variance across trait definitions. These results are consistent with prior studies that have reported moderate heritabilities for methane traits in both dairy and beef cattle. For example, Kamalanathan et al. (2023) estimated heritabilities of 0.16, 0.27, and 0.21 for METP, METY, and METINT, respectively, in Holstein cattle under pedigree-based models [52]. Similarly, Ryan et al. (2025) observed heritability estimates ranging from 0.15 to 0.39, depending on trait definition and measurement approach [93]. Bittante et al. (2020) also highlighted moderate heritabilities for methane emissions predicted from milk fatty acid profiles, supporting the view that these traits are genetically tractable [94]. The heritability is theoretically non-negative; however, the confidence intervals for some individual studies in forest plots (Figs 7 and 8) were calculated using Wald-type normal approximations (estimate ± 1.96 × SE) within a random-effects meta-analysis framework, which does not impose boundary constraints. Consequently, for estimates with larger standard errors, the lower bound of the confidence interval may extend below zero due to sampling uncertainty. These negative values do not imply negative heritability but indicate that the true value may be close to zero.

The variation observed across studies is partly attributable to differences in phenotyping strategies, measurement technologies, and environmental conditions. Consistent with these observations, the present meta-analysis reinforces the hypothesis that methane emissions exhibit sufficient genetic variability to be included as selection objectives in breeding programs. Traits such as METP and METY, with moderate heritability, appear particularly promising for genetic improvement. Furthermore, established genetic correlations between methane traits and feed efficiency [95–97], suggest that selection for reduced methane emission may concurrently enhance resource-use efficiency. Integrating METP into breeding indices, alongside careful trait definition and measurement standardization, holds strong potential to support sustainable dairy and beef production systems with a minimal environmental footprint.

## Conclusions

The present study combined two complementary meta-analytical approaches to provide that integration of GWAS and heritability data enabled a comprehensive view of the genetic architecture underlying methane emission traits in cattle. Post-GWAS analyses identified several candidate genes enriched in biological processes that were mainly related to energy metabolism, epithelial ion transport and extra cellular matrix remodelling; these results highlight the multivariate and regulatory aspects of the phenotypes of methane. Taken together, the results advance a more refined understanding of the molecular mechanisms that may indirectly influence methane production, while acknowledging the need for functional validation to establish causality.

In a quantitative genetics perspective, the meta-analysis of heritability revealed consistent moderate genetic heritability, specifically on METP and METY. These findings confirm the fact that methane traits are sufficiently heritable to be targeted within selection criteria, while also defining methodological heterogeneity across studies. Importantly, the consistency of genomic and pedigree-based estimates reinforces the reliability of these parameters and their applicability in breeding programs. Overall, this study highlights the value of combining the evidence of functional genomics with the assessment of breeding in developing breeding strategies that can suppress methane emissions. The results indicate that genomic selection which focuses on traits associated with methane can be practiced and also potentially increase feed efficiency, which can be used to enhance environmentally friendly livestock production. To improve the biological basis of methane mitigation through genetic selection, future studies need to focus on refining phenotyping, expand multi-breed genomic data sets, and experimentally validate candidate genes of critical importance.

## Supporting information

S1 Checklist The PRISMA checklist used in this study. (DOCX)

S1 Table The characteristics of studies included in h^2^ meta- analysis. (DOCX)

S2 Table List of the identified genes related to significant SNPs. (DOCX)

S3 Table Detailed functional annotations for identified genes related to significant SNPs. (XLSX)

S4 Table Genemania Interactions. (DOCX)

S1 Fig The forest plot of individual studies and the overall outcome for the genomic heritability estimate of METC. The mean effect size, calculated according to a random effects model, is indicated by the diamond at the bottom of each plot. The size of the squares illustrates the weight of each study relative to the mean effect size. Smaller squares represent less weight. The horizontal bars represent the 95% CI for the study.
(PDF)

S2 Fig The forest plot of individual studies and the overall outcome for the pedigree-based heritability estimate of METC. Details are provided in S1 Fig.
(PDF)

S3 Fig The forest plot of individual studies and the overall outcome for the pedigree-based heritability estimate of METI. Details are provided in S1 Fig.
(PDF)

S4 Fig The forest plot of individual studies and the overall outcome for the pedigree-based heritability estimate of METY. Details are provided in S1 Fig.
(PDF)

S5 Fig The forest plot of individual studies and the overall outcome for the pedigree-based heritability estimate of RMET. Details are provided in S1 Fig.
(PDF)

S6 Fig The funnel plot of the genomic heritability estimate for METC. Solid dots represent the potentially missing studies that were found using the trim-and-fill method. When theoretically imputed studies are included in the meta-analysis, solid diamonds represent the mean values and CI, and open diamonds represent the mean values and confidence intervals for studies that are currently in the literature.
(PDF)

S7 Fig The funnel plot of the pedigree-based heritability estimate for METC. Details are provided in S6 Fig.
(PDF)

S8 Fig The funnel plot of the pedigree-based heritability estimate for METI. Details are provided in S6 Fig.
(PDF)

S9 Fig The funnel plot of the pedigree-based heritability estimate for METY. Details are provided in S6 Fig.
(PDF)

S10 Fig The funnel plot of the pedigree-based heritability estimate for RMET. Details are provided in S6 Fig.
(PDF)

## References

[1] Zhang L, Tian H, Shi H, Pan S, Chang J, Dangal SRS, et al. A 130-year global inventory of methane emissions from livestock: Trends, patterns, and drivers. Glob Chang Biol. 2022;28(17):5142–58. doi: 10.1111/gcb.16280 35642457

[2] Džermeikaitė K, Krištolaitytė J, Antanaitis R. Relationship between dairy cow health and intensity of greenhouse gas emissions. Animals (Basel). 2024;14(6):829. doi: 10.3390/ani14060829 38539927 PMC10967422

[3] Ghavi Hossein-Zadeh N. Estimates of the genetic contribution to methane emission in dairy cows: a meta-analysis. Sci Rep. 2022;12(1):12352. doi: 10.1038/s41598-022-16778-z 35853993 PMC9296463

[4] Ghavi Hossein-Zadeh N. A meta-analysis of the genetic contribution to greenhouse gas emission in sheep. J Anim Breed Genet. 2023;140(1):49–59. doi: 10.1111/jbg.12744 36263924

[5] Palangi V, Lackner M. Management of enteric methane emissions in ruminants using feed additives: a review. Animals (Basel). 2022;12(24):3452. doi: 10.3390/ani12243452 36552373 PMC9774182

[6] Palangi V, Taghizadeh A, Abachi S, Lackner M. Strategies to mitigate enteric methane emissions in ruminants: a review. Sustainability. 2022;14(20):13229. doi: 10.3390/su142013229

[7] Kader Esen V, Palangi V, Esen S. Genetic improvement and nutrigenomic management of ruminants to achieve enteric methane mitigation: a review. Methane. 2022;1(4):342–54. doi: 10.3390/methane1040025

[8] Križanac A-M, Reimer C, Heise J, Liu Z, Pryce JE, Bennewitz J, et al. Sequence-based GWAS in 180,000 German Holstein cattle reveals new candidate variants for milk production traits. Genet Sel Evol. 2025;57(1):3. doi: 10.1186/s12711-025-00951-9 39905301 PMC11796172

[9] Tang L, Swedlund B, Dupont S, Harland C, Costa Monteiro Moreira G, Durkin K, et al. GWAS reveals determinants of mobilization rate and dynamics of an active endogenous retrovirus of cattle. Nat Commun. 2024;15(1):2154. doi: 10.1038/s41467-024-46434-1 38461177 PMC10924933

[10] Ghavi Hossein-Zadeh N. An overview of recent technological developments in bovine genomics. Vet Anim Sci. 2024;25:100382. doi: 10.1016/j.vas.2024.100382 39166173 PMC11334705

[11] Kontou PI, Bagos PG. The goldmine of GWAS summary statistics: a systematic review of methods and tools. BioData Min. 2024;17(1):31. doi: 10.1186/s13040-024-00385-x 39238044 PMC11375927

[12] Korte A, Farlow A. The advantages and limitations of trait analysis with GWAS: a review. Plant Methods. 2013;9:29. doi: 10.1186/1746-4811-9-29 23876160 PMC3750305

[13] Ghavi Hossein-Zadeh N. A meta-analysis of genetic estimates for economically important traits in ducks. Vet Anim Sci. 2024;26:100405. doi: 10.1016/j.vas.2024.100405 39568627 PMC11576399

[14] Huang J, Lin X. Advances in animal disease resistance research: discoveries of genetic markers for disease resistance in cattle through GWAS. Biol Evid. 2024;14.

[15] Bang NN, Hayes BJ, Lyons RE, Randhawa IAS, Gaughan JB, Trach NX, et al. Genomic prediction and genome-wide association studies for productivity, conformation and heat tolerance traits in tropical smallholder dairy cows. J Anim Breed Genet. 2025;142(3):322–41. doi: 10.1111/jbg.12907 39462234

[16] Lakamp AD, Ahlberg CM, Allwardt K, Broocks A, Bruno K, Mcphillips L, et al. Variance component estimation and genome-wide association of predicted methane production in crossbred beef steers. J Anim Sci. 2023;101:skad179. doi: 10.1093/jas/skad179 37328159 PMC10284037

[17] Jang MJ, Lee SH, Kim JM. Genome-wide association studies: a powerful approach for identifying genomic variants for livestock breeding and disease management. In: Kim JM, Pathak RK, editors. Bioinformatics in veterinary science: Vetinformatics. Singapore: Springer Nature Singapore; 2025. p. 87–117.

[18] Souza LL, Dominguez-Castaño P, Gianvecchio SB, Sakamoto LS, Rodrigues GRD, Soares TL da S, et al. Heritability estimates and genome-wide association study of methane emission traits in Nellore cattle. J Anim Sci. 2024;102:skae182. doi: 10.1093/jas/skae182 38967061 PMC11282363

[19] Jalil Sarghale A, Moradi Shahrebabak M, Moradi Shahrebabak H, Nejati Javaremi A, Saatchi M, Khansefid M, et al. Genome-wide association studies for methane emission and ruminal volatile fatty acids using Holstein cattle sequence data. BMC Genet. 2020;21(1):129. doi: 10.1186/s12863-020-00953-0 33228565 PMC7684878

[20] Pszczola M, Strabel T, Mucha S, Sell-Kubiak E. Genome-wide association identifies methane production level relation to genetic control of digestive tract development in dairy cows. Sci Rep. 2018;8(1):15164. doi: 10.1038/s41598-018-33327-9 30310168 PMC6181922

[21] Pinares-Patiño CS, Hickey SM, Young EA, Dodds KG, MacLean S, Molano G, et al. Heritability estimates of methane emissions from sheep. Animal. 2013;7 Suppl 2(Suppl 2):316–21. doi: 10.1017/S1751731113000864 23739473 PMC3691003

[22] Richardson CM, Nguyen TTT, Abdelsayed M, Moate PJ, Williams SRO, Chud TCS, et al. Genetic parameters for methane emission traits in Australian dairy cows. J Dairy Sci. 2021;104(1):539–49. doi: 10.3168/jds.2020-18565 33131823

[23] Ghavi Hossein-Zadeh N. A meta-analysis of the genetic contribution estimates to major indicators for ketosis in dairy cows. Res Vet Sci. 2022;153:8–16. doi: 10.1016/j.rvsc.2022.10.008 36272179

[24] Borenstein M, Hedges LV, Higgins JPT, Rothstein HR. A basic introduction to fixed-effect and random-effects models for meta-analysis. Res Synth Methods. 2010;1(2):97–111. doi: 10.1002/jrsm.12 26061376

[25] Rojas de Oliveira H, Ventura HT, Costa EV, Pereira MA, Veroneze R, Duarte M de S, et al. Meta-analysis of genetic-parameter estimates for reproduction, growth and carcass traits in Nellore cattle by using a random-effects model. Anim Prod Sci. 2017;58(9):1575–83. doi: 10.1071/an16712

[26] Evans LM, Tahmasbi R, Vrieze SI, Abecasis GR, Das S, Gazal S, et al. Comparison of methods that use whole genome data to estimate the heritability and genetic architecture of complex traits. Nat Genet. 2018;50(5):737–45. doi: 10.1038/s41588-018-0108-x 29700474 PMC5934350

[27] Ghoreishifar M, Macleod IM, Chamberlain AJ, Liu Z, Lopdell TJ, Littlejohn MD, et al. An integrative approach to prioritize candidate causal genes for complex traits in cattle. PLoS Genet. 2025;21(5):e1011492. doi: 10.1371/journal.pgen.1011492 40446200 PMC12158001

[28] Hayes B, Goddard M. Genome-wide association and genomic selection in animal breeding. Genome. 2010;53(11):876–83. doi: 10.1139/G10-076 21076503

[29] Panagiotou OA, Willer CJ, Hirschhorn JN, Ioannidis JPA. The power of meta-analysis in genome-wide association studies. Annu Rev Genomics Hum Genet. 2013;14:441–65. doi: 10.1146/annurev-genom-091212-153520 23724904 PMC4040957

[30] Sahana G, Cai Z, Sanchez MP, Bouwman AC, Boichard D. Invited review: good practices in genome-wide association studies to identify candidate sequence variants in dairy cattle. J Dairy Sci. 2023;106(8):5218–41. doi: 10.3168/jds.2022-22694 37349208

[31] Zhou W, Kanai M, Wu K-HH, Rasheed H, Tsuo K, Hirbo JB, et al. Global Biobank Meta-analysis Initiative: Powering genetic discovery across human disease. Cell Genom. 2022;2(10):100192. doi: 10.1016/j.xgen.2022.100192 36777996 PMC9903716

[32] Page MJ, McKenzie JE, Bossuyt PM, Boutron I, Hoffmann TC, Mulrow CD, et al. The PRISMA 2020 statement: an updated guideline for reporting systematic reviews. BMJ. 2021;372:n71. doi: 10.1136/bmj.n71 33782057 PMC8005924

[33] Schardt C, Adams MB, Owens T, Keitz S, Fontelo P. Utilization of the PICO framework to improve searching PubMed for clinical questions. BMC Med Inform Decis Mak. 2007;7(1):16. 17573961 10.1186/1472-6947-7-16 PMC1904193

[34] Willer CJ, Li Y, Abecasis GR. METAL: fast and efficient meta-analysis of genomewide association scans. Bioinformatics. 2010;26(17):2190–1. doi: 10.1093/bioinformatics/btq340 20616382 PMC2922887

[35] Pósfai M, Barabási AL. Network science. Citeseer; 2016.

[36] Shannon P, Markiel A, Ozier O, Baliga NS, Wang JT, Ramage D, et al. Cytoscape: a software environment for integrated models of biomolecular interaction networks. Genome Res. 2003;13(11):2498–504. doi: 10.1101/gr.1239303 14597658 PMC403769

[37] Bindea G, Mlecnik B, Hackl H, Charoentong P, Tosolini M, Kirilovsky A, et al. ClueGO: a Cytoscape plug-in to decipher functionally grouped gene ontology and pathway annotation networks. Bioinformatics. 2009;25(8):1091–3. doi: 10.1093/bioinformatics/btp101 19237447 PMC2666812

[38] Bindea G, Galon J, Mlecnik B. CluePedia Cytoscape plugin: pathway insights using integrated experimental and in silico data. Bioinformatics. 2013;29(5):661–3. doi: 10.1093/bioinformatics/btt019 23325622 PMC3582273

[39] Maere S, Heymans K, Kuiper M. BiNGO: a Cytoscape plugin to assess overrepresentation of gene ontology categories in biological networks. Bioinformatics. 2005;21(16):3448–9. doi: 10.1093/bioinformatics/bti551 15972284

[40] Montojo J, Zuberi K, Rodriguez H, Kazi F, Wright G, Donaldson SL, et al. GeneMANIA Cytoscape plugin: fast gene function predictions on the desktop. Bioinformatics. 2010;26(22):2927–8. doi: 10.1093/bioinformatics/btq562 20926419 PMC2971582

[41] Brüggemann P, Rajguru K. Comprehensive meta-analysis (CMA) 3.0: a software review. J Mark Anal. 2022;10(4):425–9.

[42] Sutton AJ, Abrams KR, Jones DR, Sheldon TA, Song F. Methods for meta-analysis in medical research. Wiley Chichester; 2000.

[43] Duval S, Tweedie R. A Nonparametric “trim and fill” method of accounting for publication bias in meta-analysis. J Am Stat Assoc. 2000;95(449):89. doi: 10.2307/2669529

[44] Ioannidis JPA, Trikalinos TA. The appropriateness of asymmetry tests for publication bias in meta-analyses: a large survey. CMAJ. 2007;176(8):1091–6. doi: 10.1503/cmaj.060410 17420491 PMC1839799

[45] Manzanilla-Pech CIV, Difford GF, Sahana G, Romé H, Løvendahl P, Lassen J. Genome-wide association study for methane emission traits in Danish Holstein cattle. J Dairy Sci. 2022;105(2):1357–68. doi: 10.3168/jds.2021-20410 34799107

[46] Sarghale A, Moradi Shahrbabak H, Moradi Shahrebabak M, Nejati Javaremi A, Saatchi M, Miar Y. Genome wide association study to identify genome region associated with methane emission in cattle using 30K panel. Cell Mol Res. 2021;34(1):65–76.

[47] Calderón-Chagoya R, Hernandez-Medrano JHH, Ruiz-López FJJ, Garcia-Ruiz A, Vega-Murillo VEE, Montano-Bermudez M, et al. Genome-wide association studies for methane production in dairy cattle. Genes (Basel). 2019;10(12):995. doi: 10.3390/genes10120995 31810242 PMC6969927

[48] Chin C-H, Chen S-H, Wu H-H, Ho C-W, Ko M-T, Lin C-Y. cytoHubba: identifying hub objects and sub-networks from complex interactome. BMC Syst Biol. 2014;8 Suppl 4(Suppl 4):S11. doi: 10.1186/1752-0509-8-S4-S11 25521941 PMC4290687

[49] Chin C-H, Chen S-H, Wu H-H, Ho C-W, Ko M-T, Lin C-Y. cytoHubba: identifying hub objects and sub-networks from complex interactome. BMC Syst Biol. 2014;8 Suppl 4(Suppl 4):S11. doi: 10.1186/1752-0509-8-S4-S11 25521941 PMC4290687

[50] Hasin Y, Seldin M, Lusis A. Multi-omics approaches to disease. Genome Biol. 2017;18(1):83. doi: 10.1186/s13059-017-1215-1 28476144 PMC5418815

[51] Ferraboschi P, Ciceri S, Grisenti P. Applications of lysozyme, an innate immune defense factor, as an alternative antibiotic. Antibiotics (Basel). 2021;10(12):1534. doi: 10.3390/antibiotics10121534 34943746 PMC8698798

[52] Kamalanathan S, Houlahan K, Miglior F, Chud TCS, Seymour DJ, Hailemariam D, et al. Genetic analysis of methane emission traits in Holstein dairy cattle. Animals (Basel). 2023;13(8):1308. doi: 10.3390/ani13081308 37106871 PMC10135250

[53] Manzanilla-Pech CIV, Difford GF, Løvendahl P, Stephansen RB, Lassen J. Genetic (co-)variation of methane emissions, efficiency, and production traits in Danish Holstein cattle along and across lactations. J Dairy Sci. 2022;105(12):9799–809. doi: 10.3168/jds.2022-22121 36241442

[54] Worku D. Unraveling the genetic basis of methane emission in dairy cattle: a comprehensive exploration and breeding approach to lower methane emissions. Anim Biotechnol. 2024;35(1):2362677. doi: 10.1080/10495398.2024.2362677 38860914 PMC12674316

[55] Higgins MG, Fitzsimons C, McClure MC, McKenna C, Conroy S, Kenny DA, et al. GWAS and eQTL analysis identifies a SNP associated with both residual feed intake and GFRA2 expression in beef cattle. Sci Rep. 2018;8(1):14301. doi: 10.1038/s41598-018-32374-6 30250203 PMC6155370

[56] Eu A. European green deal: striving to be the first climate-neutral continent. European Union; 2019.

[57] Manneck D, Braun H-S, Schrapers KT, Stumpff F. TRPV3 and TRPV4 as candidate proteins for intestinal ammonium absorption. Acta Physiol (Oxf). 2021;233(1):e13694. doi: 10.1111/apha.13694 34031986

[58] Dhakal S, Lee Y. Transient receptor potential channels and metabolism. Mol Cells. 2019;42(8):569–78. doi: 10.14348/molcells.2019.0007 31446746 PMC6715338

[59] Stincone A, Prigione A, Cramer T, Wamelink MMC, Campbell K, Cheung E, et al. The return of metabolism: biochemistry and physiology of the pentose phosphate pathway. Biol Rev Camb Philos Soc. 2015;90(3):927–63. doi: 10.1111/brv.12140 25243985 PMC4470864

[60] Wallace RJ, Rooke JA, McKain N, Duthie C-A, Hyslop JJ, Ross DW, et al. The rumen microbial metagenome associated with high methane production in cattle. BMC Genomics. 2015;16:839. doi: 10.1186/s12864-015-2032-0 26494241 PMC4619255

[61] Tan Y, Meng W, Jiang Z, Li N, Zhang T, Zhang J, et al. A comprehensive analysis of the prognostic and immunological role of ANK3 in pan-cancer. Transl Cancer Res. 2024;13(2):1091–113. doi: 10.21037/tcr-23-2379 38482441 PMC10928612

[62] Rueckert EH, Barker D, Ruderfer D, Bergen SE, O’Dushlaine C, Luce CJ, et al. Cis-acting regulation of brain-specific ANK3 gene expression by a genetic variant associated with bipolar disorder. Mol Psychiatry. 2013;18(8):922–9. doi: 10.1038/mp.2012.104 22850628 PMC3856665

[63] Peters LL, John KM, Lu FM, Eicher EM, Higgins A, Yialamas M, et al. Ank3 (epithelial ankyrin), a widely distributed new member of the ankyrin gene family and the major ankyrin in kidney, is expressed in alternatively spliced forms, including forms that lack the repeat domain. J Cell Biol. 1995;130(2):313–30. doi: 10.1083/jcb.130.2.313 7615634 PMC2199924

[64] Smith KR, Penzes P. Ankyrins: roles in synaptic biology and pathology. Mol Cell Neurosci. 2018;91:131–9. doi: 10.1016/j.mcn.2018.04.010 29730177 PMC6128775

[65] Zhu X, Yu Y, Jiang Z, Otani Y, Fujitani M. Ankyrin-G and its binding partners in neurons: orchestrating the molecular structure of the axon initial segment. Biomolecules. 2025;15(6):901. doi: 10.3390/biom15060901 40563541 PMC12190637

[66] Bennett V, Healy J. Membrane domains based on ankyrin and spectrin associated with cell-cell interactions. Cold Spring Harb Perspect Biol. 2009;1(6):a003012. doi: 10.1101/cshperspect.a003012 20457566 PMC2882121

[67] Sharma N, Bham K, Senapati S. Human ankyrins and their contribution to disease biology: an update. J Biosci. 2020;45:146. doi: 10.1007/s12038-020-00117-3 33410423

[68] Hayashi A, Le Gal K, Södersten K, Vizlin-Hodzic D, Ågren H, Funa K. Calcium-dependent intracellular signal pathways in primary cultured adipocytes and ANK3 gene variation in patients with bipolar disorder and healthy controls. Mol Psychiatry. 2015;20(8):931–40. doi: 10.1038/mp.2014.104 25311363 PMC4759096

[69] Girard A, Dufort I, Douville G, Sirard M-A. Global gene expression in granulosa cells of growing, plateau and atretic dominant follicles in cattle. Reprod Biol Endocrinol. 2015;13:17. doi: 10.1186/s12958-015-0010-7 25879740 PMC4355352

[70] Gomez S, Morgans C. Interaction between band 3 and ankyrin begins in early compartments of the secretory pathway and is essential for band 3 processing. J Biol Chem. 1993;268(26):19593–7. doi: 10.1016/s0021-9258(19)36557-3 8366103

[71] Wen Y, Li S, Bao G, Wang J, Liu X, Hu J, et al. Comparative transcriptome analysis reveals the mechanism associated with dynamic changes in meat quality of the longissimus thoracis muscle in tibetan sheep at different growth stages. Front Vet Sci. 2022;9:926725. doi: 10.3389/fvets.2022.926725 35873690 PMC9298548

[72] Liu J, Shi L, Li Y, Chen L, Garrick D, Wang L, et al. Estimates of genomic inbreeding and identification of candidate regions that differ between Chinese indigenous sheep breeds. J Anim Sci Biotechnol. 2021;12(1):95. doi: 10.1186/s40104-021-00608-9 34348773 PMC8340518

[73] Huang S, Ma L, Li B, Dou J, Xu Q, Wang Y. Genomic analysis reveals population structure and selection signatures in plateau dairy cattle. BMC Genomics. 2025;26(1):240. doi: 10.1186/s12864-025-11335-0 40075267 PMC11905691

[74] Lin J, Jiang L, Wang X, Wei W, Song C, Cui Y, et al. P4HA2 promotes epithelial-to-mesenchymal transition and glioma malignancy through the collagen-dependent PI3K/AKT pathway. J Oncol. 2021;2021:1406853. doi: 10.1155/2021/1406853 34434233 PMC8382519

[75] Wu Y-L, Liu W, Zhao T, Jin J. P4HA2 contributes to head and neck squamous cell carcinoma progression and EMT through PI3K/AKT signaling pathway. Med Oncol. 2024;41(6):163. doi: 10.1007/s12032-024-02358-w 38777998 PMC11111551

[76] Kern RJ, Lindholm-Perry AK, Freetly HC, Snelling WM, Kern JW, Keele JW, et al. Transcriptome differences in the rumen of beef steers with variation in feed intake and gain. Gene. 2016;586(1):12–26. doi: 10.1016/j.gene.2016.03.034 27033587

[77] Xie F, Jin W, Si H, Yuan Y, Tao Y, Liu J, et al. An integrated gene catalog and over 10,000 metagenome-assembled genomes from the gastrointestinal microbiome of ruminants. Microbiome. 2021;9(1):137. doi: 10.1186/s40168-021-01078-x 34118976 PMC8199421

[78] Wallace RJ, Sasson G, Garnsworthy PC, Tapio I, Gregson E, Bani P, et al. A heritable subset of the core rumen microbiome dictates dairy cow productivity and emissions. Sci Adv. 2019;5(7):eaav8391. doi: 10.1126/sciadv.aav8391 31281883 PMC6609165

[79] Chen Y, Gondro C, Quinn K, Herd RM, Parnell PF, Vanselow B. Global gene expression profiling reveals genes expressed differentially in cattle with high and low residual feed intake. Anim Genet. 2011;42(5):475–90. doi: 10.1111/j.1365-2052.2011.02182.x 21906099

[80] Lima J, Martínez-Álvaro M, Mattock J, Auffret MD, Duthie C-A, Cleveland MA, et al. Temporal stability of the rumen microbiome and its longitudinal associations with performance traits in beef cattle. Sci Rep. 2024;14(1):20772. doi: 10.1038/s41598-024-70770-3 39237607 PMC11377694

[81] Tian R, Mahmoodi M, Tian J, Esmailizadeh Koshkoiyeh S, Zhao M, Saminzadeh M, et al. Leveraging functional genomics for understanding beef quality complexities and breeding beef cattle for improved meat quality. Genes (Basel). 2024;15(8):1104. doi: 10.3390/genes15081104 39202463 PMC11353656

[82] Buzanskas ME, Grossi DA, Ventura RV, Schenkel FS, Sargolzaei M, Meirelles SLC, et al. Genome-wide association for growth traits in Canchim beef cattle. PLoS One. 2014;9(4):e94802. doi: 10.1371/journal.pone.0094802 24733441 PMC3986245

[83] de Oliveira PSN, Cesar ASM, do Nascimento ML, Chaves AS, Tizioto PC, Tullio RR, et al. Identification of genomic regions associated with feed efficiency in Nelore cattle. BMC Genet. 2014;15:100. doi: 10.1186/s12863-014-0100-0 25257854 PMC4198703

[84] Grubbs JK, Fritchen AN, Huff-Lonergan E, Gabler NK, Lonergan SM. Selection for residual feed intake alters the mitochondria protein profile in pigs. J Proteomics. 2013;80:334–45. doi: 10.1016/j.jprot.2013.01.017 23403255

[85] Ramírez-Zamudio GD, Ganga MJG, Pereira GL, Nociti RP, Chiaratti MR, Cooke RF, et al. Effect of cow-calf supplementation on gene expression, processes, and pathways related to adipogenesis and lipogenesis in longissimus thoracis muscle of F1 Angus × Nellore cattle at weaning. Metabolites. 2023;13(2):160. doi: 10.3390/metabo13020160 36837780 PMC9962728

[86] Manzanilla-Pech CIV, De Haas Y, Hayes BJ, Veerkamp RF, Khansefid M, Donoghue KA, et al. Genomewide association study of methane emissions in Angus beef cattle with validation in dairy cattle. J Anim Sci. 2016;94(10):4151–66. doi: 10.2527/jas.2016-0431 27898855

[87] Robinson DL, Goopy JP, Hegarty RS, Oddy VH, Thompson AN, Toovey AF, et al. Genetic and environmental variation in methane emissions of sheep at pasture. J Anim Sci. 2014;92(10):4349–63. doi: 10.2527/jas.2014-8042 25149329

[88] López-Paredes J, Goiri I, Atxaerandio R, García-Rodríguez A, Ugarte E, Jiménez-Montero JA, et al. Mitigation of greenhouse gases in dairy cattle via genetic selection: 1. Genetic parameters of direct methane using noninvasive methods and proxies of methane. J Dairy Sci. 2020;103(8):7199–209. doi: 10.3168/jds.2019-17597 32475675

[89] Breider IS, Wall E, Garnsworthy PC. Short communication: Heritability of methane production and genetic correlations with milk yield and body weight in Holstein-Friesian dairy cows. J Dairy Sci. 2019;102(8):7277–81. doi: 10.3168/jds.2018-15909 31202647

[90] Pszczola M, Rzewuska K, Mucha S, Strabel T. Heritability of methane emissions from dairy cows over a lactation measured on commercial farms. J Anim Sci. 2017;95(11):4813–9. doi: 10.2527/jas2017.1842 29293701 PMC6292309

[91] Chipondoro L. Heritability of methane emission in Swedish Red cattle in northern Sweden. 2024.

[92] Ryan CV, Pabiou T, Purfield DC, Berry DP, Conroy S, Murphy CP, et al. Exploring definitions of daily enteric methane emission phenotypes for genetic evaluations using a population of indoor-fed multi-breed growing cattle with feed intake data. J Anim Sci. 2024;102:skae034. doi: 10.1093/jas/skae034 38323901 PMC10889735

[93] Ryan CV, Pabiou T, Purfield DC, Kelly DN, Murphy CP, Evans RD. Genetic correlations between enteric methane and traits of economic importance in a beef finishing system. J Anim Sci. 2025;103:skaf162. doi: 10.1093/jas/skaf162 40355361 PMC12132798

[94] Bittante G, Cecchinato A. Heritability estimates of enteric methane emissions predicted from fatty acid profiles, and their relationships with milk composition, cheese-yield and body size and condition. Ital J Anim Sci. 2019;19(1):114–26. doi: 10.1080/1828051x.2019.1698979

[95] Haas Y de, Windig JJ, Calus MPL, Dijkstra J, Haan M de, Bannink A, et al. Genetic parameters for predicted methane production and potential for reducing enteric emissions through genomic selection. J Dairy Sci. 2011;94(12):6122–34. doi: 10.3168/jds.2011-4439 22118100

[96] Løvendahl P, Difford GF, Li B, Chagunda MGG, Huhtanen P, Lidauer MH, et al. Review: selecting for improved feed efficiency and reduced methane emissions in dairy cattle. Animal. 2018;12(s2):s336–49. doi: 10.1017/S1751731118002276 30255826

[97] Sakamoto LS, Souza LL, Gianvecchio SB, de Oliveira MHV, Silva JAI de V, Canesin RC, et al. Phenotypic association among performance, feed efficiency and methane emission traits in Nellore cattle. PLoS One. 2021;16(10):e0257964. doi: 10.1371/journal.pone.0257964 34648502 PMC8516271

