# Supplementary material for: Genomic dissection of methane emission traits in cattle: A meta-GWAS and heritability analysis across populations
Source: PLoS One. 2026 Apr 10;21(4):e0344752. doi: 10.1371/journal.pone.0344752 (PMC13068272; doi:10.1371/journal.pone.0344752)
Supplement: S1 Table — (DOCX) [file pone.0344752.s002.docx]

| **Supplementary Table 1**- The characteristics of studies included in h^2^ meta- analysis. |
| --- |

| **Reference** | **Country/Region** | **Breed** | **P/G** | **Analysis method** | **Analysis**  **model** |
| --- | --- | --- | --- | --- | --- |
| Almasi et al. ^1^ | Australia | - | G | REML | Animal |
| Atashi et al. ^2^ | Belgium | Holstein | G | Bayesian | Animal |
| Atashi et al. ^3^ | Belgium | Holstein | G | Bayesian | Random Regression model |
| Bakke and Heringstad ^4^ | Norway | Norwegian Red | P | REML | Animal |
| Bittante and Cecchinato ^5^ | Italy | Brown Swiss | P | Bayesian | Animal |
| Bittante et al. ^6^ | Italy | Brown Swiss | P | Bayesian | Animal |
| Chen et al. ^7^ | Belgium | Holstein | G | REML | Animal |
| De Haas et al. ^8^ | Netherlands | Holstein-Friesian | P | REML | Sire-maternal grandsire |
| Difford et al. ^9^ | Denmark | Holstein | P | REML | Animal |
| Difford et al. ^10^ | Denmark and the Netherlands | Danish and Dutch | P | REML | Animal |
| Donoghue et al. ^11^ | Australia | Angus | P | REML | Animal |
| Donoghue et al. ^12^ | Australia | Angus | P | REML | Animal |
| Fresco et al. ^13^ | France | Holstein | P | REML | Animal |
| Fresco et al. ^14^ | France | Holstein | P | REML | Animal |
| Handcock et al. ^15^ | New Zealand | - | G | Bayesian | Animal |
| Hayes et al. ^16^ | Australia | Angus | G and P | REML | Animal |
| Johansen et al. ^17^ | Denmark | Holstein, Angus, Danish Blue, Charolais | P | Bayesian, REML | Animal |
| Kamalanathan et al. ^18^ | Canada | Holstein | P | REML | Animal |
| Kandel et al. ^19^ | Belgium | Holstein | P | Bayesian | Animal |
| Lassen and Løvendahl ^20^ | Denmark | Holstein | P | REML | Animal |
| **Reference** | **Country/Region** | **Breed** | **P/G** | **Analysis method** | **Analysis**  **model** |
| Layton et al. ^21^ | Italy | Holstein | G | Bayesian | Animal |
| Lassen et al. ^22^ | Denmark | Holstein | P | REML | Animal |
| Lekamp et al. ^23^ | United States of America | - | G | REML | Animal |
| Lopes et al. ^24^ | Canada | Holstein | P | REML | Animal |
| López-Paredes et al. ^25^ | Spain | Holstein | G | REML | Animal |
| Lopez-Paredes et al. ^26^ | Spain | Holstein | G | REML | Animal |
| Manzanilla-Pech et al. ^27^ | Australia | Holstein | G | GREML | Animal |
| Manzanilla-Pech et al. ^28^ | Denmark | Holstein | G | REML | Animal |
| Manzanilla-Pech et al. ^29^ | Australia, Denmark and Switzerland | Holstein | P | REML | Animal |
| Manzanilla-Pech et al. ^30^ | Denmark | Holstein | G | REML | Animal |
| Manzanilla-Pech et al. ^31^ | Denmark | Holstein | P | REML | Animal |
| Pickering et al. ^32^ | UK | Holstein | P | REML | Animal |
| Pszczola et al. ^33^ | Poland | Holstein | P | REML | Animal |
| Richardson et al. ^34^ | Australia | Holstein | p | REML | Animal |
| Ryan et al. ^35^ | Ireland | Mixed breeds | P | REML | Animal |
| Ryan et al. ^36^ | Ireland | Mixed breeds | P | REML | Animal |
| Saborío‐Montero et al. ^37^ | Spain | Holstein | G | Bayesian | Animal |
| Schneider et al.^38^ | Denmark | Holstein | P | REML | Animal |
| Sobrinho et al. ^39^ | Canada | Nellore | P | REML | Animal |
| Souza et al. ^40^ | Brazil | Nellore | G | REML | Animal |
| Sypniewski et al. ^41^ | Poland | Holstein | G | REML | Animal |
| Uemoto et al. ^42^ | Japan | Japanese Black steers | G and P | REML | Animal |
| Van Breukelen et al. ^43^ | Netherlands | Holstein | P | REML | Animal |
| **Reference** | **Country/Region** | **Breed** | **P/G** | **Analysis method** | **Analysis**  **model** |
| Van Breukelen et al. ^44^ | Netherlands | Holstein | P | REML | Random Regression model |
| Van Breukelen et al. ^45^ | Netherlands | Holstein | P | REML | Animal |
| Van Engelen et al. ^46^ | Netherlands | Holstein | P | REML | Animal |
| Vanrobays et al. ^47^ | Belgium | Holstein | P | REML | Animal |
| Visker et al. ^48^ | Netherlands | Holstein | P | REML | Animal |
| Yin et al. ^49^ | Switzerland | Brown Swiss | P | REML | Animal |
| Zetouni et al. ^50^ | Denmark | Holstein | P | REML | Animal |

P: Pedigree-based heritability; G: Genomic heritability; REML: Restricted Maximum Likelihood; GREML: Genome-based Restricted Maximum Likelihood

1 Almasi, F. *et al.* in *Proc. Assoc. Advmt. Anim. Breed. Genet.* 146-149.

2 Atashi, H. *et al.* in *WCGALP.* (WCGALP, Wageningen, Netherlands).

3 Atashi, H. *et al.* Genetic parameters of mid-infrared-predicted methane production and its relationship with production traits in Walloon Holstein dairy cows. *Journal of Dairy Science* (2025).

4 Bakke, K. & Heringstad, B. Genetic Correlations Between Daily Dry Matter Intake, Body Weight, and Enteric Methane in Norwegian Red. *Interbull Bulletin*, 78-85 (2024).

5 Bittante, G. & Cecchinato, A. Heritability estimates of enteric methane emissions predicted from fatty acid profiles, and their relationships with milk composition, cheese-yield and body size and condition. *Italian Journal of Animal Science* **19**, 114-126 (2020).

6 Bittante, G., Cipolat-Gotet, C. & Cecchinato, A. Genetic parameters of different FTIR-enabled phenotyping tools derived from milk fatty acid profile for reducing enteric methane emissions in dairy cattle. *Animals* **10**, 1654 (2020).

7 Chen, Y. *et al.* Exploring a Bayesian sparse factor model-based strategy for the genetic analysis of thousands of mid-infrared spectra traits for animal breeding. *Journal of Dairy Science* **107**, 9615-9627 (2024).

8 De Haas, Y. *et al.* Genetic parameters for predicted methane production and potential for reducing enteric emissions through genomic selection. *Journal of dairy science* **94**, 6122-6134 (2011).

9 Difford, G. F. *et al.* Host genetics and the rumen microbiome jointly associate with methane emissions in dairy cows. *PLoS genetics* **14**, e1007580 (2018).

10 Difford, G. F. *et al.* Can greenhouse gases in breath be used to genetically improve feed efficiency of dairy cows? *J Dairy Sci* **103**, 2442-2459 (2020). <https://doi.org:10.3168/jds.2019-16966>

11 Donoghue, K., Bird-Gardiner, T., Arthur, P., Herd, R. M. & Hegarty, R. Genetic and phenotypic variance and covariance components for methane emission and postweaning traits in Angus cattle. *Journal of Animal Science* **94**, 1438-1445 (2016).

12 Donoghue, K. A., Bird-Gardiner, T., Herd, R. M., Hegarty, R. S. & Arthur, P. F. Genetic variance and covariance components for carbon dioxide production and postweaning traits in Angus cattle. *Journal of Animal Science* **98**, skaa253 (2020).

13 Fresco, S., Boichard, D., Fritz, S. & Martin, P. Genetic parameters for methane production, intensity, and yield predicted from milk mid-infrared spectra throughout lactation in Holstein dairy cows. *Journal of Dairy Science* **107**, 11311-11323 (2024).

14 Fresco, S., Sanchez, M.-P., Boichard, D., Fritz, S. & Martin, P. Sequence-based GWAS reveals genes and variants associated with predicted methane emissions in French dairy cows. *Genetics Selection Evolution* **57**, 32 (2025).

15 Handcock, R. *et al.* in *Assoc. Adv. Anim. Breed. Genet.* 174-177.

16 Hayes, B. *et al.* Genomic heritabilities and genomic estimated breeding values for methane traits in Angus cattle. *Journal of Animal Science* **94**, 902-908 (2016).

17 Johansen, K. *et al.* Repeatability and genetic parameters for phenotypes of methane emission in crossbred beef× dairy slaughter calves. *animal* **19**, 101478 (2025).

18 Kamalanathan, S. *et al.* Genetic analysis of methane emission traits in Holstein dairy cattle. *Animals* **13**, 1308 (2023).

19 Kandel, P. *et al.* Genetic parameters of mid-infrared methane predictions and their relationships with milk production traits in Holstein cattle. *Journal of dairy science* **100**, 5578-5591 (2017).

20 Lassen, J. & Løvendahl, P. Heritability estimates for enteric methane emissions from Holstein cattle measured using noninvasive methods. *Journal of Dairy Science* **99**, 1959-1967 (2016).

21 Layton, J. *et al.* Genetic aspects of methane emissions and feed intake in growing Holstein bulls. *Italian Journal of Animal Science* **24**, 1909-1914 (2025).

22 Lassen, J., Poulsen, N., Larsen, M. K. & Buitenhuis, A. Genetic and genomic relationship between methane production measured in breath and fatty acid content in milk samples from Danish Holsteins. *Animal Production Science* **56**, 298-303 (2016).

23 Lakamp, A. D. *et al.* Variance component estimation and genome-wide association of predicted methane production in crossbred beef steers. *Journal of Animal Science* **101**, skad179 (2023).

24 Lopes, L. *et al.* Estimates of genetic parameters for rumination time, feed efficiency, and methane production traits in first-lactation Holstein cows. *Journal of dairy science* **107**, 4704-4713 (2024).

25 López-Paredes, J. *et al.* Mitigation of greenhouse gases in dairy cattle via genetic selection: 1. Genetic parameters of direct methane using noninvasive methods and proxies of methane. *Journal of dairy science* **103**, 7199-7209 (2020).

26 Lopez-Paredes, J., Saborio-Montero, A., Charfeddine, N., Jimenez-Montero, J. A. & Gonzalez-Recio, O. Dry matter intake, methane emissions and microbiome profiles as new traits for feed efficiency. *Interbull Bulletin*, 111-120 (2021).

27 Manzanilla-Pech, C. *et al.* Genomewide association study of methane emissions in Angus beef cattle with validation in dairy cattle. *Journal of Animal Science* **94**, 4151-4166 (2016).

28 Manzanilla-Pech, C. I. V., Gordo, D., Difford, G. F., Løvendahl, P. & Lassen, J. Multitrait genomic prediction of methane emissions in Danish Holstein cattle. *Journal of Dairy Science* **103**, 9195-9206 (2020).

29 Manzanilla-Pech, C. *et al.* Breeding for reduced methane emission and feed-efficient Holstein cows: An international response. *Journal of Dairy Science* **104**, 8983-9001 (2021).

30 Manzanilla-Pech, C. *et al.* Genome-wide association study for methane emission traits in Danish Holstein cattle. *Journal of Dairy Science* **105**, 1357-1368 (2022).

31 Manzanilla-Pech, C. I. V., Stephansen, R. B., Difford, G. F., Løvendahl, P. & Lassen, J. Selecting for feed efficient cows will help to reduce methane gas emissions. *Frontiers in Genetics* **13**, 885932 (2022).

32 Pickering, N. K. *et al.* Genetic parameters for predicted methane production and laser methane detector measurements. *Journal of Animal Science* **93**, 11-20 (2015).

33 Pszczola, M., Rzewuska, K., Mucha, S. & Strabel, T. Heritability of methane emissions from dairy cows over a lactation measured on commercial farms. *Journal of animal science* **95**, 4813-4819 (2017).

34 Richardson, C. *et al.* Genetic parameters for methane emission traits in Australian dairy cows. *Journal of Dairy Science* **104**, 539-549 (2021).

35 Ryan, C. V. *et al.* Exploring definitions of daily enteric methane emission phenotypes for genetic evaluations using a population of indoor-fed multi-breed growing cattle with feed intake data. *Journal of Animal Science* **102**, skae034 (2024).

36 Ryan, C. V. *et al.* Genetic correlations between enteric methane and traits of economic importance in a beef finishing system. *Journal of Animal Science* **103**, skaf162 (2025).

37 Saborío‐Montero, A. *et al.* Structural equation models to disentangle the biological relationship between microbiota and complex traits: Methane production in dairy cattle as a case of study. *Journal of Animal Breeding and Genetics* **137**, 36-48 (2020).

38 Schneider, H., Lajevardi, R. K., Bjerring, M., Sand, M. & Villumsen, T. M. Genetic parameters for enteric methane traits and their genetic connection with milk production in Danish Holstein cattle. *Journal of Dairy Science* (2025).

39 Sobrinho, T. *et al.* Heritability of predicted daily enteric methane emissions from growing Nellore cattle. *Genet. Mol. Res* **14**, 14123-14129 (2015).

40 Souza, L. L. *et al.* Heritability estimates and genome-wide association study of methane emission traits in Nellore cattle. *Journal of Animal Science* **102**, skae182 (2024).

41 Sypniewski, M., Strabel, T. & Pszczola, M. Genetic variability of methane production and concentration measured in the breath of Polish Holstein-Friesian cattle. *Animals* **11**, 3175 (2021).

42 Uemoto, Y. *et al.* Genetic and genomic analyses for predicted methane‐related traits in Japanese Black steers. *Animal Science Journal* **91**, e13383 (2020).

43 Van Breukelen, A. *et al.* Heritability and genetic correlations between enteric methane production and concentration recorded by GreenFeed and sniffers on dairy cows. *Journal of Dairy Science* **106**, 4121-4132 (2023).

44 Van Breukelen, A., Veerkamp, R., de Haas, Y. & Aldridge, M. Genetic parameter estimates for methane emission from breath during lactation and potential inaccuracies in reliabilities assuming a repeatability versus random regression model. *Journal of Dairy Science* **107**, 5853-5868 (2024).

45 van Breukelen, A., de Haas, Y., Aldridge, M., Meijer, N. & Veerkamp, R. Genetic relationships among methane emissions from breath, dry matter intake, body weight, and milk production traits of Dutch dairy cows. *Journal of Dairy Science* (2025).

46 Van Engelen, S., Bovenhuis, H., Dijkstra, J., Van Arendonk, J. & Visker, M. Genetic study of methane production predicted from milk fat composition in dairy cows. *Journal of Dairy Science* **98**, 8223-8226 (2015).

47 Vanrobays, M.-L. *et al.* Changes throughout lactation in phenotypic and genetic correlations between methane emissions and milk fatty acid contents predicted from milk mid-infrared spectra. *Journal of Dairy Science* **99**, 7247-7260 (2016).

48 Visker, M., van Engelen, S., Dijkstra, S., van Arendonk, J. & Bovenhuis, H. in *World Congress in Genetics Applied to Livestock Production (WCGALP), Vancouver, Canada.*

49 Yin, T., Pinent, T., Bruegemann, K., Simianer, H. & König, S. Simulation, prediction, and genetic analyses of daily methane emissions in dairy cattle. *Journal of Dairy Science* **98**, 5748-5762 (2015).

50 Zetouni, L., Kargo, M., Norberg, E. & Lassen, J. Genetic correlations between methane production and fertility, health, and body type traits in Danish Holstein cows. *Journal of dairy science* **101**, 2273-2280 (2018).
