# Supplementary material for: Genomic dissection of methane emission traits in cattle: A meta-GWAS and heritability analysis across populations
Source: PLoS One. 2026 Apr 10;21(4):e0344752. doi: 10.1371/journal.pone.0344752 (PMC13068272; doi:10.1371/journal.pone.0344752)
Supplement: S2 Table — (DOCX) [file pone.0344752.s003.docx]

| **Supplementary Table 2-** List of the identified genes related to significant SNPs. |
| --- |

| **Gene stable ID** | **Gene start (bp)** | **Gene end (bp)** | **Gene name** |
| --- | --- | --- | --- |
| ENSBTAG00000021193 | 89877735 | 89889302 | FBXO5 |
| ENSBTAG00000001116 | 22147142 | 22218911 | P4HA2 |
| ENSBTAG00000019217 | 39526080 | 39728759 | KIF13A |
| ENSBTAG00000013103 | 36457360 | 36475992 | COL1A1 |
| ENSBTAG00000003345 | 32346950 | 32529463 | FAT4 |
| ENSBTAG00000021007 | 51800170 | 51805078 | FOLR1 |
| ENSBTAG00000030671 | 85840597 | 86340597 | HOOK1 |
| ENSBTAG00000048804 | 53791937 | 53809246 | CUPIN1 |
| ENSBTAG00000002788 | 54084298 | 54260661 | RAB27B |
| ENSBTAG00000017573 | 54426079 | 54506698 | RBM26 |
| ENSBTAG00000043985 | 46050248 | 46548457 | DACH1 |
| ENSBTAG00000018575 | 1921450 | 2370583 | TRHDE |
| ENSBTAG00000007704 | 18454541 | 18531444 | ELOVL7 |
| ENSBTAG00000001509 | 60372175 | 60550376 | ELK3 |
| ENSBTAG00000013802: | 88677129 | 89159688 | DAB1 |
| ENSBTAG00000012307 | 22130555 | 22449866 | DTNA |
| ENSBTAG00000040568 | 37431139 | 37469998 | ZNF648 |
| ENSBTAG00000008032 | 115112608 | 115213732 | ACTR3B |
| ENSBTAG00000014871 | 1115631329 | 115629803 | CCT8L2 |
| ENSBTAG00000045535 | 115301688 | 115246102 | GALNTL5 |
| ENSBTAG00000021260 | 115360689 | 115335971 | GALNT11 |
| ENSBTAG00000046461 | 114631249 | 115631249 | SUMF1 |
| ENSBTAG00000031861 | 114855282 | 114803157 | RHEB |
| ENSBTAG00000003019 | 114779653 | 114773449 | CRYGN |
| ENSBTAG00000014372 | 114680160 | 114615444 | SMARCD3 |
| ENSBTAG00000004540 | 114725191 | 114689767 | NUB1 |
| ENSBTAG00000002917 | 114925565 | 114885501 | PRKAG2 |
| ENSBTAG00000024199 | 115524670 | 115368063 | KMT2C |
| ENSBTAG00000006232 | 114755250 | 114730382 | WDR86 |
| ENSBTAG00000000833 | 24977062 | 24971932 | TAX1BP3 |
| ENSBTAG00000007166 | 24494920 | 24523050 | CAMKK1 |
| ENSBTAG00000025121 | 24978133 | 24977284 | EMC6 |
| ENSBTAG00000015258 | 24996301 | 24983919 | P2RX5 |
| ENSBTAG00000008324 | 24481721 | 24480297 | LOC618593 |
| ENSBTAG00000020619 2 | 1626216 | 1665616 | PKD1 |
| ENSBTAG00000020031 | 36613501 | 36672487 | PKD2 |
| ENSBTAG00000000829 | 24951464 | 24933750 | SHPK |
| ENSBTAG00000000831 | 24972141 | 24951988 | CTNS |
| ENSBTAG00000000020 | 24885437 | 24854533 | TRPV3 |
| ENSBTAG00000018880 | 24913053 | 24890751 | TRPV1 |
| ENSBTAG00000017440 | 24023406 | 23961074 | METTL16 |
| ENSBTAG00000015763 | 24824295 | 24809409 | SPATA22 |
| ENSBTAG00000003629 | 24847108 | 24825866 | ASPA |
| ENSBTAG00000016806 | 24158415 | 24088805 | PAFAH1B1 |
| **Gene stable ID** | **Gene start (bp)** | **Gene end (bp)** | **Gene name** |
| ENSBTAG00000011786 | 24181331 | 24168990 | CLUH |
| ENSBTAG00000020000 | 24452796 | 24395974 | RAP1GAP2 |
| ENSBTAG00000045899 | 24763240 | 24762296 | LOC526294 |
| ENSBTAG00000032457 | 24802537 | 24801593 | OR3A3 |
| ENSBTAG00000039018 | 24507032 | 24506058 | OR1G1 |
| ENSBTAG00000040362 | 24521504 | 24520572 | LOC540082 |
| ENSBTAG00000039411 | 24528384 | 24527446 | LOC532238 |
| ENSBTAG00000039633 | 24539905 | 24538976 | LOC522582 |
| ENSBTAG00000046217 | 24750164 | 24749223 | LOC511509 |
| ENSBTAG00000047458 | 24756645 | 24755704 | LOC618112 |
| ENSBTAG00000048282 | 24599226 | 24598261 | LOC509525 |
| ENSBTAG00000045667 | 24731754 | 24730810 | LOC618124 |
| ENSBTAG00000046960 | 24724257 | 24723313 | LOC615901 |
| ENSBTAG00000047049 | 24632514 | 24631549 | LOC617122 |
| ENSBTAG00000048099 | 24613735 | 24612770 | LOC509526 |
| ENSBTAG00000026859 | 24655261 | 24654314 | LOC508980 |
| ENSBTAG00000018221 | 24669376 | 24668411 | LOC101902679 |
| ENSBTAG00000046652 | 24677969 | 24677022 | LOC538966 |
| ENSBTAG00000037529 | 24708485 | 24707451 | OR3A2 |
| ENSBTAG00000038059 | 24712313 | 24711369 | OR1E1 |
| ENSBTAG00000010791 | 24505109 | 24518593 | HTR3A |
| ENSBTAG00000001468 | 67064712 | 67289712 | SAMD4A |
| ENSBTAG00000030556 | 81854047 | 81863004 | ZNF217 |
| ENSBTAG00000000835 | 82162091 | 82259990 | BCAS1 |
| ENSBTAG00000007917 | 81601021 | 81604089 | TSHZ2 |
| ENSBTAG00000036111 | 21868711 | 21867759 | AP2M1 |
| ENSBTAG00000000977 | 25714094 | 26071222 | CADM1 |
| ENSBTAG00000031362 | 25524857 | 25515542 | NXPE2 |
| ENSBTAG00000005945 | 25434051 | 25419219 | NXPE4 |
| ENSBTAG00000005843 | 25304051 | 25293299 | REXO2 |
| ENSBTAG00000046256 | 48416690 | 48868792 | TMEM132C |
| ENSBTAG00000007306 | 20440181 | 20735986 | RAB3C |
| ENSBTAG00000005644 | 44853997 | 44970388 | GALNT9 |
| ENSBTAG00000016514 | 573305 | 721444 | CPE |
| ENSBTAG00000051724 | 104316388 | 104728748 | TMEM178B |
| ENSBTAG00000021514 | 107307884 | 107331666 | DNPEP |
| ENSBTAG00000032092 | 32682891 | 32772981 | CDH11 |
| ENSBTAG00000030942 | 138384476 | 138464624 | MRPL3 |
| ENSBTAG00000015837 | 116694444 | 116747331 | P2RY12 |
| ENSBTAG00000021703 | 116653939 | 117017709 | MED12L |
| ENSBTAG00000026323 | 44347203 | 44352876 | LYSB |
| ENSBTAG00000026088 | 44365812 | 44371561 | LYZ2 |
| ENSBTAG00000046511 | 44392339 | 44400094 | LYZ1 |
| ENSBTAG00000048493 | 91435008 | 92802345 | NAALADL2 |
| ENSBTAG00000023429 | 126325171 | 126418835 | PLS1 |
| ENSBTAG00000017244 | 28229476 | 28341207 | PRPF18 |
| ENSBTAG00000061592 | 28242451 | 28399691 | YTHDF3 |
| **Gene stable ID** | **Gene start (bp)** | **Gene end (bp)** | **Gene name** |
| ENSBTAG00000021941 | 115834010 | 116899609 | DPP6 |
| ENSBTAG00000019052 | 15696541 | 16446156 | ANK3 |
| ENSBTAG00000001992 | 9434458 | 9476713 | CYP51A1 |
| ENSBTAG00000009646 | 27282280 | 27326576 | ALDH7A1 |
| ENSBTAG00000007705 | 35248809 | 35434069 | COLEC12 |
| ENSBTAG00000011225 | 44057524 | 44070810 | TMX1 |
| ENSBTAG00000020617 | 1595329 | 1626216 | TSC2 |
| ENSBTAG00000001274 | 106404112 | 106727269 | PPM1L |
| ENSBTAG00000019265 | 104801940 | 104902617 | AGK |
| ENSBTAG00000010394 | 83653588 | 83911819 | MCF2L2 |
| ENSBTAG00000031178 | 125043158 | 125706579 | SLC9A9 |
| ENSBTAG00000005871 | 97692611 | 98348910 | MECOM |
| ENSBTAG00000043962 | 40812649 | 41332738 | SLC2A13 |
| ENSBTAG000000323661 | 47209404 | 47348038 | SLC23A2 |
| ENSBTAG000000485601 | 55796086 | 55830485 | TMEM233 |
| ENSBTAG000000157391 | 47043142 | 47103169 | MRC2 |
| ENSBTAG000000070712 | 39313837 | 39475310 | RAI14 |
| ENSBTAG000000006531 | 67628341 | 67706157 | PPP1R16B |
| ENSBTAG00000051030 | 86053854 | 86085716 | CYP2J30 |
| ENSBTAG00000018546 | 6800432 | 7556710 | LRBA |
| ENSBTAG00000017986 | 92479465 | 92739972 | GLIS1 |
| ENSBTAG00000012444 | 45512771 | 45903613 | ADAM12 |
