# Supplementary material for: Genomic dissection of methane emission traits in cattle: A meta-GWAS and heritability analysis across populations
Source: PLoS One. 2026 Apr 10;21(4):e0344752. doi: 10.1371/journal.pone.0344752 (PMC13068272; doi:10.1371/journal.pone.0344752)
Supplement: S4 Table — (DOCX) [file pone.0344752.s005.docx]

| **Supplementary Table 4-** Genemania Interactions | | | | |
| --- | --- | --- | --- | --- |
| **Gene 1** | **Gene 2** | **Weight** | **Network group** | **Network** |
| PRKAG2 | SAMD4A | 0.022259 | Co-expression | Dobbin-Giordano-2005 |
| PRKAG2 | DTNA | 0.018422 | Co-expression | Dobbin-Giordano-2005 |
| EMC6 | RAP1GAP2 | 0.009933 | Co-expression | Dobbin-Giordano-2005 |
| MRC2 | ADAM12 | 0.016553 | Co-expression | Dobbin-Giordano-2005 |
| NTHL1 | SHPK | 0.021101 | Co-expression | Dobbin-Giordano-2005 |
| NTHL1 | EMC6 | 0.022832 | Co-expression | Dobbin-Giordano-2005 |
| ALDH7A1 | SHPK | 0.031123 | Co-expression | Dobbin-Giordano-2005 |
| ALDH7A1 | NTHL1 | 0.02227 | Co-expression | Dobbin-Giordano-2005 |
| ZNF217 | BCAS1 | 0.015048 | Co-expression | Dobbin-Giordano-2005 |
| CYP2J2 | CLUH | 0.006827 | Co-expression | Dobbin-Giordano-2005 |
| MRPL3 | REXO2 | 0.011591 | Co-expression | Dobbin-Giordano-2005 |
| COL1A1 | MRC2 | 0.015085 | Co-expression | Dobbin-Giordano-2005 |
| BNIP3 | SHPK | 0.026999 | Co-expression | Dobbin-Giordano-2005 |
| BNIP3 | PLS1 | 0.011752 | Co-expression | Dobbin-Giordano-2005 |
| BNIP3 | NTHL1 | 0.022012 | Co-expression | Dobbin-Giordano-2005 |
| BNIP3 | ALDH7A1 | 0.023165 | Co-expression | Dobbin-Giordano-2005 |
| BMP1 | MRC2 | 0.0141 | Co-expression | Dobbin-Giordano-2005 |
| PKD2 | COLEC12 | 0.01055 | Co-expression | Dobbin-Giordano-2005 |
| PKD2 | YTHDF3 | 0.006208 | Co-expression | Dobbin-Giordano-2005 |
| ERP44 | TSC1 | 0.016108 | Co-expression | Dobbin-Giordano-2005 |
| AASS | CYP2J2 | 0.008774 | Co-expression | Dobbin-Giordano-2005 |
| CTNS | CLUH | 0.01443 | Co-expression | Bild-Nevins-2006 B |
| DACH1 | PLS1 | 0.014681 | Co-expression | Bild-Nevins-2006 B |
| LRBA | BCAS1 | 0.007786 | Co-expression | Bild-Nevins-2006 B |
| ZNF217 | BCAS1 | 0.013867 | Co-expression | Bild-Nevins-2006 B |
| MRPL3 | RHEB | 0.009621 | Co-expression | Bild-Nevins-2006 B |
| TSC2 | DNPEP | 0.008323 | Co-expression | Bild-Nevins-2006 B |
| TSC2 | NTHL1 | 0.008659 | Co-expression | Bild-Nevins-2006 B |
| CDH11 | SAMD4A | 0.008433 | Co-expression | Bild-Nevins-2006 B |
| CDH11 | CADM1 | 0.007473 | Co-expression | Bild-Nevins-2006 B |
| CDH11 | ADAM12 | 0.008378 | Co-expression | Bild-Nevins-2006 B |
| CDH11 | RAI14 | 0.005109 | Co-expression | Bild-Nevins-2006 B |
| CPE | CDH11 | 0.008585 | Co-expression | Bild-Nevins-2006 B |
| COL1A1 | RAI14 | 0.012048 | Co-expression | Bild-Nevins-2006 B |
| AP2M1 | MRC2 | 0.007569 | Co-expression | Bild-Nevins-2006 B |
| PAFAH1B1 | RAP1GAP2 | 0.021418 | Co-expression | Bild-Nevins-2006 B |
| BNIP3 | RHEB | 0.029779 | Co-expression | Bild-Nevins-2006 B |
| BMP1 | MRC2 | 0.012926 | Co-expression | Bild-Nevins-2006 B |
| PKD2 | SAMD4A | 0.022473 | Co-expression | Bild-Nevins-2006 B |
| PKD2 | CDH11 | 0.008591 | Co-expression | Bild-Nevins-2006 B |
| CTNNB1 | AP2M1 | 0.014089 | Co-expression | Bild-Nevins-2006 B |
| NSA2 | TMX1 | 0.007293 | Co-expression | Bild-Nevins-2006 B |
| AASS | RAP1GAP2 | 0.020773 | Co-expression | Bild-Nevins-2006 B |
| AASS | ALDH7A1 | 0.021794 | Co-expression | Bild-Nevins-2006 B |
| AASS | ASPA | 0.018694 | Co-expression | Bild-Nevins-2006 B |
| CLIP1 | RAI14 | 0.007023 | Co-expression | Bild-Nevins-2006 B |
| CLIP1 | ZNF217 | 0.009425 | Co-expression | Bild-Nevins-2006 B |
| HTR3A | MCF2L2 | 0.024911 | Co-expression | Wang-Maris-2006 |
| RAP1GAP2 | CADM1 | 0.007331 | Co-expression | Wang-Maris-2006 |
| DACH1 | PPP1R16B | 0.01907 | Co-expression | Wang-Maris-2006 |
| LRBA | NTHL1 | 0.008745 | Co-expression | Wang-Maris-2006 |
| DPP6 | RAP1GAP2 | 0.01002 | Co-expression | Wang-Maris-2006 |
| ALDH7A1 | NTHL1 | 0.010065 | Co-expression | Wang-Maris-2006 |
| ASPA | SAMD4A | 0.016525 | Co-expression | Wang-Maris-2006 |
| MRPL3 | PLS1 | 0.007641 | Co-expression | Wang-Maris-2006 |
| MECOM | MRC2 | 0.013647 | Co-expression | Wang-Maris-2006 |
| RAB11FIP3 | ZNF217 | 0.015258 | Co-expression | Wang-Maris-2006 |
| CTNNB1 | ZNF217 | 0.019555 | Co-expression | Wang-Maris-2006 |
| PAFAH1B2 | PKD2 | 0.021141 | Co-expression | Wang-Maris-2006 |
| ERP44 | TSC1 | 0.016197 | Co-expression | Wang-Maris-2006 |
| NSA2 | ALDH7A1 | 0.015795 | Co-expression | Wang-Maris-2006 |
| NSA2 | MRPL3 | 0.005064 | Co-expression | Wang-Maris-2006 |
| AASS | ZNF217 | 0.024355 | Co-expression | Wang-Maris-2006 |
| CRYGN | WDR86 | 0.013512 | Co-expression | Roth-Zlotnik-2006 |
| P2RY12 | SLC9A9 | 0.013059 | Co-expression | Roth-Zlotnik-2006 |
| NXPE4 | TESC | 0.014564 | Co-expression | Roth-Zlotnik-2006 |
| DTNA | P2RY12 | 0.011462 | Co-expression | Roth-Zlotnik-2006 |
| COLEC12 | TMEM132C | 0.018502 | Co-expression | Roth-Zlotnik-2006 |
| TMX1 | PRPF18 | 0.012917 | Co-expression | Roth-Zlotnik-2006 |
| EMC6 | SAMD4A | 0.011393 | Co-expression | Roth-Zlotnik-2006 |
| BCAS1 | P2RY12 | 0.004242 | Co-expression | Roth-Zlotnik-2006 |
| LRBA | HOOK1 | 0.011304 | Co-expression | Roth-Zlotnik-2006 |
| LRBA | PLS1 | 0.008947 | Co-expression | Roth-Zlotnik-2006 |
| ASPA | BCAS1 | 0.005178 | Co-expression | Roth-Zlotnik-2006 |
| CYP2J2 | BCAS1 | 0.005694 | Co-expression | Roth-Zlotnik-2006 |
| MRPL3 | HOOK1 | 0.00745 | Co-expression | Roth-Zlotnik-2006 |
| MRPL3 | PRPF18 | 0.006413 | Co-expression | Roth-Zlotnik-2006 |
| CDH11 | RAI14 | 0.015135 | Co-expression | Roth-Zlotnik-2006 |
| COL1A1 | MRC2 | 0.015888 | Co-expression | Roth-Zlotnik-2006 |
| MECOM | PLS1 | 0.017119 | Co-expression | Roth-Zlotnik-2006 |
| RAB11FIP3 | GALNT11 | 0.018284 | Co-expression | Roth-Zlotnik-2006 |
| BNIP3 | RHEB | 0.017632 | Co-expression | Roth-Zlotnik-2006 |
| AKAP5 | ACTR3B | 0.009537 | Co-expression | Roth-Zlotnik-2006 |
| PKD2 | COLEC12 | 0.009056 | Co-expression | Roth-Zlotnik-2006 |
| PKD2 | RAI14 | 0.007294 | Co-expression | Roth-Zlotnik-2006 |
| NSA2 | ITGAE | 0.010656 | Co-expression | Roth-Zlotnik-2006 |
| AASS | ALDH7A1 | 0.027607 | Co-expression | Roth-Zlotnik-2006 |
| AASS | PKD2 | 0.009076 | Co-expression | Roth-Zlotnik-2006 |
| CRYGN | WDR86 | 0.017398 | Co-expression | Mallon-McKay-2013 |
| GALNTL5 | SPATA22 | 0.0087 | Co-expression | Mallon-McKay-2013 |
| NXPE4 | NXPE2 | 0.016257 | Co-expression | Mallon-McKay-2013 |
| GALNT11 | ELOVL7 | 0.019781 | Co-expression | Mallon-McKay-2013 |
| PPP1R16B | P2RY12 | 0.023683 | Co-expression | Mallon-McKay-2013 |
| ELK3 | COLEC12 | 0.0075 | Co-expression | Mallon-McKay-2013 |
| MRC2 | TMEM132C | 0.008216 | Co-expression | Mallon-McKay-2013 |
| MRC2 | COLEC12 | 0.005335 | Co-expression | Mallon-McKay-2013 |
| BCAS1 | CAMKK1 | 0.013799 | Co-expression | Mallon-McKay-2013 |
| ITGAE | EMC6 | 0.010962 | Co-expression | Mallon-McKay-2013 |
| TSC2 | KMT2C | 0.0083 | Co-expression | Mallon-McKay-2013 |
| TSC2 | DNPEP | 0.008985 | Co-expression | Mallon-McKay-2013 |
| CDH11 | TMEM132C | 0.01228 | Co-expression | Mallon-McKay-2013 |
| CDH11 | COLEC12 | 0.007739 | Co-expression | Mallon-McKay-2013 |
| COL1A1 | TMEM132C | 0.011198 | Co-expression | Mallon-McKay-2013 |
| RAB11FIP3 | MRC2 | 0.005269 | Co-expression | Mallon-McKay-2013 |
| BNIP3 | P4HA2 | 0.016072 | Co-expression | Mallon-McKay-2013 |
| BMP1 | MRC2 | 0.011762 | Co-expression | Mallon-McKay-2013 |
| CTNNB1 | YTHDF3 | 0.011145 | Co-expression | Mallon-McKay-2013 |
| TSC1 | ANK3 | 0.00951 | Co-expression | Mallon-McKay-2013 |
| AASS | METTL16 | 0.008719 | Co-expression | Mallon-McKay-2013 |
| AASS | RAP1GAP2 | 0.014707 | Co-expression | Mallon-McKay-2013 |
| MED12 | KMT2C | 0.010507 | Co-expression | Mallon-McKay-2013 |
| MED12 | TSC2 | 0.00397 | Co-expression | Mallon-McKay-2013 |
| NUB1 | KMT2C | 0.015946 | Co-expression | Chen-Brown-2002 |
| ADAM12 | COLEC12 | 0.017842 | Co-expression | Chen-Brown-2002 |
| PRPF18 | ACTR3B | 0.016145 | Co-expression | Chen-Brown-2002 |
| EMC6 | CTNS | 0.021033 | Co-expression | Chen-Brown-2002 |
| MRC2 | TAX1BP3 | 0.004742 | Co-expression | Chen-Brown-2002 |
| ALDH7A1 | KIF13A | 0.012008 | Co-expression | Chen-Brown-2002 |
| ALDH7A1 | CADM1 | 0.015338 | Co-expression | Chen-Brown-2002 |
| MRPL3 | SHPK | 0.011717 | Co-expression | Chen-Brown-2002 |
| PAFAH1B1 | SLC23A2 | 0.007905 | Co-expression | Chen-Brown-2002 |
| BMP1 | PAFAH1B1 | 0.013119 | Co-expression | Chen-Brown-2002 |
| PKD2 | PRKAG2 | 0.014483 | Co-expression | Chen-Brown-2002 |
| PKD2 | ASPA | 0.022285 | Co-expression | Chen-Brown-2002 |
| CTNNB1 | CYP51A1 | 0.025091 | Co-expression | Chen-Brown-2002 |
| MED12 | PPM1L | 0.012451 | Co-expression | Chen-Brown-2002 |
| MCF2L2 | NXPE4 | 0.023867 | Co-expression | Wang-Cheung-2015 |
| BCAS1 | TESC | 0.010588 | Co-expression | Wang-Cheung-2015 |
| ALDH7A1 | CADM1 | 0.017161 | Co-expression | Wang-Cheung-2015 |
| RAI14 | RAP1GAP2 | 0.010623 | Co-expression | Wang-Cheung-2015 |
| ASPA | BCAS1 | 0.013563 | Co-expression | Wang-Cheung-2015 |
| MRPL3 | DNPEP | 0.00434 | Co-expression | Wang-Cheung-2015 |
| MRPL3 | ACTR3B | 0.003604 | Co-expression | Wang-Cheung-2015 |
| DAB1 | COLEC12 | 0.010597 | Co-expression | Wang-Cheung-2015 |
| CPE | REXO2 | 0.006056 | Co-expression | Wang-Cheung-2015 |
| MECOM | SMARCD3 | 0.011656 | Co-expression | Wang-Cheung-2015 |
| RAB11FIP3 | AP2M1 | 0.005052 | Co-expression | Wang-Cheung-2015 |
| BNIP3 | REXO2 | 0.01073 | Co-expression | Wang-Cheung-2015 |
| RPH3AL | AP2M1 | 0.00286 | Co-expression | Wang-Cheung-2015 |
| PKD2 | CADM1 | 0.016022 | Co-expression | Wang-Cheung-2015 |
| PKD2 | ALDH7A1 | 0.011462 | Co-expression | Wang-Cheung-2015 |
| LRP8 | CLUH | 0.004274 | Co-expression | Wang-Cheung-2015 |
| LRP8 | ITGAE | 0.003193 | Co-expression | Wang-Cheung-2015 |
| PAFAH1B2 | PKD2 | 0.011613 | Co-expression | Wang-Cheung-2015 |
| PAFAH1B2 | CTNNB1 | 0.022275 | Co-expression | Wang-Cheung-2015 |
| TESC | ELOVL7 | 0.008894 | Co-expression | Innocenti-Brown-2011 |
| RAP1GAP2 | GALNTL5 | 0.023583 | Co-expression | Innocenti-Brown-2011 |
| PRPF18 | ACTR3B | 0.012817 | Co-expression | Innocenti-Brown-2011 |
| TMX1 | PRPF18 | 0.007899 | Co-expression | Innocenti-Brown-2011 |
| BCAS1 | TRPV3 | 0.006959 | Co-expression | Innocenti-Brown-2011 |
| PLS1 | HOOK1 | 0.010608 | Co-expression | Innocenti-Brown-2011 |
| RAI14 | PLS1 | 0.012256 | Co-expression | Innocenti-Brown-2011 |
| ANK3 | DACH1 | 0.019613 | Co-expression | Innocenti-Brown-2011 |
| SMARCD3 | TESC | 0.002639 | Co-expression | Innocenti-Brown-2011 |
| MRPL3 | ACTR3B | 0.015478 | Co-expression | Innocenti-Brown-2011 |
| MRPL3 | PRPF18 | 0.006995 | Co-expression | Innocenti-Brown-2011 |
| DAB1 | HTR3A | 0.003906 | Co-expression | Innocenti-Brown-2011 |
| AP2M1 | TAX1BP3 | 0.004112 | Co-expression | Innocenti-Brown-2011 |
| AP2M1 | RAI14 | 0.004955 | Co-expression | Innocenti-Brown-2011 |
| PAFAH1B1 | PRPF18 | 0.01104 | Co-expression | Innocenti-Brown-2011 |
| PAFAH1B1 | RHEB | 0.021769 | Co-expression | Innocenti-Brown-2011 |
| RAB11FIP3 | TSC2 | 0.00712 | Co-expression | Innocenti-Brown-2011 |
| BNIP3 | CYP2J2 | 0.004498 | Co-expression | Innocenti-Brown-2011 |
| CTLA4 | DTNA | 0.007577 | Co-expression | Innocenti-Brown-2011 |
| CTLA4 | MCF2L2 | 0.007439 | Co-expression | Innocenti-Brown-2011 |
| RPH3AL | PRKAG2 | 0.01082 | Co-expression | Innocenti-Brown-2011 |
| RPH3AL | TSC2 | 0.004521 | Co-expression | Innocenti-Brown-2011 |
| AKAP5 | NXPE2 | 0.009916 | Co-expression | Innocenti-Brown-2011 |
| SIX1 | ELOVL7 | 0.021076 | Co-expression | Innocenti-Brown-2011 |
| DAB2IP | RAB11FIP3 | 0.021855 | Co-expression | Innocenti-Brown-2011 |
| ERP44 | HOOK1 | 0.022635 | Co-expression | Innocenti-Brown-2011 |
| MED12 | AP2M1 | 0.004362 | Co-expression | Innocenti-Brown-2011 |
| DPP6 | SLC23A2 | 0.01212 | Co-expression | Perou-Botstein-1999 |
| ANK3 | ADAM12 | 0.016299 | Co-expression | Perou-Botstein-1999 |
| COL1A1 | CPE | 0.011883 | Co-expression | Perou-Botstein-1999 |
| CTNNB1 | COL1A1 | 0.013093 | Co-expression | Perou-Botstein-1999 |
| SLC23A2 | RBM26 | 0.012015 | Co-expression | Ross-Perou-2001 |
| BCAS1 | COLEC12 | 0.015746 | Co-expression | Ross-Perou-2001 |
| ASPA | REXO2 | 0.011997 | Co-expression | Ross-Perou-2001 |
| SMARCD3 | DPP6 | 0.018395 | Co-expression | Ross-Perou-2001 |
| COL1A1 | CDH11 | 0.009028 | Co-expression | Ross-Perou-2001 |
| BMP1 | ASPA | 0.006258 | Co-expression | Ross-Perou-2001 |
| BMP1 | CPE | 0.008921 | Co-expression | Ross-Perou-2001 |
| PKD2 | ASPA | 0.008558 | Co-expression | Ross-Perou-2001 |
| PKD2 | BMP1 | 0.006456 | Co-expression | Ross-Perou-2001 |
| CTNNB1 | REXO2 | 0.017822 | Co-expression | Ross-Perou-2001 |
| CTNNB1 | CPE | 0.016189 | Co-expression | Ross-Perou-2001 |
| PAFAH1B2 | KIF13A | 0.018876 | Co-expression | Ross-Perou-2001 |
| COLEC12 | RBM26 | 0.006514 | Co-expression | Arijs-Rutgeerts-2009 |
| PLS1 | SLC2A13 | 0.010249 | Co-expression | Arijs-Rutgeerts-2009 |
| RAI14 | SAMD4A | 0.015326 | Co-expression | Arijs-Rutgeerts-2009 |
| ZNF217 | RHEB | 0.009343 | Co-expression | Arijs-Rutgeerts-2009 |
| CYP2J2 | ELOVL7 | 0.006732 | Co-expression | Arijs-Rutgeerts-2009 |
| CYP2J2 | PRKAG2 | 0.008311 | Co-expression | Arijs-Rutgeerts-2009 |
| CYP2J2 | HOOK1 | 0.005304 | Co-expression | Arijs-Rutgeerts-2009 |
| CYP2J2 | ASPA | 0.004201 | Co-expression | Arijs-Rutgeerts-2009 |
| CPE | ELOVL7 | 0.024534 | Co-expression | Arijs-Rutgeerts-2009 |
| CPE | DAB1 | 0.012122 | Co-expression | Arijs-Rutgeerts-2009 |
| MECOM | GALNT11 | 0.010612 | Co-expression | Arijs-Rutgeerts-2009 |
| MECOM | LRBA | 0.020387 | Co-expression | Arijs-Rutgeerts-2009 |
| AKAP5 | BCAS1 | 0.02093 | Co-expression | Arijs-Rutgeerts-2009 |
| BMP1 | MRC2 | 0.02599 | Co-expression | Arijs-Rutgeerts-2009 |
| PKD2 | COLEC12 | 0.00314 | Co-expression | Arijs-Rutgeerts-2009 |
| TSC1 | KMT2C | 0.008964 | Co-expression | Arijs-Rutgeerts-2009 |
| TSC1 | TSC2 | 0.009071 | Co-expression | Arijs-Rutgeerts-2009 |
| NSA2 | MRPL3 | 0.003463 | Co-expression | Arijs-Rutgeerts-2009 |
| AASS | PKD2 | 0.007057 | Co-expression | Arijs-Rutgeerts-2009 |
| MED12 | TSC1 | 0.010258 | Co-expression | Arijs-Rutgeerts-2009 |
| HOOK1 | REXO2 | 0.007295 | Co-expression | Ramaswamy-Golub-2001 |
| YTHDF3 | PRPF18 | 0.007692 | Co-expression | Ramaswamy-Golub-2001 |
| ALDH7A1 | LRBA | 0.008592 | Co-expression | Ramaswamy-Golub-2001 |
| ZNF217 | PRPF18 | 0.006315 | Co-expression | Ramaswamy-Golub-2001 |
| SMARCD3 | DTNA | 0.006441 | Co-expression | Ramaswamy-Golub-2001 |
| MRPL3 | CYP2J2 | 0.013257 | Co-expression | Ramaswamy-Golub-2001 |
| CDH11 | P4HA2 | 0.018244 | Co-expression | Ramaswamy-Golub-2001 |
| CPE | LRBA | 0.006102 | Co-expression | Ramaswamy-Golub-2001 |
| BNIP3 | ALDH7A1 | 0.010361 | Co-expression | Ramaswamy-Golub-2001 |
| AKAP5 | SMARCD3 | 0.004643 | Co-expression | Ramaswamy-Golub-2001 |
| SIX1 | ALDH7A1 | 0.009219 | Co-expression | Ramaswamy-Golub-2001 |
| SIX1 | CPE | 0.007112 | Co-expression | Ramaswamy-Golub-2001 |
| BMP1 | SMARCD3 | 0.004184 | Co-expression | Ramaswamy-Golub-2001 |
| PKD2 | ZNF217 | 0.018308 | Co-expression | Ramaswamy-Golub-2001 |
| PKD2 | PAFAH1B1 | 0.010307 | Co-expression | Ramaswamy-Golub-2001 |
| CTNNB1 | PKD1 | 0.012582 | Co-expression | Ramaswamy-Golub-2001 |
| PAFAH1B2 | PRPF18 | 0.007246 | Co-expression | Ramaswamy-Golub-2001 |
| ASPA | DPP6 | 0.00916 | Co-localization | Schadt-Shoemaker-2004 |
| ASPA | ALDH7A1 | 0.0118 | Co-localization | Schadt-Shoemaker-2004 |
| ASPA | ANK3 | 0.009413 | Co-localization | Schadt-Shoemaker-2004 |
| CYP2J2 | ANK3 | 0.010076 | Co-localization | Schadt-Shoemaker-2004 |
| CDH11 | ASPA | 0.008108 | Co-localization | Schadt-Shoemaker-2004 |
| CPE | DPP6 | 0.008123 | Co-localization | Schadt-Shoemaker-2004 |
| CPE | ALDH7A1 | 0.008469 | Co-localization | Schadt-Shoemaker-2004 |
| CPE | ANK3 | 0.007529 | Co-localization | Schadt-Shoemaker-2004 |
| CPE | ASPA | 0.006441 | Co-localization | Schadt-Shoemaker-2004 |
| CPE | CDH11 | 0.006236 | Co-localization | Schadt-Shoemaker-2004 |
| PAFAH1B1 | DPP6 | 0.008144 | Co-localization | Schadt-Shoemaker-2004 |
| PAFAH1B1 | ANK3 | 0.007844 | Co-localization | Schadt-Shoemaker-2004 |
| PAFAH1B1 | ASPA | 0.005659 | Co-localization | Schadt-Shoemaker-2004 |
| PAFAH1B1 | CDH11 | 0.005882 | Co-localization | Schadt-Shoemaker-2004 |
| PAFAH1B1 | CPE | 0.005334 | Co-localization | Schadt-Shoemaker-2004 |
| CTNNB1 | CDH11 | 0.012681 | Co-localization | Schadt-Shoemaker-2004 |
| CTNNB1 | CPE | 0.006653 | Co-localization | Schadt-Shoemaker-2004 |
| TSC1 | DPP6 | 0.011157 | Co-localization | Schadt-Shoemaker-2004 |
| TSC1 | ANK3 | 0.011242 | Co-localization | Schadt-Shoemaker-2004 |
| TSC1 | PAFAH1B1 | 0.00901 | Co-localization | Schadt-Shoemaker-2004 |
| DTNA | P2RY12 | 0.005991 | Co-localization | Johnson-Shoemaker-2003 |
| BCAS1 | P2RY12 | 0.00735 | Co-localization | Johnson-Shoemaker-2003 |
| PLS1 | NXPE4 | 0.014293 | Co-localization | Johnson-Shoemaker-2003 |
| ALDH7A1 | GALNT11 | 0.013513 | Co-localization | Johnson-Shoemaker-2003 |
| RAI14 | ADAM12 | 0.007414 | Co-localization | Johnson-Shoemaker-2003 |
| ZNF217 | TMX1 | 0.006474 | Co-localization | Johnson-Shoemaker-2003 |
| ASPA | P2RY12 | 0.003177 | Co-localization | Johnson-Shoemaker-2003 |
| ASPA | DTNA | 0.007316 | Co-localization | Johnson-Shoemaker-2003 |
| ASPA | BCAS1 | 0.008831 | Co-localization | Johnson-Shoemaker-2003 |
| CDH11 | ELK3 | 0.015097 | Co-localization | Johnson-Shoemaker-2003 |
| CDH11 | DACH1 | 0.016783 | Co-localization | Johnson-Shoemaker-2003 |
| CPE | DPP6 | 0.003504 | Co-localization | Johnson-Shoemaker-2003 |
| CPE | ANK3 | 0.00837 | Co-localization | Johnson-Shoemaker-2003 |
| COL1A1 | CDH11 | 0.008632 | Co-localization | Johnson-Shoemaker-2003 |
| RAB11FIP3 | GALNT11 | 0.020641 | Co-localization | Johnson-Shoemaker-2003 |
| TSC1 | PAFAH1B1 | 0.013778 | Co-localization | Johnson-Shoemaker-2003 |
| CLIP1 | SIX1 | 0.006961 | Co-localization | Johnson-Shoemaker-2003 |
| CRYGN | SLC9A9 | 0.000605 | Genetic Interactions | Lin-Smith-2010 |
| CYP51A1 | NXPE2 | 0.000615 | Genetic Interactions | Lin-Smith-2010 |
| CYP51A1 | SLC9A9 | 0.000299 | Genetic Interactions | Lin-Smith-2010 |
| KMT2C | SLC9A9 | 0.000433 | Genetic Interactions | Lin-Smith-2010 |
| KMT2C | MED12L | 0.000438 | Genetic Interactions | Lin-Smith-2010 |
| KMT2C | ELOVL7 | 0.000578 | Genetic Interactions | Lin-Smith-2010 |
| TSHZ2 | ELOVL7 | 0.000348 | Genetic Interactions | Lin-Smith-2010 |
| NUB1 | NXPE2 | 0.001035 | Genetic Interactions | Lin-Smith-2010 |
| NUB1 | NAALADL2 | 0.00057 | Genetic Interactions | Lin-Smith-2010 |
| NUB1 | ELOVL7 | 0.000672 | Genetic Interactions | Lin-Smith-2010 |
| RAB3C | NAALADL2 | 0.000352 | Genetic Interactions | Lin-Smith-2010 |
| RAB3C | SLC9A9 | 0.000311 | Genetic Interactions | Lin-Smith-2010 |
| RAB3C | MED12L | 0.000314 | Genetic Interactions | Lin-Smith-2010 |
| RAB3C | CYP51A1 | 0.000327 | Genetic Interactions | Lin-Smith-2010 |
| RAB3C | KMT2C | 0.000473 | Genetic Interactions | Lin-Smith-2010 |
| RAB3C | TSHZ2 | 0.000284 | Genetic Interactions | Lin-Smith-2010 |
| RAB3C | NUB1 | 0.00055 | Genetic Interactions | Lin-Smith-2010 |
| P4HA2 | PPM1L | 0.000965 | Genetic Interactions | Lin-Smith-2010 |
| DNPEP | NAALADL2 | 0.000738 | Genetic Interactions | Lin-Smith-2010 |
| DNPEP | MED12L | 0.000659 | Genetic Interactions | Lin-Smith-2010 |
| DNPEP | PPM1L | 0.000646 | Genetic Interactions | Lin-Smith-2010 |
| SAMD4A | SLC9A9 | 0.000441 | Genetic Interactions | Lin-Smith-2010 |
| SAMD4A | MED12L | 0.000446 | Genetic Interactions | Lin-Smith-2010 |
| SAMD4A | ELOVL7 | 0.000589 | Genetic Interactions | Lin-Smith-2010 |
| SAMD4A | NUB1 | 0.00078 | Genetic Interactions | Lin-Smith-2010 |
| SHPK | NAALADL2 | 0.001741 | Genetic Interactions | Lin-Smith-2010 |
| SHPK | SLC9A9 | 0.001539 | Genetic Interactions | Lin-Smith-2010 |
| SHPK | MED12L | 0.001555 | Genetic Interactions | Lin-Smith-2010 |
| ACTR3B | NAALADL2 | 0.001162 | Genetic Interactions | Lin-Smith-2010 |
| REXO2 | MED12L | 0.000789 | Genetic Interactions | Lin-Smith-2010 |
| CAMKK1 | SLC9A9 | 0.000648 | Genetic Interactions | Lin-Smith-2010 |
| CAMKK1 | SAMD4A | 0.001004 | Genetic Interactions | Lin-Smith-2010 |
| RBM26 | CYP51A1 | 0.000377 | Genetic Interactions | Lin-Smith-2010 |
| RBM26 | TSHZ2 | 0.000328 | Genetic Interactions | Lin-Smith-2010 |
| DTNA | NXPE2 | 0.000539 | Genetic Interactions | Lin-Smith-2010 |
| DTNA | NAALADL2 | 0.000297 | Genetic Interactions | Lin-Smith-2010 |
| DTNA | SLC9A9 | 0.000262 | Genetic Interactions | Lin-Smith-2010 |
| DTNA | MED12L | 0.000265 | Genetic Interactions | Lin-Smith-2010 |
| DTNA | CYP51A1 | 0.000276 | Genetic Interactions | Lin-Smith-2010 |
| DTNA | TSHZ2 | 0.00024 | Genetic Interactions | Lin-Smith-2010 |
| DTNA | RAB3C | 0.000286 | Genetic Interactions | Lin-Smith-2010 |
| DTNA | RBM26 | 0.00033 | Genetic Interactions | Lin-Smith-2010 |
| CTNS | PPM1L | 0.001266 | Genetic Interactions | Lin-Smith-2010 |
| KIF13A | NAALADL2 | 0.000439 | Genetic Interactions | Lin-Smith-2010 |
| KIF13A | MED12L | 0.000393 | Genetic Interactions | Lin-Smith-2010 |
| KIF13A | TSHZ2 | 0.000356 | Genetic Interactions | Lin-Smith-2010 |
| MCF2L2 | TSHZ2 | 0.000692 | Genetic Interactions | Lin-Smith-2010 |
| COLEC12 | NAALADL2 | 0.000577 | Genetic Interactions | Lin-Smith-2010 |
| COLEC12 | SAMD4A | 0.00079 | Genetic Interactions | Lin-Smith-2010 |
| CADM1 | NAALADL2 | 0.000286 | Genetic Interactions | Lin-Smith-2010 |
| CADM1 | SLC9A9 | 0.000252 | Genetic Interactions | Lin-Smith-2010 |
| CADM1 | GALNTL5 | 0.000756 | Genetic Interactions | Lin-Smith-2010 |
| CADM1 | MED12L | 0.000255 | Genetic Interactions | Lin-Smith-2010 |
| CADM1 | ELOVL7 | 0.000337 | Genetic Interactions | Lin-Smith-2010 |
| CADM1 | TSHZ2 | 0.000231 | Genetic Interactions | Lin-Smith-2010 |
| CADM1 | NUB1 | 0.000446 | Genetic Interactions | Lin-Smith-2010 |
| CADM1 | TAX1BP3 | 0.000891 | Genetic Interactions | Lin-Smith-2010 |
| CADM1 | RAB3C | 0.000275 | Genetic Interactions | Lin-Smith-2010 |
| CADM1 | SAMD4A | 0.000391 | Genetic Interactions | Lin-Smith-2010 |
| CADM1 | RBM26 | 0.000318 | Genetic Interactions | Lin-Smith-2010 |
| CADM1 | DTNA | 0.000232 | Genetic Interactions | Lin-Smith-2010 |
| ADAM12 | NXPE2 | 0.000689 | Genetic Interactions | Lin-Smith-2010 |
| ADAM12 | NAALADL2 | 0.000379 | Genetic Interactions | Lin-Smith-2010 |
| ADAM12 | SLC9A9 | 0.000335 | Genetic Interactions | Lin-Smith-2010 |
| ADAM12 | MED12L | 0.000339 | Genetic Interactions | Lin-Smith-2010 |
| ADAM12 | CYP51A1 | 0.000352 | Genetic Interactions | Lin-Smith-2010 |
| ADAM12 | RAB3C | 0.000366 | Genetic Interactions | Lin-Smith-2010 |
| ADAM12 | DTNA | 0.000309 | Genetic Interactions | Lin-Smith-2010 |
| ADAM12 | CADM1 | 0.000297 | Genetic Interactions | Lin-Smith-2010 |
| SLC23A2 | CAMKK1 | 0.001762 | Genetic Interactions | Lin-Smith-2010 |
| SLC23A2 | KIF13A | 0.001055 | Genetic Interactions | Lin-Smith-2010 |
| PRKAG2 | NAALADL2 | 0.000502 | Genetic Interactions | Lin-Smith-2010 |
| PRKAG2 | MED12L | 0.000448 | Genetic Interactions | Lin-Smith-2010 |
| PRKAG2 | ELOVL7 | 0.000592 | Genetic Interactions | Lin-Smith-2010 |
| PRKAG2 | DTNA | 0.000408 | Genetic Interactions | Lin-Smith-2010 |
| PRKAG2 | CTNS | 0.001991 | Genetic Interactions | Lin-Smith-2010 |
| PRKAG2 | CADM1 | 0.000393 | Genetic Interactions | Lin-Smith-2010 |
| HOOK1 | NXPE2 | 0.001253 | Genetic Interactions | Lin-Smith-2010 |
| HOOK1 | KIF13A | 0.000832 | Genetic Interactions | Lin-Smith-2010 |
| HTR3A | KMT2C | 0.001589 | Genetic Interactions | Lin-Smith-2010 |
| HTR3A | ACTR3B | 0.003769 | Genetic Interactions | Lin-Smith-2010 |
| RAP1GAP2 | SLC9A9 | 0.000379 | Genetic Interactions | Lin-Smith-2010 |
| RAP1GAP2 | GALNTL5 | 0.001134 | Genetic Interactions | Lin-Smith-2010 |
| RAP1GAP2 | ACTR3B | 0.001366 | Genetic Interactions | Lin-Smith-2010 |
| RAP1GAP2 | CADM1 | 0.000336 | Genetic Interactions | Lin-Smith-2010 |
| PPP1R16B | NAALADL2 | 0.000701 | Genetic Interactions | Lin-Smith-2010 |
| PPP1R16B | RAB3C | 0.000677 | Genetic Interactions | Lin-Smith-2010 |
| ELK3 | ZNF648 | 0.001118 | Genetic Interactions | Lin-Smith-2010 |
| ELK3 | SLC9A9 | 0.000555 | Genetic Interactions | Lin-Smith-2010 |
| ELK3 | MED12L | 0.000561 | Genetic Interactions | Lin-Smith-2010 |
| ELK3 | RAB3C | 0.000606 | Genetic Interactions | Lin-Smith-2010 |
| ELK3 | DTNA | 0.000511 | Genetic Interactions | Lin-Smith-2010 |
| ELK3 | GALNT11 | 0.001887 | Genetic Interactions | Lin-Smith-2010 |
| PRPF18 | SLC9A9 | 0.000524 | Genetic Interactions | Lin-Smith-2010 |
| PRPF18 | DNPEP | 0.0012 | Genetic Interactions | Lin-Smith-2010 |
| PRPF18 | DTNA | 0.000483 | Genetic Interactions | Lin-Smith-2010 |
| PRPF18 | ELK3 | 0.001022 | Genetic Interactions | Lin-Smith-2010 |
| TMX1 | CYP51A1 | 0.000673 | Genetic Interactions | Lin-Smith-2010 |
| TMX1 | TSHZ2 | 0.000586 | Genetic Interactions | Lin-Smith-2010 |
| TMX1 | NUB1 | 0.001133 | Genetic Interactions | Lin-Smith-2010 |
| TMX1 | ADAM12 | 0.000754 | Genetic Interactions | Lin-Smith-2010 |
| TMX1 | HOOK1 | 0.001372 | Genetic Interactions | Lin-Smith-2010 |
| TMX1 | RAP1GAP2 | 0.000852 | Genetic Interactions | Lin-Smith-2010 |
| MRC2 | RAB3C | 0.000429 | Genetic Interactions | Lin-Smith-2010 |
| MRC2 | CADM1 | 0.000348 | Genetic Interactions | Lin-Smith-2010 |
| MRC2 | SLC23A2 | 0.001067 | Genetic Interactions | Lin-Smith-2010 |
| BCAS1 | HTR3A | 0.003366 | Genetic Interactions | Lin-Smith-2010 |
| DACH1 | ZNF648 | 0.000561 | Genetic Interactions | Lin-Smith-2010 |
| DACH1 | CYP51A1 | 0.000292 | Genetic Interactions | Lin-Smith-2010 |
| DACH1 | KMT2C | 0.000423 | Genetic Interactions | Lin-Smith-2010 |
| DACH1 | NUB1 | 0.000492 | Genetic Interactions | Lin-Smith-2010 |
| DACH1 | SHPK | 0.001504 | Genetic Interactions | Lin-Smith-2010 |
| DACH1 | DTNA | 0.000256 | Genetic Interactions | Lin-Smith-2010 |
| DACH1 | KIF13A | 0.00038 | Genetic Interactions | Lin-Smith-2010 |
| DACH1 | MCF2L2 | 0.000739 | Genetic Interactions | Lin-Smith-2010 |
| DACH1 | MRC2 | 0.000384 | Genetic Interactions | Lin-Smith-2010 |
| LYZ | RAB3C | 0.001459 | Genetic Interactions | Lin-Smith-2010 |
| NTHL1 | ADAM12 | 0.000806 | Genetic Interactions | Lin-Smith-2010 |
| LRBA | GLIS1 | 0.00054 | Genetic Interactions | Lin-Smith-2010 |
| LRBA | CRYGN | 0.000495 | Genetic Interactions | Lin-Smith-2010 |
| LRBA | MED12L | 0.000235 | Genetic Interactions | Lin-Smith-2010 |
| LRBA | PPM1L | 0.000231 | Genetic Interactions | Lin-Smith-2010 |
| LRBA | ELOVL7 | 0.000311 | Genetic Interactions | Lin-Smith-2010 |
| LRBA | CYP51A1 | 0.000245 | Genetic Interactions | Lin-Smith-2010 |
| LRBA | TSHZ2 | 0.000213 | Genetic Interactions | Lin-Smith-2010 |
| LRBA | ACTR3B | 0.000839 | Genetic Interactions | Lin-Smith-2010 |
| LRBA | DTNA | 0.000214 | Genetic Interactions | Lin-Smith-2010 |
| LRBA | CTNS | 0.001044 | Genetic Interactions | Lin-Smith-2010 |
| LRBA | CADM1 | 0.000206 | Genetic Interactions | Lin-Smith-2010 |
| LRBA | ADAM12 | 0.000274 | Genetic Interactions | Lin-Smith-2010 |
| LRBA | PRKAG2 | 0.000362 | Genetic Interactions | Lin-Smith-2010 |
| LRBA | HOOK1 | 0.000498 | Genetic Interactions | Lin-Smith-2010 |
| LRBA | PPP1R16B | 0.000507 | Genetic Interactions | Lin-Smith-2010 |
| LRBA | TMX1 | 0.000523 | Genetic Interactions | Lin-Smith-2010 |
| LRBA | MRC2 | 0.000321 | Genetic Interactions | Lin-Smith-2010 |
| LRBA | PLS1 | 0.000835 | Genetic Interactions | Lin-Smith-2010 |
| RHEB | ELOVL7 | 0.001633 | Genetic Interactions | Lin-Smith-2010 |
| RHEB | CYP51A1 | 0.001285 | Genetic Interactions | Lin-Smith-2010 |
| ALDH7A1 | TSHZ2 | 0.00042 | Genetic Interactions | Lin-Smith-2010 |
| ALDH7A1 | CADM1 | 0.000407 | Genetic Interactions | Lin-Smith-2010 |
| ALDH7A1 | MRC2 | 0.000634 | Genetic Interactions | Lin-Smith-2010 |
| RAI14 | GALNTL5 | 0.000969 | Genetic Interactions | Lin-Smith-2010 |
| RAI14 | PPM1L | 0.000321 | Genetic Interactions | Lin-Smith-2010 |
| RAI14 | TSHZ2 | 0.000296 | Genetic Interactions | Lin-Smith-2010 |
| RAI14 | SAMD4A | 0.000501 | Genetic Interactions | Lin-Smith-2010 |
| RAI14 | CAMKK1 | 0.000737 | Genetic Interactions | Lin-Smith-2010 |
| RAI14 | CTNS | 0.001453 | Genetic Interactions | Lin-Smith-2010 |
| RAI14 | RAP1GAP2 | 0.00043 | Genetic Interactions | Lin-Smith-2010 |
| RAI14 | LRBA | 0.000264 | Genetic Interactions | Lin-Smith-2010 |
| ZNF217 | SLC9A9 | 0.000393 | Genetic Interactions | Lin-Smith-2010 |
| ZNF217 | CYP51A1 | 0.000413 | Genetic Interactions | Lin-Smith-2010 |
| ZNF217 | KIF13A | 0.000536 | Genetic Interactions | Lin-Smith-2010 |
| ZNF217 | CADM1 | 0.000348 | Genetic Interactions | Lin-Smith-2010 |
| ZNF217 | ELK3 | 0.000767 | Genetic Interactions | Lin-Smith-2010 |
| ZNF217 | RAI14 | 0.000447 | Genetic Interactions | Lin-Smith-2010 |
| ANK3 | NAALADL2 | 0.000264 | Genetic Interactions | Lin-Smith-2010 |
| ANK3 | SLC9A9 | 0.000233 | Genetic Interactions | Lin-Smith-2010 |
| ANK3 | GALNTL5 | 0.000698 | Genetic Interactions | Lin-Smith-2010 |
| ANK3 | PPM1L | 0.000231 | Genetic Interactions | Lin-Smith-2010 |
| ANK3 | ELOVL7 | 0.000311 | Genetic Interactions | Lin-Smith-2010 |
| ANK3 | TSHZ2 | 0.000213 | Genetic Interactions | Lin-Smith-2010 |
| ANK3 | NUB1 | 0.000412 | Genetic Interactions | Lin-Smith-2010 |
| ANK3 | TAX1BP3 | 0.000823 | Genetic Interactions | Lin-Smith-2010 |
| ANK3 | CAMKK1 | 0.000531 | Genetic Interactions | Lin-Smith-2010 |
| ANK3 | RBM26 | 0.000293 | Genetic Interactions | Lin-Smith-2010 |
| ANK3 | DTNA | 0.000215 | Genetic Interactions | Lin-Smith-2010 |
| ANK3 | KIF13A | 0.000318 | Genetic Interactions | Lin-Smith-2010 |
| ANK3 | HOOK1 | 0.000499 | Genetic Interactions | Lin-Smith-2010 |
| ANK3 | ELK3 | 0.000454 | Genetic Interactions | Lin-Smith-2010 |
| ANK3 | PLS1 | 0.000836 | Genetic Interactions | Lin-Smith-2010 |
| ANK3 | DACH1 | 0.000228 | Genetic Interactions | Lin-Smith-2010 |
| ANK3 | LRBA | 0.00019 | Genetic Interactions | Lin-Smith-2010 |
| ANK3 | ZNF217 | 0.000322 | Genetic Interactions | Lin-Smith-2010 |
| ITGAE | GALNT11 | 0.004334 | Genetic Interactions | Lin-Smith-2010 |
| ITGAE | PRKAG2 | 0.001986 | Genetic Interactions | Lin-Smith-2010 |
| ITGAE | ANK3 | 0.001043 | Genetic Interactions | Lin-Smith-2010 |
| ASPA | LRBA | 0.001421 | Genetic Interactions | Lin-Smith-2010 |
| SMARCD3 | SLC9A9 | 0.002118 | Genetic Interactions | Lin-Smith-2010 |
| CYP2J2 | SLC9A9 | 0.00091 | Genetic Interactions | Lin-Smith-2010 |
| MRPL3 | TRPV3 | 0.002657 | Genetic Interactions | Lin-Smith-2010 |
| MRPL3 | RAB3C | 0.000589 | Genetic Interactions | Lin-Smith-2010 |
| MRPL3 | RBM26 | 0.000679 | Genetic Interactions | Lin-Smith-2010 |
| MRPL3 | CADM1 | 0.000478 | Genetic Interactions | Lin-Smith-2010 |
| MRPL3 | SLC23A2 | 0.001465 | Genetic Interactions | Lin-Smith-2010 |
| MRPL3 | MRC2 | 0.000744 | Genetic Interactions | Lin-Smith-2010 |
| MRPL3 | DACH1 | 0.000527 | Genetic Interactions | Lin-Smith-2010 |
| MRPL3 | LYZ | 0.002533 | Genetic Interactions | Lin-Smith-2010 |
| MRPL3 | ALDH7A1 | 0.00087 | Genetic Interactions | Lin-Smith-2010 |
| MRPL3 | ANK3 | 0.000441 | Genetic Interactions | Lin-Smith-2010 |
| TSC2 | NAALADL2 | 0.000718 | Genetic Interactions | Lin-Smith-2010 |
| TSC2 | SPATA22 | 0.005821 | Genetic Interactions | Lin-Smith-2010 |
| TSC2 | ADAM12 | 0.000747 | Genetic Interactions | Lin-Smith-2010 |
| TSC2 | BCAS1 | 0.002045 | Genetic Interactions | Lin-Smith-2010 |
| DAB1 | NXPE2 | 0.000454 | Genetic Interactions | Lin-Smith-2010 |
| DAB1 | SLC9A9 | 0.000221 | Genetic Interactions | Lin-Smith-2010 |
| DAB1 | PPM1L | 0.000219 | Genetic Interactions | Lin-Smith-2010 |
| DAB1 | ELOVL7 | 0.000295 | Genetic Interactions | Lin-Smith-2010 |
| DAB1 | TAX1BP3 | 0.000781 | Genetic Interactions | Lin-Smith-2010 |
| DAB1 | RAB3C | 0.000241 | Genetic Interactions | Lin-Smith-2010 |
| DAB1 | RBM26 | 0.000278 | Genetic Interactions | Lin-Smith-2010 |
| DAB1 | CTNS | 0.000992 | Genetic Interactions | Lin-Smith-2010 |
| DAB1 | ADAM12 | 0.00026 | Genetic Interactions | Lin-Smith-2010 |
| DAB1 | PRKAG2 | 0.000344 | Genetic Interactions | Lin-Smith-2010 |
| DAB1 | PRPF18 | 0.000407 | Genetic Interactions | Lin-Smith-2010 |
| DAB1 | TMX1 | 0.000497 | Genetic Interactions | Lin-Smith-2010 |
| DAB1 | EMC6 | 0.001451 | Genetic Interactions | Lin-Smith-2010 |
| DAB1 | LRBA | 0.000181 | Genetic Interactions | Lin-Smith-2010 |
| DAB1 | ANK3 | 0.000181 | Genetic Interactions | Lin-Smith-2010 |
| CDH11 | TRPV3 | 0.001541 | Genetic Interactions | Lin-Smith-2010 |
| CDH11 | CRYGN | 0.000665 | Genetic Interactions | Lin-Smith-2010 |
| CDH11 | GALNTL5 | 0.000937 | Genetic Interactions | Lin-Smith-2010 |
| CDH11 | PPM1L | 0.00031 | Genetic Interactions | Lin-Smith-2010 |
| CDH11 | CYP51A1 | 0.000329 | Genetic Interactions | Lin-Smith-2010 |
| CDH11 | KMT2C | 0.000476 | Genetic Interactions | Lin-Smith-2010 |
| CDH11 | TSHZ2 | 0.000286 | Genetic Interactions | Lin-Smith-2010 |
| CDH11 | RAB3C | 0.000341 | Genetic Interactions | Lin-Smith-2010 |
| CDH11 | SAMD4A | 0.000484 | Genetic Interactions | Lin-Smith-2010 |
| CDH11 | CAMKK1 | 0.000713 | Genetic Interactions | Lin-Smith-2010 |
| CDH11 | RBM26 | 0.000394 | Genetic Interactions | Lin-Smith-2010 |
| CDH11 | KIF13A | 0.000427 | Genetic Interactions | Lin-Smith-2010 |
| CDH11 | COLEC12 | 0.00056 | Genetic Interactions | Lin-Smith-2010 |
| CDH11 | CADM1 | 0.000277 | Genetic Interactions | Lin-Smith-2010 |
| CDH11 | ADAM12 | 0.000368 | Genetic Interactions | Lin-Smith-2010 |
| CDH11 | SLC23A2 | 0.00085 | Genetic Interactions | Lin-Smith-2010 |
| CDH11 | PPP1R16B | 0.000681 | Genetic Interactions | Lin-Smith-2010 |
| CDH11 | LRBA | 0.000256 | Genetic Interactions | Lin-Smith-2010 |
| CDH11 | RAI14 | 0.000356 | Genetic Interactions | Lin-Smith-2010 |
| CDH11 | ANK3 | 0.000256 | Genetic Interactions | Lin-Smith-2010 |
| CDH11 | DAB1 | 0.000243 | Genetic Interactions | Lin-Smith-2010 |
| CPE | SLC9A9 | 0.000289 | Genetic Interactions | Lin-Smith-2010 |
| CPE | CYP51A1 | 0.000304 | Genetic Interactions | Lin-Smith-2010 |
| CPE | TSHZ2 | 0.000265 | Genetic Interactions | Lin-Smith-2010 |
| CPE | KIF13A | 0.000394 | Genetic Interactions | Lin-Smith-2010 |
| CPE | CADM1 | 0.000256 | Genetic Interactions | Lin-Smith-2010 |
| CPE | ANK3 | 0.000236 | Genetic Interactions | Lin-Smith-2010 |
| CPE | MRPL3 | 0.000547 | Genetic Interactions | Lin-Smith-2010 |
| CPE | DAB1 | 0.000224 | Genetic Interactions | Lin-Smith-2010 |
| PAFAH1B1 | KMT2C | 0.001168 | Genetic Interactions | Lin-Smith-2010 |
| PAFAH1B1 | NUB1 | 0.001359 | Genetic Interactions | Lin-Smith-2010 |
| PAFAH1B1 | CADM1 | 0.000681 | Genetic Interactions | Lin-Smith-2010 |
| PAFAH1B1 | ELK3 | 0.001498 | Genetic Interactions | Lin-Smith-2010 |
| PAFAH1B1 | PRPF18 | 0.001414 | Genetic Interactions | Lin-Smith-2010 |
| MECOM | GALNTL5 | 0.000695 | Genetic Interactions | Lin-Smith-2010 |
| MECOM | CYP51A1 | 0.000244 | Genetic Interactions | Lin-Smith-2010 |
| MECOM | TSHZ2 | 0.000212 | Genetic Interactions | Lin-Smith-2010 |
| MECOM | RAB3C | 0.000253 | Genetic Interactions | Lin-Smith-2010 |
| MECOM | DNPEP | 0.000531 | Genetic Interactions | Lin-Smith-2010 |
| MECOM | SHPK | 0.001254 | Genetic Interactions | Lin-Smith-2010 |
| MECOM | CAMKK1 | 0.000529 | Genetic Interactions | Lin-Smith-2010 |
| MECOM | RBM26 | 0.000292 | Genetic Interactions | Lin-Smith-2010 |
| MECOM | GALNT11 | 0.000789 | Genetic Interactions | Lin-Smith-2010 |
| MECOM | KIF13A | 0.000316 | Genetic Interactions | Lin-Smith-2010 |
| MECOM | ADAM12 | 0.000273 | Genetic Interactions | Lin-Smith-2010 |
| MECOM | PRKAG2 | 0.000361 | Genetic Interactions | Lin-Smith-2010 |
| MECOM | HOOK1 | 0.000497 | Genetic Interactions | Lin-Smith-2010 |
| MECOM | RAP1GAP2 | 0.000309 | Genetic Interactions | Lin-Smith-2010 |
| MECOM | PPP1R16B | 0.000505 | Genetic Interactions | Lin-Smith-2010 |
| MECOM | DACH1 | 0.000227 | Genetic Interactions | Lin-Smith-2010 |
| MECOM | LRBA | 0.00019 | Genetic Interactions | Lin-Smith-2010 |
| MECOM | ALDH7A1 | 0.000374 | Genetic Interactions | Lin-Smith-2010 |
| MECOM | RAI14 | 0.000264 | Genetic Interactions | Lin-Smith-2010 |
| MECOM | CPE | 0.000235 | Genetic Interactions | Lin-Smith-2010 |
| RAB11FIP3 | KMT2C | 0.001272 | Genetic Interactions | Lin-Smith-2010 |
| RAB11FIP3 | HOOK1 | 0.001791 | Genetic Interactions | Lin-Smith-2010 |
| RAB11FIP3 | RHEB | 0.003592 | Genetic Interactions | Lin-Smith-2010 |
| RAB11FIP3 | DAB1 | 0.000649 | Genetic Interactions | Lin-Smith-2010 |
| RAB11FIP3 | MECOM | 0.000682 | Genetic Interactions | Lin-Smith-2010 |
| BNIP3 | GLIS1 | 0.000949 | Genetic Interactions | Lin-Smith-2010 |
| BNIP3 | SLC9A9 | 0.000409 | Genetic Interactions | Lin-Smith-2010 |
| BNIP3 | MED12L | 0.000413 | Genetic Interactions | Lin-Smith-2010 |
| BNIP3 | ELOVL7 | 0.000546 | Genetic Interactions | Lin-Smith-2010 |
| BNIP3 | RAB3C | 0.000446 | Genetic Interactions | Lin-Smith-2010 |
| BNIP3 | SAMD4A | 0.000633 | Genetic Interactions | Lin-Smith-2010 |
| BNIP3 | CAMKK1 | 0.000931 | Genetic Interactions | Lin-Smith-2010 |
| BNIP3 | KIF13A | 0.000557 | Genetic Interactions | Lin-Smith-2010 |
| BNIP3 | COLEC12 | 0.000732 | Genetic Interactions | Lin-Smith-2010 |
| BNIP3 | RAP1GAP2 | 0.000544 | Genetic Interactions | Lin-Smith-2010 |
| BNIP3 | TMX1 | 0.000919 | Genetic Interactions | Lin-Smith-2010 |
| BNIP3 | MRC2 | 0.000564 | Genetic Interactions | Lin-Smith-2010 |
| BNIP3 | MRPL3 | 0.000774 | Genetic Interactions | Lin-Smith-2010 |
| BNIP3 | DAB1 | 0.000317 | Genetic Interactions | Lin-Smith-2010 |
| BNIP3 | CPE | 0.000415 | Genetic Interactions | Lin-Smith-2010 |
| CTLA4 | PRPF18 | 0.002804 | Genetic Interactions | Lin-Smith-2010 |
| CTLA4 | MECOM | 0.001241 | Genetic Interactions | Lin-Smith-2010 |
| RPH3AL | KMT2C | 0.000513 | Genetic Interactions | Lin-Smith-2010 |
| RPH3AL | TSHZ2 | 0.000309 | Genetic Interactions | Lin-Smith-2010 |
| RPH3AL | NUB1 | 0.000596 | Genetic Interactions | Lin-Smith-2010 |
| RPH3AL | BCAS1 | 0.001086 | Genetic Interactions | Lin-Smith-2010 |
| RPH3AL | LRBA | 0.000276 | Genetic Interactions | Lin-Smith-2010 |
| RPH3AL | RAI14 | 0.000383 | Genetic Interactions | Lin-Smith-2010 |
| RPH3AL | ANK3 | 0.000276 | Genetic Interactions | Lin-Smith-2010 |
| RPH3AL | CPE | 0.000342 | Genetic Interactions | Lin-Smith-2010 |
| RPH3AL | RAB11FIP3 | 0.00099 | Genetic Interactions | Lin-Smith-2010 |
| AKAP5 | RAB3C | 0.000727 | Genetic Interactions | Lin-Smith-2010 |
| AKAP5 | SHPK | 0.0036 | Genetic Interactions | Lin-Smith-2010 |
| AKAP5 | RBM26 | 0.000839 | Genetic Interactions | Lin-Smith-2010 |
| AKAP5 | ALDH7A1 | 0.001074 | Genetic Interactions | Lin-Smith-2010 |
| AKAP5 | ANK3 | 0.000545 | Genetic Interactions | Lin-Smith-2010 |
| AKAP5 | MRPL3 | 0.001262 | Genetic Interactions | Lin-Smith-2010 |
| AKAP5 | BNIP3 | 0.000956 | Genetic Interactions | Lin-Smith-2010 |
| SIX1 | NXPE2 | 0.001217 | Genetic Interactions | Lin-Smith-2010 |
| SIX1 | RAB3C | 0.000646 | Genetic Interactions | Lin-Smith-2010 |
| SIX1 | RBM26 | 0.000746 | Genetic Interactions | Lin-Smith-2010 |
| SIX1 | COLEC12 | 0.001061 | Genetic Interactions | Lin-Smith-2010 |
| SIX1 | CADM1 | 0.000525 | Genetic Interactions | Lin-Smith-2010 |
| SIX1 | CPE | 0.000601 | Genetic Interactions | Lin-Smith-2010 |
| SIX1 | BNIP3 | 0.00085 | Genetic Interactions | Lin-Smith-2010 |
| DAB2IP | NAALADL2 | 0.000397 | Genetic Interactions | Lin-Smith-2010 |
| DAB2IP | SLC9A9 | 0.00035 | Genetic Interactions | Lin-Smith-2010 |
| DAB2IP | MED12L | 0.000354 | Genetic Interactions | Lin-Smith-2010 |
| DAB2IP | ELOVL7 | 0.000468 | Genetic Interactions | Lin-Smith-2010 |
| DAB2IP | CYP51A1 | 0.000368 | Genetic Interactions | Lin-Smith-2010 |
| DAB2IP | RAB3C | 0.000383 | Genetic Interactions | Lin-Smith-2010 |
| DAB2IP | RBM26 | 0.000441 | Genetic Interactions | Lin-Smith-2010 |
| DAB2IP | COLEC12 | 0.000628 | Genetic Interactions | Lin-Smith-2010 |
| DAB2IP | ADAM12 | 0.000412 | Genetic Interactions | Lin-Smith-2010 |
| DAB2IP | HOOK1 | 0.00075 | Genetic Interactions | Lin-Smith-2010 |
| DAB2IP | ZNF217 | 0.000484 | Genetic Interactions | Lin-Smith-2010 |
| DAB2IP | ANK3 | 0.000287 | Genetic Interactions | Lin-Smith-2010 |
| DAB2IP | CDH11 | 0.000385 | Genetic Interactions | Lin-Smith-2010 |
| DAB2IP | MECOM | 0.000286 | Genetic Interactions | Lin-Smith-2010 |
| DAB2IP | SIX1 | 0.000729 | Genetic Interactions | Lin-Smith-2010 |
| PKD2 | NAALADL2 | 0.000489 | Genetic Interactions | Lin-Smith-2010 |
| PKD2 | TAX1BP3 | 0.001525 | Genetic Interactions | Lin-Smith-2010 |
| PKD2 | REXO2 | 0.001184 | Genetic Interactions | Lin-Smith-2010 |
| PKD2 | RBM26 | 0.000544 | Genetic Interactions | Lin-Smith-2010 |
| PKD2 | CADM1 | 0.000383 | Genetic Interactions | Lin-Smith-2010 |
| PKD2 | ADAM12 | 0.000508 | Genetic Interactions | Lin-Smith-2010 |
| PKD2 | ELK3 | 0.000842 | Genetic Interactions | Lin-Smith-2010 |
| PKD2 | EMC6 | 0.002834 | Genetic Interactions | Lin-Smith-2010 |
| PKD2 | LRBA | 0.000353 | Genetic Interactions | Lin-Smith-2010 |
| PKD2 | ANK3 | 0.000353 | Genetic Interactions | Lin-Smith-2010 |
| PKD2 | BNIP3 | 0.00062 | Genetic Interactions | Lin-Smith-2010 |
| PKD2 | DAB2IP | 0.000531 | Genetic Interactions | Lin-Smith-2010 |
| RNF168 | SLC9A9 | 0.000495 | Genetic Interactions | Lin-Smith-2010 |
| RNF168 | TSHZ2 | 0.000453 | Genetic Interactions | Lin-Smith-2010 |
| RNF168 | RAB3C | 0.00054 | Genetic Interactions | Lin-Smith-2010 |
| RNF168 | CAMKK1 | 0.001127 | Genetic Interactions | Lin-Smith-2010 |
| RNF168 | KIF13A | 0.000675 | Genetic Interactions | Lin-Smith-2010 |
| RNF168 | PPP1R16B | 0.001078 | Genetic Interactions | Lin-Smith-2010 |
| RNF168 | DACH1 | 0.000484 | Genetic Interactions | Lin-Smith-2010 |
| RNF168 | LRBA | 0.000404 | Genetic Interactions | Lin-Smith-2010 |
| RNF168 | RAI14 | 0.000563 | Genetic Interactions | Lin-Smith-2010 |
| RNF168 | CYP2J2 | 0.001583 | Genetic Interactions | Lin-Smith-2010 |
| RNF168 | RAB11FIP3 | 0.001454 | Genetic Interactions | Lin-Smith-2010 |
| LRP8 | RBM26 | 0.0008 | Genetic Interactions | Lin-Smith-2010 |
| LRP8 | MRC2 | 0.000877 | Genetic Interactions | Lin-Smith-2010 |
| CTNNB1 | PPM1L | 0.000278 | Genetic Interactions | Lin-Smith-2010 |
| CTNNB1 | TSHZ2 | 0.000257 | Genetic Interactions | Lin-Smith-2010 |
| CTNNB1 | RAB3C | 0.000306 | Genetic Interactions | Lin-Smith-2010 |
| CTNNB1 | DTNA | 0.000258 | Genetic Interactions | Lin-Smith-2010 |
| CTNNB1 | COLEC12 | 0.000503 | Genetic Interactions | Lin-Smith-2010 |
| CTNNB1 | NTHL1 | 0.000675 | Genetic Interactions | Lin-Smith-2010 |
| CTNNB1 | LRBA | 0.000229 | Genetic Interactions | Lin-Smith-2010 |
| CTNNB1 | TSC2 | 0.000626 | Genetic Interactions | Lin-Smith-2010 |
| CTNNB1 | CPE | 0.000285 | Genetic Interactions | Lin-Smith-2010 |
| CTNNB1 | RPH3AL | 0.000332 | Genetic Interactions | Lin-Smith-2010 |
| PAFAH1B2 | GLIS1 | 0.001103 | Genetic Interactions | Lin-Smith-2010 |
| PAFAH1B2 | NUB1 | 0.00084 | Genetic Interactions | Lin-Smith-2010 |
| PAFAH1B2 | RBM26 | 0.000598 | Genetic Interactions | Lin-Smith-2010 |
| PAFAH1B2 | LYZ | 0.002231 | Genetic Interactions | Lin-Smith-2010 |
| PAFAH1B2 | SIX1 | 0.000988 | Genetic Interactions | Lin-Smith-2010 |
| PAFAH1B2 | PKD2 | 0.00072 | Genetic Interactions | Lin-Smith-2010 |
| TSC1 | TSHZ2 | 0.000824 | Genetic Interactions | Lin-Smith-2010 |
| TSC1 | KIF13A | 0.001228 | Genetic Interactions | Lin-Smith-2010 |
| TSC1 | SLC23A2 | 0.002445 | Genetic Interactions | Lin-Smith-2010 |
| TSC1 | RAP1GAP2 | 0.001197 | Genetic Interactions | Lin-Smith-2010 |
| TSC1 | ASPA | 0.005498 | Genetic Interactions | Lin-Smith-2010 |
| ERP44 | ELOVL7 | 0.000728 | Genetic Interactions | Lin-Smith-2010 |
| ERP44 | CYP51A1 | 0.000573 | Genetic Interactions | Lin-Smith-2010 |
| ERP44 | TSHZ2 | 0.000499 | Genetic Interactions | Lin-Smith-2010 |
| ERP44 | GALNT11 | 0.001853 | Genetic Interactions | Lin-Smith-2010 |
| ERP44 | ADAM12 | 0.000641 | Genetic Interactions | Lin-Smith-2010 |
| ERP44 | PPP1R16B | 0.001187 | Genetic Interactions | Lin-Smith-2010 |
| ERP44 | LYZ | 0.00256 | Genetic Interactions | Lin-Smith-2010 |
| ERP44 | LRBA | 0.000445 | Genetic Interactions | Lin-Smith-2010 |
| ERP44 | ANK3 | 0.000446 | Genetic Interactions | Lin-Smith-2010 |
| ERP44 | ASPA | 0.003329 | Genetic Interactions | Lin-Smith-2010 |
| ERP44 | RPH3AL | 0.000645 | Genetic Interactions | Lin-Smith-2010 |
| ERP44 | PKD2 | 0.000827 | Genetic Interactions | Lin-Smith-2010 |
| NSA2 | TSHZ2 | 0.001329 | Genetic Interactions | Lin-Smith-2010 |
| NSA2 | DTNA | 0.001337 | Genetic Interactions | Lin-Smith-2010 |
| NSA2 | AKAP5 | 0.003396 | Genetic Interactions | Lin-Smith-2010 |
| AASS | SLC9A9 | 0.000405 | Genetic Interactions | Lin-Smith-2010 |
| AASS | PPM1L | 0.000401 | Genetic Interactions | Lin-Smith-2010 |
| AASS | TSHZ2 | 0.000371 | Genetic Interactions | Lin-Smith-2010 |
| AASS | DNPEP | 0.000927 | Genetic Interactions | Lin-Smith-2010 |
| AASS | CTNS | 0.001817 | Genetic Interactions | Lin-Smith-2010 |
| AASS | KIF13A | 0.000552 | Genetic Interactions | Lin-Smith-2010 |
| AASS | CADM1 | 0.000359 | Genetic Interactions | Lin-Smith-2010 |
| AASS | MRC2 | 0.000559 | Genetic Interactions | Lin-Smith-2010 |
| AASS | BCAS1 | 0.001304 | Genetic Interactions | Lin-Smith-2010 |
| AASS | LYZ | 0.001901 | Genetic Interactions | Lin-Smith-2010 |
| AASS | NTHL1 | 0.000974 | Genetic Interactions | Lin-Smith-2010 |
| AASS | LRBA | 0.000331 | Genetic Interactions | Lin-Smith-2010 |
| AASS | DAB2IP | 0.000498 | Genetic Interactions | Lin-Smith-2010 |
| AASS | RNF168 | 0.000704 | Genetic Interactions | Lin-Smith-2010 |
| CLIP1 | NXPE2 | 0.000789 | Genetic Interactions | Lin-Smith-2010 |
| CLIP1 | PPM1L | 0.00038 | Genetic Interactions | Lin-Smith-2010 |
| CLIP1 | CYP51A1 | 0.000404 | Genetic Interactions | Lin-Smith-2010 |
| CLIP1 | DNPEP | 0.000879 | Genetic Interactions | Lin-Smith-2010 |
| CLIP1 | KIF13A | 0.000524 | Genetic Interactions | Lin-Smith-2010 |
| CLIP1 | CADM1 | 0.00034 | Genetic Interactions | Lin-Smith-2010 |
| CLIP1 | ADAM12 | 0.000452 | Genetic Interactions | Lin-Smith-2010 |
| CLIP1 | HOOK1 | 0.000822 | Genetic Interactions | Lin-Smith-2010 |
| CLIP1 | MRC2 | 0.00053 | Genetic Interactions | Lin-Smith-2010 |
| CLIP1 | NTHL1 | 0.000924 | Genetic Interactions | Lin-Smith-2010 |
| CLIP1 | LRBA | 0.000314 | Genetic Interactions | Lin-Smith-2010 |
| CLIP1 | ALDH7A1 | 0.00062 | Genetic Interactions | Lin-Smith-2010 |
| CLIP1 | PAFAH1B2 | 0.000641 | Genetic Interactions | Lin-Smith-2010 |
| PRKAG2 | CAMKK1 | 0.057667 | Pathway | Wu-Stein-2010 |
| TSC2 | RHEB | 0.054244 | Pathway | Wu-Stein-2010 |
| COL1A1 | ITGAE | 0.011086 | Pathway | Wu-Stein-2010 |
| PAFAH1B1 | DAB1 | 0.014948 | Pathway | Wu-Stein-2010 |
| LRP8 | DAB1 | 0.062211 | Pathway | Wu-Stein-2010 |
| CTNNB1 | SMARCD3 | 0.012243 | Pathway | Wu-Stein-2010 |
| CTNNB1 | CDH11 | 0.002967 | Pathway | Wu-Stein-2010 |
| PAFAH1B2 | DAB1 | 0.145795 | Pathway | Wu-Stein-2010 |
| PAFAH1B2 | PAFAH1B1 | 0.040397 | Pathway | Wu-Stein-2010 |
| TSC1 | RHEB | 0.126892 | Pathway | Wu-Stein-2010 |
| TSC1 | TSC2 | 0.05615 | Pathway | Wu-Stein-2010 |
| CLIP1 | PAFAH1B1 | 0.006336 | Pathway | Wu-Stein-2010 |
| MECOM | RHEB | 0.082396 | Physical Interactions | Ivanochko-Arrowsmith-2019 |
| MECOM | AP2M1 | 0.047343 | Physical Interactions | Ivanochko-Arrowsmith-2019 |
| NTHL1 | CAMKK1 | 0.228873 | Physical Interactions | Hein-Mann-2015 |
| RNF168 | NUB1 | 0.583476 | Physical Interactions | Hein-Mann-2015 |
| PAFAH1B2 | PAFAH1B1 | 0.132685 | Physical Interactions | Hein-Mann-2015 |
| TSC1 | TSC2 | 0.134669 | Physical Interactions | Hein-Mann-2015 |
| ERP44 | KMT2C | 0.317091 | Physical Interactions | Hein-Mann-2015 |
| ERP44 | ALDH7A1 | 0.114707 | Physical Interactions | Hein-Mann-2015 |
| NSA2 | CAMKK1 | 0.463672 | Physical Interactions | Hein-Mann-2015 |
| TSC2 | RHEB | 0.155701 | Physical Interactions | IREF-bind |
| CTNNB1 | PKD1 | 0.371094 | Physical Interactions | IREF-bind |
| PAFAH1B2 | PAFAH1B1 | 1 | Physical Interactions | IREF-bind |
| TSC1 | TSC2 | 0.080551 | Physical Interactions | IREF-bind |
| RHEB | PRKAG2 | 0.046899 | Physical Interactions | IREF-reactome |
| TSC2 | PRKAG2 | 0.05206 | Physical Interactions | IREF-reactome |
| TSC2 | RHEB | 0.07216 | Physical Interactions | IREF-reactome |
| BMP1 | COL1A1 | 0.013839 | Physical Interactions | IREF-reactome |
| TSC1 | PRKAG2 | 0.069609 | Physical Interactions | IREF-reactome |
| TSC1 | RHEB | 0.096485 | Physical Interactions | IREF-reactome |
| TSC1 | TSC2 | 0.107104 | Physical Interactions | IREF-reactome |
| AASS | ALDH7A1 | 0.765367 | Physical Interactions | IREF-reactome |
| CLIP1 | PAFAH1B1 | 0.007874 | Physical Interactions | IREF-reactome |
| RHEB | PRKAG2 | 0.046899 | Physical Interactions | Vastrik-Stein-2007 |
| TSC2 | PRKAG2 | 0.05206 | Physical Interactions | Vastrik-Stein-2007 |
| TSC2 | RHEB | 0.07216 | Physical Interactions | Vastrik-Stein-2007 |
| BMP1 | COL1A1 | 0.013839 | Physical Interactions | Vastrik-Stein-2007 |
| TSC1 | PRKAG2 | 0.069609 | Physical Interactions | Vastrik-Stein-2007 |
| TSC1 | RHEB | 0.096485 | Physical Interactions | Vastrik-Stein-2007 |
| TSC1 | TSC2 | 0.107104 | Physical Interactions | Vastrik-Stein-2007 |
| AASS | ALDH7A1 | 0.765367 | Physical Interactions | Vastrik-Stein-2007 |
| CLIP1 | PAFAH1B1 | 0.007874 | Physical Interactions | Vastrik-Stein-2007 |
| AP2M1 | KIF13A | 0.160943 | Physical Interactions | Huttlin-Gygi-2015 |
| AP2M1 | COLEC12 | 0.020554 | Physical Interactions | Huttlin-Gygi-2015 |
| ANK3 | HOOK1 | 0.085664 | Physical Interactions | IREF-hprd |
| TSC2 | RHEB | 0.090463 | Physical Interactions | IREF-hprd |
| PAFAH1B1 | DAB1 | 0.039783 | Physical Interactions | IREF-hprd |
| CTLA4 | AP2M1 | 0.040065 | Physical Interactions | IREF-hprd |
| RPH3AL | RAB3C | 0.322063 | Physical Interactions | IREF-hprd |
| SIX1 | DACH1 | 0.130644 | Physical Interactions | IREF-hprd |
| DAB2IP | DAB1 | 0.257095 | Physical Interactions | IREF-hprd |
| BMP1 | COL1A1 | 0.038992 | Physical Interactions | IREF-hprd |
| PKD2 | PKD1 | 0.080622 | Physical Interactions | IREF-hprd |
| LRP8 | DAB1 | 0.068944 | Physical Interactions | IREF-hprd |
| CTNNB1 | PKD1 | 0.011546 | Physical Interactions | IREF-hprd |
| CTNNB1 | TAX1BP3 | 0.011905 | Physical Interactions | IREF-hprd |
| CTNNB1 | CDH11 | 0.067018 | Physical Interactions | IREF-hprd |
| PAFAH1B2 | PAFAH1B1 | 0.045824 | Physical Interactions | IREF-hprd |
| TSC1 | TSC2 | 0.050261 | Physical Interactions | IREF-hprd |
| CLIP1 | PAFAH1B1 | 0.098869 | Physical Interactions | IREF-hprd |
| ANK3 | HOOK1 | 0.826744 | Predicted | I2D-BioGRID-Mouse2Human |
| RAB11FIP3 | TRPV1 | 0.736288 | Predicted | I2D-BioGRID-Mouse2Human |
| BNIP3 | RHEB | 0.80472 | Predicted | I2D-BioGRID-Mouse2Human |
| CTLA4 | AP2M1 | 0.903855 | Predicted | I2D-BioGRID-Mouse2Human |
| RPH3AL | RAB3C | 0.56305 | Predicted | I2D-BioGRID-Mouse2Human |
| AKAP5 | TRPV1 | 0.469491 | Predicted | I2D-BioGRID-Mouse2Human |
| SIX1 | DACH1 | 0.4602 | Predicted | I2D-BioGRID-Mouse2Human |
| DAB2IP | DAB1 | 0.411988 | Predicted | I2D-BioGRID-Mouse2Human |
| BMP1 | COL1A1 | 0.464804 | Predicted | I2D-BioGRID-Mouse2Human |
| PKD2 | PKD1 | 0.345901 | Predicted | I2D-BioGRID-Mouse2Human |
| LRP8 | DAB1 | 0.411988 | Predicted | I2D-BioGRID-Mouse2Human |
| CTNNB1 | TAX1BP3 | 0.229084 | Predicted | I2D-BioGRID-Mouse2Human |
| TSC1 | TSC2 | 0.191235 | Predicted | I2D-BioGRID-Mouse2Human |
| CLIP1 | PAFAH1B1 | 0.301448 | Predicted | I2D-BioGRID-Mouse2Human |
| GLIS1 | ZNF648 | 0.004378 | Shared protein domains | PFAM |
| TRPV1 | TRPV3 | 0.024524 | Shared protein domains | PFAM |
| NXPE4 | NXPE2 | 0.142857 | Shared protein domains | PFAM |
| GALNT11 | GALNTL5 | 0.031975 | Shared protein domains | PFAM |
| LRBA | WDR86 | 0.004545 | Shared protein domains | PFAM |
| RHEB | RAB3C | 0.007652 | Shared protein domains | PFAM |
| RAI14 | TRPV3 | 0.016712 | Shared protein domains | PFAM |
| ZNF217 | ZNF648 | 0.004378 | Shared protein domains | PFAM |
| ZNF217 | GLIS1 | 0.004378 | Shared protein domains | PFAM |
| ANK3 | TRPV3 | 0.012227 | Shared protein domains | PFAM |
| ANK3 | RAI14 | 0.011485 | Shared protein domains | PFAM |
| CYP2J2 | CYP51A1 | 0.016431 | Shared protein domains | PFAM |
| TSC2 | RAP1GAP2 | 0.138492 | Shared protein domains | PFAM |
| PAFAH1B1 | WDR86 | 0.005672 | Shared protein domains | PFAM |
| MECOM | ZNF648 | 0.004378 | Shared protein domains | PFAM |
| MECOM | GLIS1 | 0.004378 | Shared protein domains | PFAM |
| MECOM | ZNF217 | 0.004378 | Shared protein domains | PFAM |
| CTLA4 | CADM1 | 0.005642 | Shared protein domains | PFAM |
| PKD2 | PKD1 | 0.03182 | Shared protein domains | PFAM |
| AL157392.5 | PRPF18 | 0.916633 | Shared protein domains | PFAM |
| ERP44 | TMX1 | 0.037554 | Shared protein domains | PFAM |
| MED12 | MED12L | 1 | Shared protein domains | PFAM |
