# Supplementary figures and images for: Genomic dissection of methane emission traits in cattle: A meta-GWAS and heritability analysis across populations
Source: PLoS One. 2026 Apr 10;21(4):e0344752. doi: 10.1371/journal.pone.0344752 (PMC13068272; doi:10.1371/journal.pone.0344752)

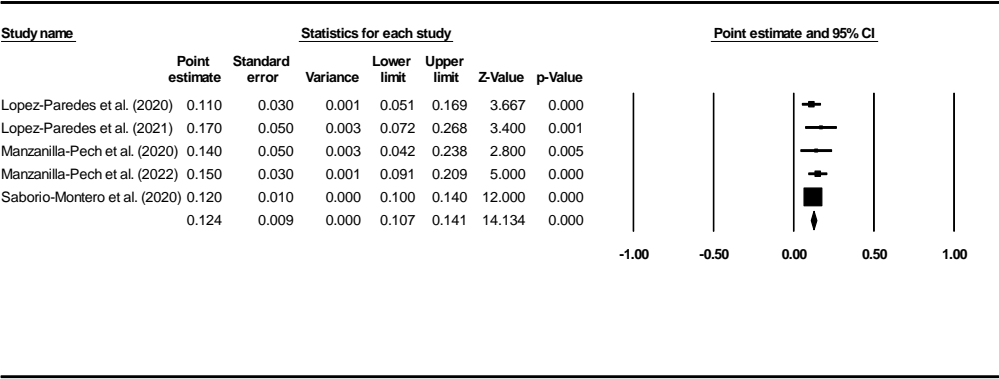

Supplement: S1 Fig — The mean effect size, calculated according to a random effects model, is indicated by the diamond at the bottom of each plot. The size of the squares illustrates the weight of each study relative to the mean effect size. Smaller squares represent less weight. The horizontal bars represent the 95% CI for the study. (PDF) [file pone.0344752.s006.pdf]

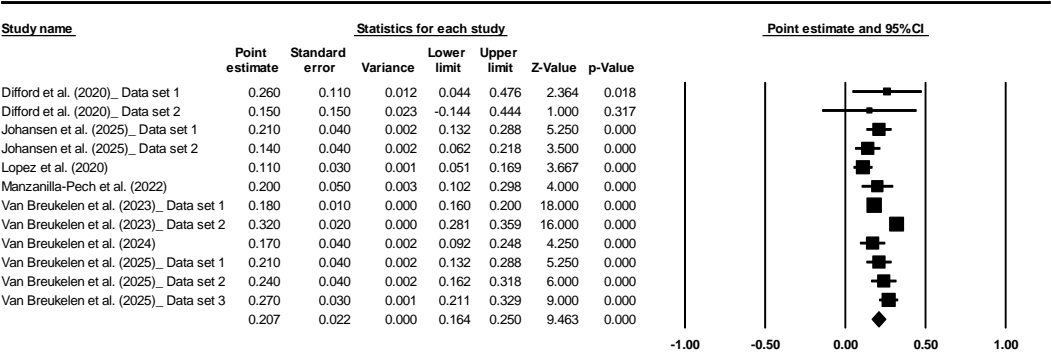

Supplement: S2 Fig — Details are provided in S1 Fig. (PDF) [file pone.0344752.s007.pdf]

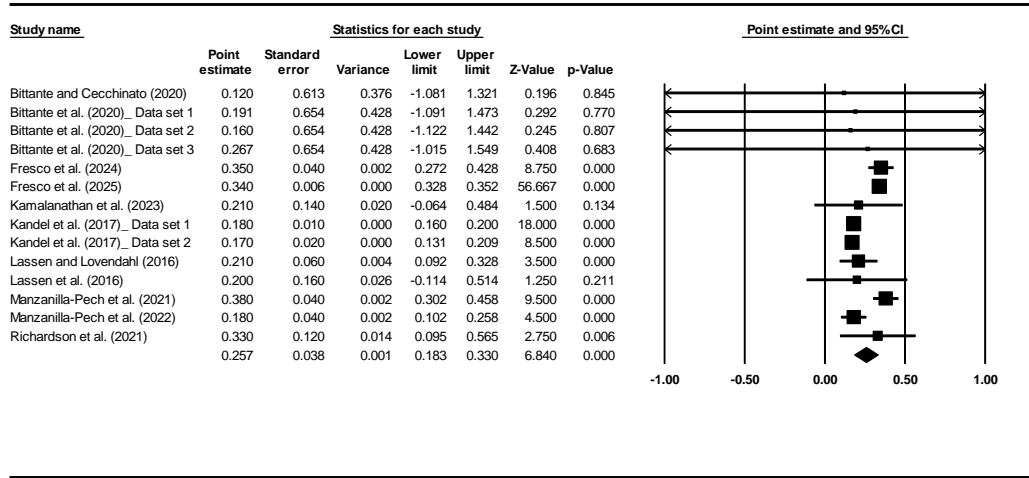

Supplement: S3 Fig — Details are provided in S1 Fig. (PDF) [file pone.0344752.s008.pdf]

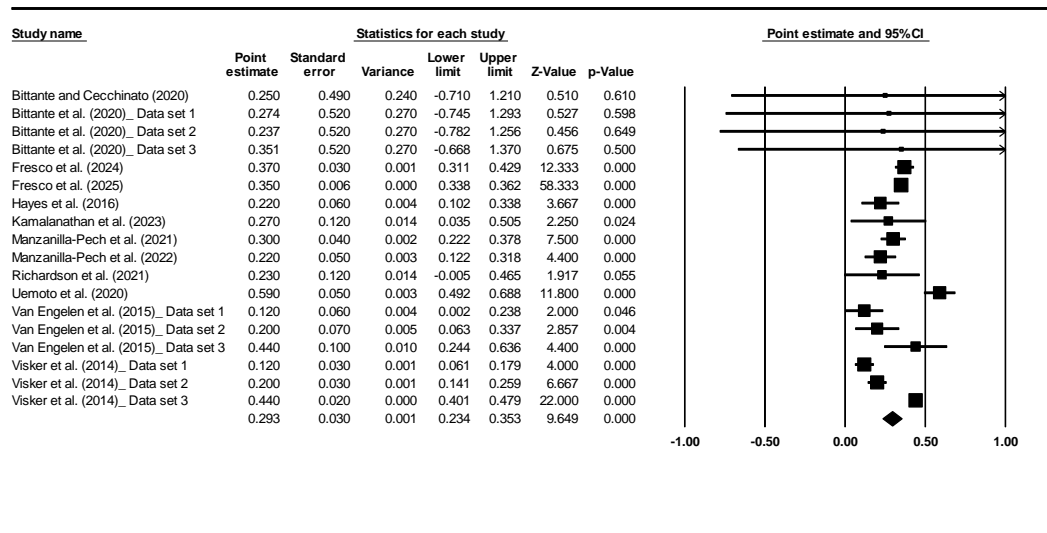

Supplement: S4 Fig — Details are provided in S1 Fig. (PDF) [file pone.0344752.s009.pdf]

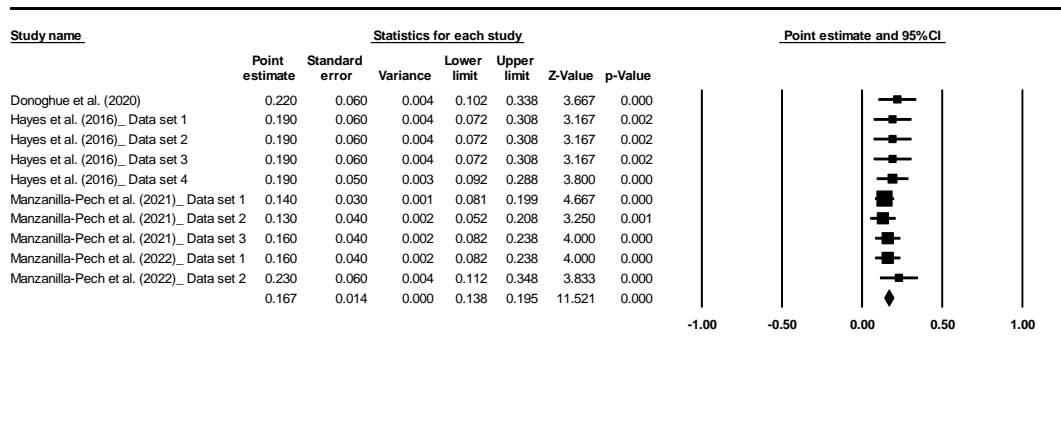

Supplement: S5 Fig — Details are provided in S1 Fig. (PDF) [file pone.0344752.s010.pdf]

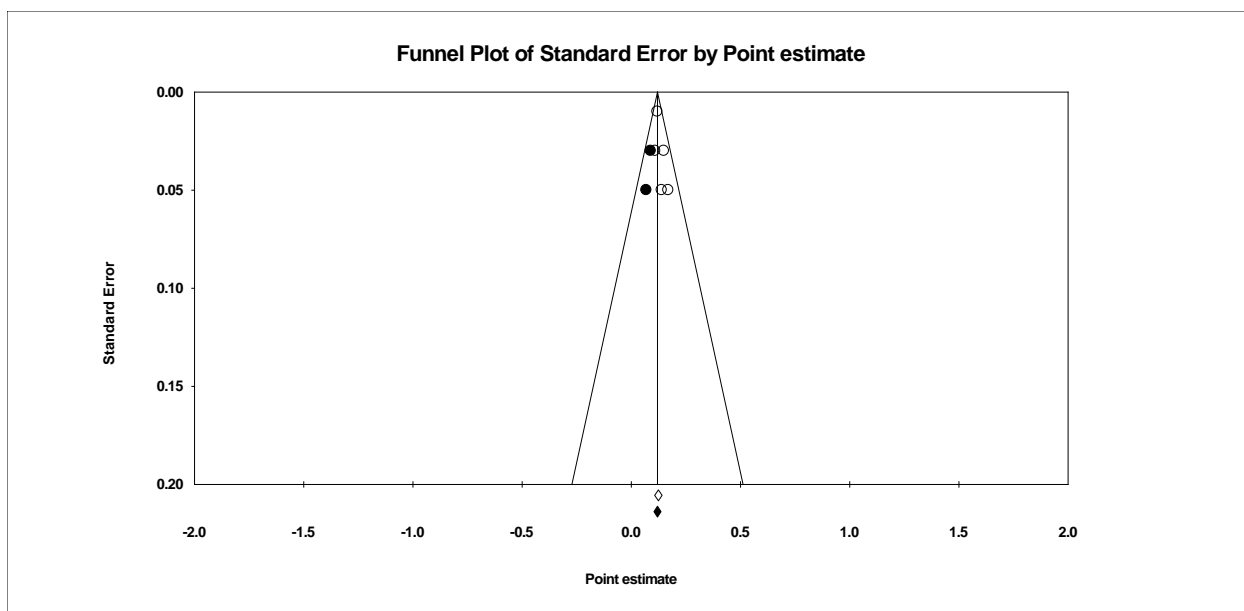

Supplement: S6 Fig — Solid dots represent the potentially missing studies that were found using the trim-and-fill method. When theoretically imputed studies are included in the meta-analysis, solid diamonds represent the mean values and CI, and open diamonds represent the mean values and confidence intervals for studies that are currently in the literature. (PDF) [file pone.0344752.s011.pdf]

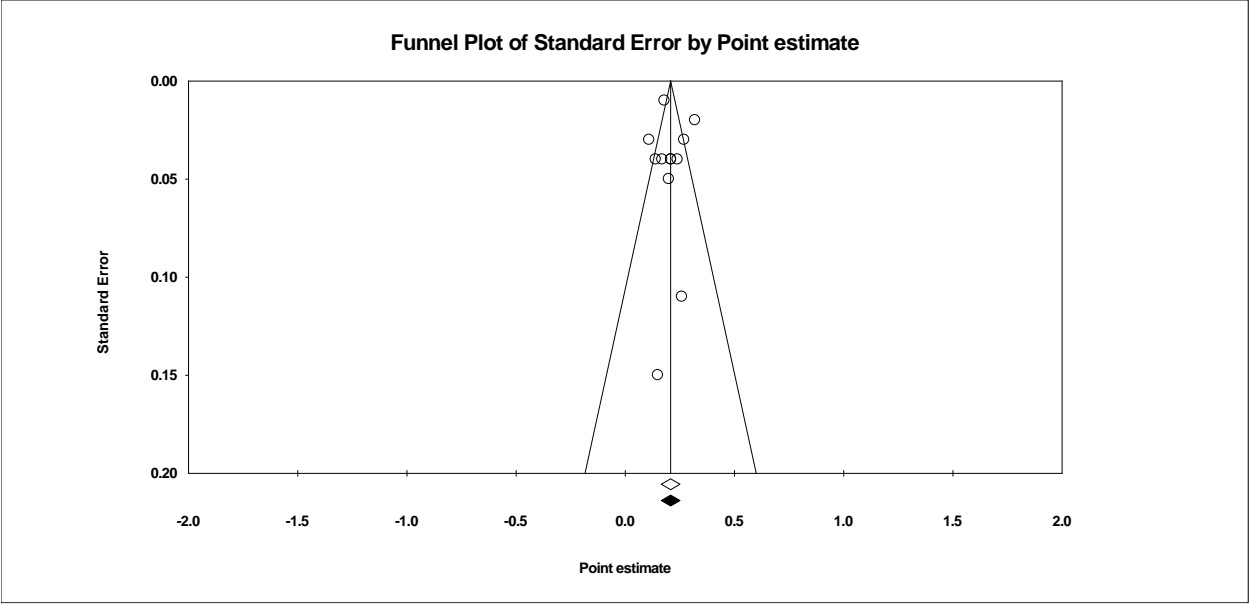

Supplement: S7 Fig — Details are provided in S6 Fig. (PDF) [file pone.0344752.s012.pdf]

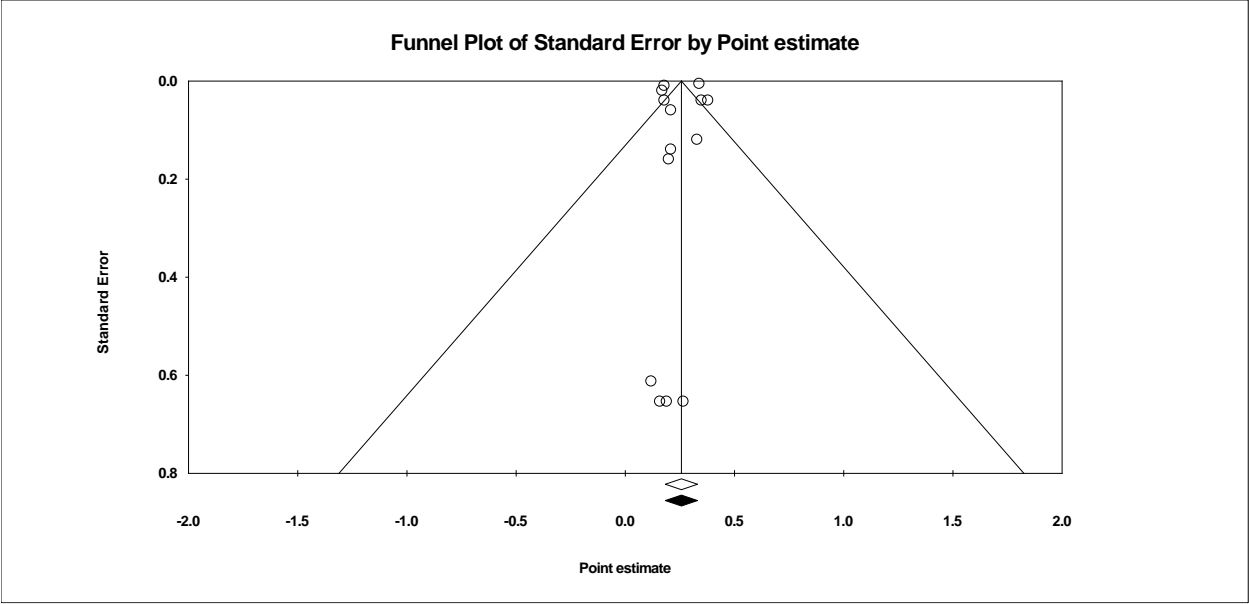

Supplement: S8 Fig — Details are provided in S6 Fig. (PDF) [file pone.0344752.s013.pdf]

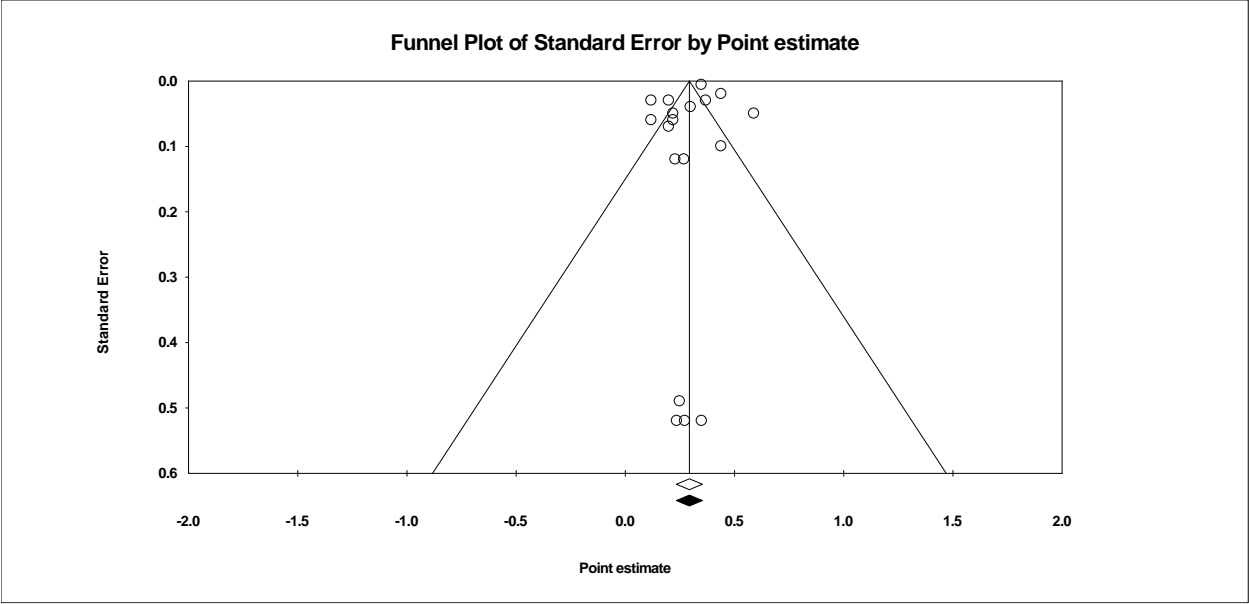

Supplement: S9 Fig — Details are provided in S6 Fig. (PDF) [file pone.0344752.s014.pdf]

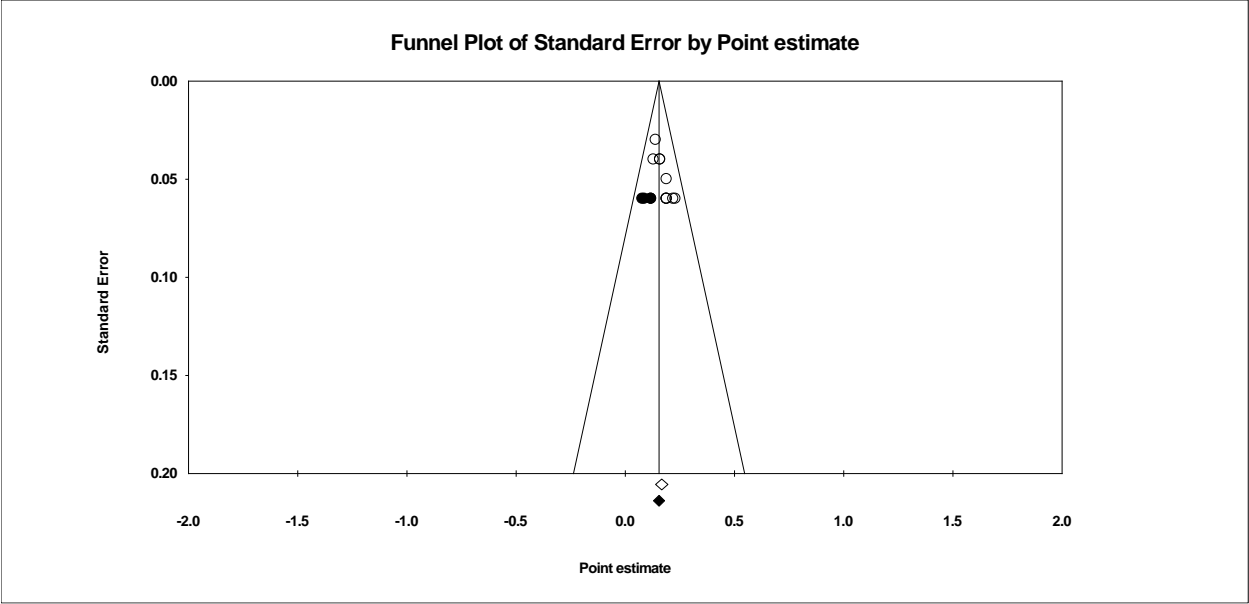

Supplement: S10 Fig — Details are provided in S6 Fig. (PDF) [file pone.0344752.s015.pdf]
