# Supplementary material for: Genomic dissection of methane emission traits in cattle: A meta-GWAS and heritability analysis across populations
Source: PLoS One. 2026 Apr 10;21(4):e0344752. doi: 10.1371/journal.pone.0344752 (PMC13068272; doi:10.1371/journal.pone.0344752)
Supplement: Attachment — Submitted filename: Response to Reviewers.docx [file pone.0344752.s019.docx]

With sincere thanks to the respective reviewers for their constructive comments, we are pointing to the changes we made to the revised manuscript. All changes were highlighted within the revised text.

**************************************************

**Reviewer #1:**

1. Grammar corrections.

**AU:** Thank you for your suggestions. All corrections were made within the revised text as suggested (Lines 21-22, 30-31, 34-35, 39, 43)

2. The self-citations by the authors should be avoided, and alternative references have been suggested.

Palangi, V., & Lackner, M. (2022). Management of enteric methane emissions in ruminants using feed additives: A review. Animals, 12(24), 3452.

Palangi, V., Taghizadeh, A., Abachi, S., & Lackner, M. (2022). Strategies to mitigate enteric methane emissions in ruminants: A review. Sustainability, 14(20), 13229.

Kader Esen, V., Palangi, V., & Esen, S. (2022). Genetic improvement and nutrigenomic management of ruminants to achieve enteric methane mitigation: A review. Methane, 1(4), 342-354.

**AU:** All suggested citations were added to the revised manuscript (Lines 720-725), but it is worth noting that the references of the authors’ works are highly relevant to the topic of this manuscript and were used in several parts of the manuscript. Therefore, we would suggest to keep them in the manuscript.

**************************************************************

**Reviewer#2:**

1. Grammar corrections.

**AU:** Thank you for your suggestions. All corrections were made within the revised text as suggested (Lines 29, 253, 603).

2. Please include a reference for this sentence

**AU:** Reference was added as recommended (Line 43).

3. Please split this into a new paragraph here.

**AU:** Reference was added as recommended (Line 49).

4. REML and Bayesian methods are estimation frameworks, whereas the animal model is a model structure that can be fitted using either approach. Consider rephrasing for clarity.

**AU:** Change made as suggested (Lines 126-128).

5. This seems redundant, as it has already been described in the M&M section. Please consider removing it.

**AU:** It was removed as suggested.

6. Please use a maximum of three decimal places

**AU:** Change made as suggested (Line 242).

7. I suggest ordering the table by chromosome position.

**AU:** Change made as suggested (Table 3).

8. could you explain what Weight means?

**AU:** “Weight indicates the total sample size contributing to the meta-analysis for each SNP, corresponding to the sample-size–weighted Z-score approach implemented in the METAL program”. This information was added to the footnote of Table 3.

9. Could you explain what the different colors in the figure mean?

**AU:** Node color intensity reflects the significance of GO term enrichment (adjusted P-value), with darker colors indicating more significant enrichment (Lines 320-322).

10. Consider not repeating in the text the values that are already clearly shown in the table, to improve readability. Please check it out in this section.

**AU:** Changes made as suggested (Lines 391-402, 422, 430, 436).

11. I suggest to include below the table the descriptions of some of the column names

**AU:** Changes made as suggested (footnotes of Tables 6 and 7).

12. Since heritability is constrained to be non-negative, it is unclear why the confidence intervals in the figure extend below zero. Please provide clarification or justify the estimation approach that leads to this.

**AU:** Some explanations were added to the text for clarification as recommended (Lines 620-625).

13. Please rewrite this sentence and avoid including results (estimated values). Try to focus on discussion and comparison of the results.

**AU:** Changes made as suggested (Lines 610-613).
